# Optic Nerve Lipidomics Reveal Impaired Glucosylsphingosine Lipids Pathway in Glaucoma

**DOI:** 10.1167/iovs.18-25802

**Published:** 2019-04

**Authors:** Muhammad Zain Chauhan, Ann-Katrin Valencia, Maria Carmen Piqueras, Mabel Enriquez-Algeciras, Sanjoy K. Bhattacharya

**Affiliations:** Department of Ophthalmology & Bascom Palmer Eye Institute, University of Miami, Miami, Florida, United States

**Keywords:** glaucoma, lipidomics, sphingolipid metabolism, glucosylsphingosine, POAG

## Abstract

**Purpose:**

To determine major differences in lipid profile between human control and glaucomatous optic nerve. To assess major enzymes in lipid pathway if aberration is revealed for a lipid class by profiling.

**Methods:**

Optic nerve (ON) samples were obtained from human cadaveric donors [control (*n* = 11) and primary open-angle glaucoma (POAG; *n* = 12)]; the lipids were extracted using Bligh and Dyer methods. Control and glaucoma donors were all Caucasians age 72.3 ± 5.9 and 70.3 ± 10.5 (inclusive of both sexes), respectively. Lipids were extracted after weighing the tissue; the protein amounts in the corresponding aqueous phase of organic solvent extraction were recorded. High-resolution mass spectrometry was performed using a Q-exactive mass spectrometer coupled with an EASY-nLC 1000 liquid chromatograph instrument. Bioinformatics and statistical analysis were performed using LipidSearch v.4.1 and MetaboAnalyst 4.0/STATA 14.2. Protein amounts were determined using Bradford's method. Western blot, ELISA, and immunohistochemistry utilized established protocols and were performed for protein quantification and localization, respectively. Additional donor tissues were utilized for Western blot, ELISA, and immunohistochemistry.

**Results:**

Principal component analysis (PCA) placed control and glaucomatous ONs in two distinct groups based on analysis of lipid profiles. Total lipid, total phospholipids, total ceramide, and total sphingolipids were similar (without significant difference) between control and glaucoma. However, we found a significant increase in glucosylsphingosine in glaucoma compared to control samples. We found similar levels of glucocerebrosidase (GBA), ceramide glucosyltransferase (UGCG), decreased nonlysosomal glucocerebrosidase (GBA2), and increased lysosomal and nonlysosomal acylsphingosine amidohydrolase (ASAH1 and ASAH2) levels in glaucomatous ON compared to control.

**Conclusions:**

We found significant differences in glucosylsphingosine lipids, consistent with decreased GBA and GBA2 and increased ASAH1 and ASAH2 immunoreactivity in glaucoma, suggesting the potential impairment of sphingolipid enzymatic pathways in lysosomal and nonlysosomal cellular compartments.

Glaucoma is a group of progressive optic neuropathies.^1^ Primary open-angle glaucoma (POAG) is one of the most common forms. Glaucoma is frequently associated with elevated intraocular pressure (IOP),^2^ but when the optic neuropathy exists in individuals with IOP in the normal range it is termed normal-tension glaucoma (NTG).^1^ The pathologies of both POAG and NTG are characterized by a progressive visual loss^3^ as a consequence of damage to optic nerve (ON) neurons or retinal ganglion cells (RGCs). The axons of RGCs traverse through the optic nerve head (ONH), which is geometrically a vulnerable structure where opposing forces maintain homeostasis and structural integrity.^4^–^6^ This region is particularly susceptible to subtle changes in biomechanics, as the ONH is a relatively weak area within a relatively strong scleral envelope.^7^ In anatomic studies, the glaucomatous ON frequently shows excavation,^8^,^9^ suggesting the exertion of a net directive force from the ONH toward the lamina cribrosa (LC).^10^ The combination of a progressive, age-related loss of visual function and excavation at the ONH suggests dynamic changes in structure coupled with a slow and progressive disintegration at the constituent molecular level.

The maintenance of ONH structural integrity, which depends upon the integrity of cells and their intercellular connectivity within the ON, is critical to visual function. Axonal injury, among other events, has been known to trigger rapid structural alterations,^11^ including changes in RGC dendritic arbors.^12^ The latter has been deduced to occur prior to manifest axonal loss, which led to synaptic rearrangements and functional deficits.^12^ RGC dendritic degeneration is one of the critical steps in glaucomatous optic neuropathy as shown in several models of acute and chronic ON damage. The integrity of other cells (such as astrocytes) in the ON is also important for visual function.^13^–^15^

Lipids constitute the outer boundary of mammalian cells and play important roles in structural integrity of RGC membranes, synapses, and dendrites.^15^ Genetic and genome-wide association studies have suggested impaired lipid metabolism in glaucoma.^16^,^17^ Phospholipids, sphingolipids, and ceramides are rich in the central nervous system (CNS) tissue^18^ as well as in membranes of generic neurons.^19^ These lipids have also been shown to undergo significant changes during axonal degeneration as well as in glaucoma.^20^–^22^ However, whereas the anterior segment of glaucomatous human eyes has been subjected to extensive lipidomic analysis,^23^–^27^ the ON has not been subjected to similar analysis. The studies presented here aim to fulfil this gap. In the present pilot studies, we aimed to determine which lipid classes demonstrate significant differences between control and glaucomatous ONs and whether existing underlying enzymatic differences can be readily demonstrated that support observed significant differences in class-specific lipid profiles. We focus here on those lipid classes that are enriched in the CNS and have been implicated in neurodegenerative disorders.

## Materials and Methods

### Tissue Procurement

Donor tissues were utilized for lipidomics following University of Miami Institutional Review Board approved protocols and in adherence with the tenets of the Declaration of Helsinki. Cadaveric donor eyes were procured from the Midwest Eye Bank (Cincinnati, OH, USA) and Lions Eye Bank (Miami, FL, USA). Donors were all Caucasian (inclusive of male and female) with mean age ± standard deviation of 72.3 ± 5.9 and 70.3 ± 10.5 for control and glaucoma donors, respectively (Supplementary Table S1; Supplemental Donor Information). Acceptable donor eyes were those with a postmortem to enucleation time of no more than 21 hours. Donor eyes were placed in a moist chamber with phosphate-buffered saline (PBS) at 4°C and shipped immediately. Eyes were carefully dissected usually within 36 hours post enucleation, and the ONs were explanted and stored at −80°C until analysis. In our previously reported studies, we evaluated and discussed the storage parameter (longer periods due to unavoidable reasons) effects for several ocular tissues.^23^ Similar to other ocular tissues, ON lipid profiles largely remain reproducible in moist chambers up to approximately 5 days. Acceptable glaucomatous eyes were those that had detailed medical and ophthalmic histories with a clear diagnosis of POAG. For this purpose, glaucoma donors should have had at least two static perimetry recordings within at least a ∼1- to 2-year interval showing loss of visual function (Supplemental Donor Information). In addition, many donor eyes had measurement of nerve fiber layer thickness and other clinical parameters recorded at two time points within at least a ∼1- to 2-year interval. Since the eyes were received first and clinical information followed, we performed mass spectrometric analysis of all eyes received. However, we used for further assessment only those eyes that had recordings of the information mentioned above. Thus, we could use only 12 eyes from a total 40 glaucoma donors and 11 eyes from a total 27 control donors for the analysis presented here. Furthermore, control eyes were from donors with no medical history of optic neuropathy. Additional donor tissues were used for Western blot, ELISA, and immunohistochemistry (Supplementary Table S1) in order to present representative outcomes. The ON was excised from ONH to the extent that the stalk was received in the enucleated donor eyes. The fatty tissue was cleared and the samples were washed with sterile PBS prior to further analysis.

### Lipid and Protein Extraction

Isolated ON tissue was weighed, minced with scissors and a fine scalpel, and then subjected to homogenization with a handheld homogenizer. Liquid nitrogen was intermittently added to the homogenizer as needed to facilitate tissue disruption. The homogenized tissue was subjected to lipid extraction of the organic phase using a modified Bligh and Dyer method^28^ as described in our previous reports.^23^,^25^,^26^ Briefly, after the tissue was disrupted and homogenized, methanol (LC-MS grade) and chloroform (LC-MS grade) were added in a 2:1 ratio to each sample. After 2 minutes of vigorous vortexing and 2 minutes of sonication in an ultrasonic bath, 3 mL ultrapure water and 1.5 mL chloroform were added, and samples were vigorously vortexed for 2 minutes and centrifuged at 10,062*g* at 4°C for 15 minutes to obtain phase separation. The organic phase from each was collected and dried under argon to prevent oxidation in a centrifugal vacuum concentrator. We then precipitated proteins from the aqueous phase (plus the interphase-precipitated proteins) by the addition of several volumes of methanol (referred to as corresponding aqueous phase). The corresponding aqueous phase–derived proteins were subjected to protein quantification using Bradford's method.^29^ The aqueous phase proteins were also fractionated on a PHAST gel (GE Healthcare Bio-Sciences AB, Uppsala, Sweden) and subjected to densitometric quantification using bovine serum albumin (BSA) as a standard.^30^ The aqueous phase proteins obtained from lipid extractions were found to result in suboptimal-quality Western blots. Lipid samples from each individual donor were stored at −20°C until reconstituted in 50 μL chloroform: methanol (1:1) prior to mass spectrometric analysis.

Protein extraction of ON samples was performed using buffer: 50 mM Tris-HCl 6.8 pH, 50 mM NaCl, and 1% SDS for Western blot analysis and ELISA. These extractions utilized separate donor samples (Supplementary Table S1). The minced ON tissues as mentioned above were homogenized for 5 minutes with a handheld homogenizer followed by recovery of supernatant using centrifugation at 100,62*g* for 5 minutes. Protein concentrations were also determined using Bradford's method^29^ and densitometrically on a PHAST gel system as described above.

### High-Performance Liquid Chromatography (HPLC)–Mass Spectrometry

Lipid samples were eluted using reverse phase liquid chromatography from an Acclaim C30 column (particle size 3.0 μm, 150 × 2.1 mm ID; Thermo Fisher Scientific, Waltham, MA, USA). The column was inserted in an HPLC Accela instrument (constituted by an autosampler and a 600 pump) was coupled to a Q-exactive mass spectrometer (Thermo Fisher Scientific) for high-resolution mass spectrometry. The column temperature was set to 30°C. The injection volume was 5 μL. A gradient of 20 minutes with a flow rate of 260 μL/min was run from 15% to 65% solvent B. Solvent A was a 60:40 ratio of methanol:water + 0.2% formic acid + 10 mM ammonium acetate. Solvent B was a 60:40 ratio of methanol:chloroform + 0.2% formic acid + 10 mM ammonium acetate. Heated electrospray ionization (HESI) was selected and used as the method of ionization by coupling a HESI probe to the Q-exactive instrument. The conditions set for the HESI probe were the following: Spray voltage was set to 4.4 Kv, heated capillary to 350°C, and heater to 275°C. The S-lens radio frequency (RF) was set to 70. The gas flow rate was set to 45 units and auxiliary gas to 15 units. The resolution for full scan was set to 70,000 at m/z 200, with an automatic gain control (AGC) target of 1x10E6 and a maximum injection time of 100 ms, and 17,500 for the data-dependent acquisition. The isolation window was set to 1.3 m/z, and the dynamic exclusion to 3 seconds. Collision energies were set to 30 and 19 eV in positive mode.

### Lipid Identification, Relative Quantification, and Processing

Raw files from mass spectrometry were analyzed for lipid identification using LipidSearch software v. 4.1, developed by Ryo Taguchi and Mitsui Knowledge Industry Co. (Tokyo, Japan). The following parameters were used for identification: parent and product search tolerance 5 ppm; product ion intensity; filters—top rank, main isomer peak, FA priority; quantification—m/z tolerance 5 ppm, retention time tolerance 0.5 minutes. The following adducts were allowed in positive mode: [M+H]^+^, [M+NH_4_]^+^, [M+H-H_2_O]^+^, [M+H-2H_2_O]^+^, M+2H]^2+^. All lipid classes were selected for search. All samples, control and glaucomatous were run in triplicates. All potential lipid matches for each sample triplicate were aligned on LipidSearch 4.1, and only the nonrejected were considered for inclusion and for further analysis. The retention time set for the alignment was 0.1 minute, top ranked filtered and main isomer peak selected. All peaks with the same annotated lipid species were merged in the result. M-score for alignments was set to 5.0, c-score to 2.0, and fatty acid chain identification to “A” and “B,” settings that allow the positive identification of the chains.

Data output from LipidSearch were grouped by lipid class [e.g., acyl carnitine (AcCa), bis-methyl lysophosphatidic acid (BisMelPA), cholesterol ester (ChE), diglyceride (DG)], fatty acid, and lipid species. Noninformative variables where characterized as values close to baseline or detection limit. These variables were filtered in MetaboAnalyst 4.0 (https://www.metaboanalyst.ca; provided in the public domain) and were detected using mean intensity value. Throughout the text, we have used LipidSearch Lipid nomenclature.

### Statistical Analyses

Analyses were conducted in MetaboAnalyst 4.0 and STATA 14.2. For principal component analysis (PCA), data were both quantile normalized and log_2_ transformed in order to remove skewness and nonbiological, procedure-based variation.^31^–^33^ For bar graphs, average peak intensity values are expressed as the mean ± SD. Unless stated otherwise, univariate analysis using false discovery rate (FDR)-adjusted Wilcoxon rank-sum and *t*-tests were performed to determine if there were differences in the peak area between control and glaucomatous lipid classes. Assessments of normal distribution were determined by Shapiro-Wilk's test (*P* > 0.05). Normality of variance was determined by Levene's test for equality of variance (*P >* 0.05). Post hoc power calculations were made in MetaboAnalyst, which estimates the average power for omics datasets with a large number of features and dimensionality. Preliminary analysis of the distribution of the *t*-statistic across the features revealed a normal distribution and there was a notable positive skew (left shift) in the distribution of the raw *P* values, indicating a strong effect. With an FDR set at 0.2 and the maximum sample per group at 60, a sample size of 10 yielded a power of 0.68. After discovering a significant difference in glucosylsphingosine peak intensity between the glaucoma and control ON, we sought to determine if other factors, namely, age and sex, were also associated with peak intensity for this class. A multiple linear regression was run with glucosylsphingosine peak intensity set as the dependent variable and with age, sex, and disease state (control/glaucoma) as covariates. Measures of collinearity were determined by variance inflation factor (VIF). A power analysis for the logistic regression with a H_0_: *R*^2^ = 0.0, H_a_: *R*^2^ = 0.3, alpha = 0.05, and *N* = 23 yielded a power of 0.64 with our sample size.

### Western Blot Analysis and ELISA Analysis

Extracted ON proteins were used for Western blot analyses. Equal amounts (20 μg) of proteins per sample were fractionated on a 4% to 15% sodium dodecyl-polyacrylamide gel electrophoresis (SDS-PAGE; Invitrogen, Carlsbad, CA, USA) and transferred onto a polyvinylidene fluoride (PVDF) membrane using precut blotting sandwiches (Ready Gel, catalog no. 162-0219; Bio-Rad, Hercules, CA, USA). Electrophoresis was performed using a constant 70 V for 90 minutes. After blotting, the PVDF membranes were blocked with 5% nonfat milk powder in a Tris-buffered saline (TBS) solution of pH 8. The primary antibodies against glucocerebrosidase (GBA) (rabbit, 1:5000, Abcam, Inc., Cambridge, UK), nonlysosomal glucocerebrosidase (GBA2) (rabbit, 1:500, Abcam, Inc.), acylsphingosine amidohydrolase (ASAH1) (rabbit, 1:5000, Abcam, Inc.), ASAH2 (rabbit, 1:5000, Abcam, Inc.), α-synuclein (sheep, 1:500, Abcam, Inc.), ceramide glucosyltransferase (UGCG) (rabbit polyclonal, 1:5000, Abcam, Inc.) and glyceraldehyde 3-phosphate dehydrogenase (GAPDH) (rabbit, 1:1000, Abcam, Inc.) were used for protein detection. For Western blot analysis and ELISA horseradish peroxidase (HRP) coupled (or AP-coupled for ELISA) secondary antibodies, goat anti-rabbit HRP (1:2000, Abcam, Inc.), goat anti-mouse HRP (1:2000, Abcam, Inc.), and donkey anti-sheep HRP (1:2000, Thermo Fisher Scientific) were used. Western blot was developed using chemiluminescence reagents on a GE ImageQuant LAS4000 (Marlborough, MA, USA).

ELISA was performed in triplicates using 5 μg extracted protein from control and glaucomatous ONs in 96-well plates following reported procedures.^34^ The absorbance was measured at 407 nm on a plate reader. An independent samples *t*-test was run to determine differences in the absorbance between control and glaucoma samples.

### Immunohistochemical Analyses

To determine localization, immunohistochemistry (IHC) was performed on paraffin-embedded ON sections (10 μm) using the same antibodies to GBA1 (1:200), GBA2 (1:200), ASAH1 (1:100), ASAH2 (1:100), and α-synuclein (1:500) mentioned previously. The sections were deparaffinized and washed with PBS, blocked with 0.2% BSA for 30 minutes, and subsequently incubated with primary antibodies overnight. Sections were then incubated for 1 hour at room temperature with Cy5-conjugated goat anti-rabbit (1:1000, Abcam, Inc.), Alexa 647 goat anti-rabbit (1:1000, Abcam, Inc.), or Alexa 594-conjugated donkey anti-sheep (1:1000, Abcam, Inc.). Control samples (without primary antibody–treated sections) were examined side by side on the same slide. Fluorescence images were obtained with a laser-scanning confocal imaging system (Zeiss LSM510; Peabody, MA, USA).

## Results

**Figure 1:**
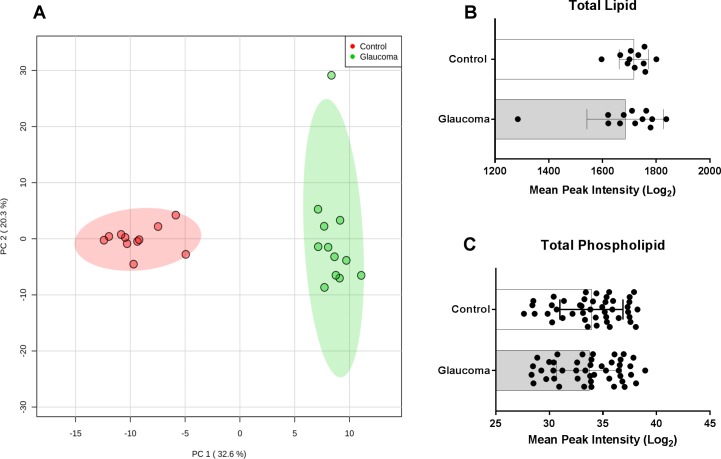
Principal component, total lipid, and total phospholipid composition of cadaveric human control and glaucomatous optic nerve (ON). (A) A two-dimensional principal component analysis (PCA) plot generated with MetaboAnalyst 4.0 using the peak intensity data. The control and glaucoma groups cluster into two completely separate categories. A two-component solution explained 52.9% of the total variance observed. One glaucoma ON sample (11G; Supplementary Table S1) was an outlier and categorized into its own cluster in k-means if a three-cluster solution was applied (analysis not shown). (B) Total lipid (overall mean peak intensity of all lipid classes) between control and glaucomatous ONs. Glaucomatous ONs were found to have a slightly lower (statistically insignificant, P = 0.85) peak intensity compared to control ONs. (C) Total phospholipid was found to be approximately the same between control and glaucomatous ON. Mean ± standard deviation are shown.

High-resolution mass spectrometric lipid profiling of a total of 23 human cadaveric eyes [11 control average age: 72.3 ± 5.9 years (6 males, 5 females), 12 glaucomatous average age: 70.3 ± 10.5 years (8 males, 4 females, Supplementary Table S1)] revealed two distinct clusters with respect to PCA (Fig. 1A). The scores for the first two principal components (PC1 and PC2) explained >50% of the variance, indicating that lipid classes in the ONs were highly different between the control and glaucomatous groups (Fig. 1A). There was not a significant difference between the death to eye preservation time for the control (10 ± 2.2 hours) and glaucomatous groups (11.6 ± 5.8 hours) (*P* = 0.4).

Total lipid content was slightly higher in control (peak area 1717 ± 54.8) compared to glaucoma ON (peak area 1685 ± 142.3) (Fig. 1B). A Wilcoxon rank-sum test was run and showed that differences in total lipid were not statistically significant (*P* = 0.9). Total phospholipids were subsequently analyzed. Analysis of phospholipids included the aggregate value of common phospholipid species (Supplementary Table S2), namely, phosphatidylcholine (PC), phosphatidylethanolamine (PE), phosphatidylserine (PS), and phosphatidylinositol (PI) categories. Phospholipids were also found to be similar in values between the control and glaucoma ON group (*P* = 0.73). Thus, phospholipids were not found to be significantly different between control and glaucomatous ONs (Fig. 1C; Supplementary Table S3).

**Figure 2:**
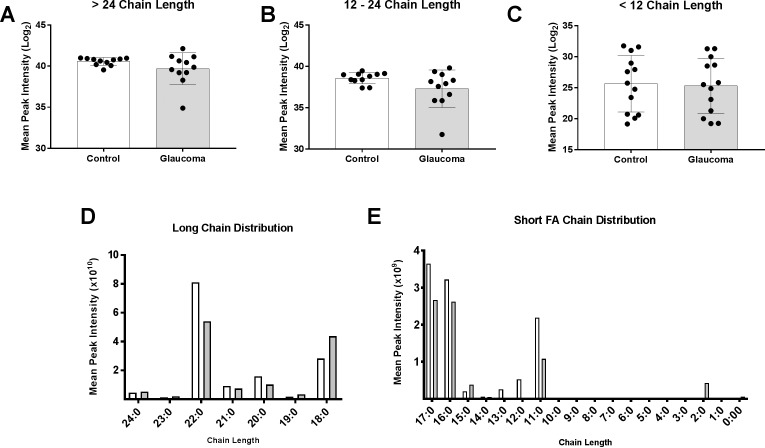
Fatty acyl chain length variation between control and glaucomatous human ON. The mean peak intensities of fatty acid chains—(A) acyl chain length greater than 24, (B) acyl chain length between 12 and 24, and (C) acyl chain length less than 12—between control and glaucoma group indicated that the glaucoma group had less overall fatty acids (statistically insignificant). (D) Distribution of long-chain fatty acids with chain lengths of 18 to 24 between control and glaucomatous ON. (E) The distribution of short-chain fatty acids (2:0–17:0) between control and glaucomatous ON. Hollow and gray bars in (D, E) depict control and glaucoma, respectively. Mean ± standard deviation are shown.

We next analyzed the fatty acids of varying chain length. Categorizing fatty acid chain into >24, 12 to 24, and <12, we found that control ONs had statistically insignificant greater peak intensities for the 12 to 24 (*P* = 0.087) and (*P* = 0.85) categories (Figs. 2A–C; Supplementary Table S4). To examine fatty acid chain distribution, we categorized fatty acid chains into chain lengths 18 to 24 (long chain) (Fig. 2D) and acyl chain length up to 17 (short chain) (Fig. 2E). In these two categories we found that the main drivers of these differences were fatty acids of acyl chain length 22:0 in the long-chain category and 11:0, 16:0, and 17:0 in the short-chain category (Figs. 2D, 2E).

**Figure 3:**
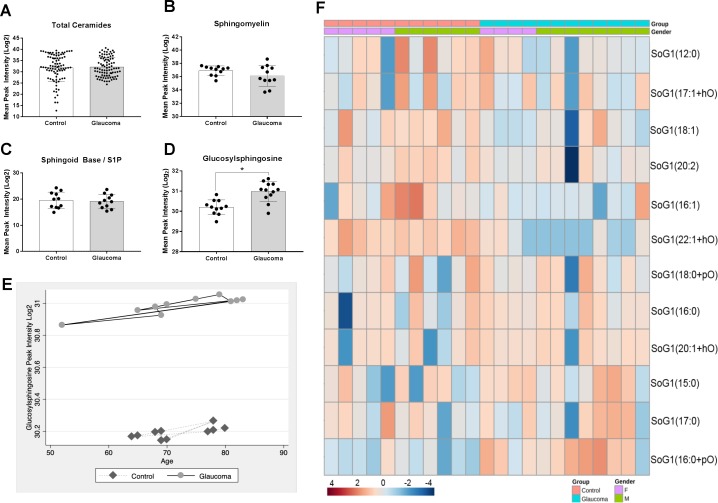
Total sphingolipid and ceramides and diversity of glucosylsphingosine lipid species between control and glaucomatous human ON. (A) The mean peak intensities of total ceramides of control and glaucoma indicated. The following species were included in total ceramide analysis: ceramides, ceramide phosphate, CerG1, CerG2, CerG3, CerG2GNAc1, CerG3GNAc1, and CerG3GNAc2. (B) Total sphingomyelins and (C) total sphingoid base and sphingosine-1-phosphate combined between control and glaucomatous ON. The differences were found to be statistically insignificant. (D) Total glucosylsphingosine species between control and glaucomatous ON (*P = 0.01). Mean ± standard deviation are shown. (E) Glucosylsphingosine peak intensity by age between control and glaucoma. (F) Heat map of 12 distinct species of glucosylsphingosine on a 4 to −4 scale as indicated. Data have been organized by group (control, glaucoma) and sex. Analysis parameters utilized the Ward clustering algorithm, Euclidean distance measure, and autoscaling based upon samples (performed using MetaboAnlayst 4.0).

We next assessed a series of related lipid classes enriched within the CNS, specifically ceramides, total sphingolipids, sphingoid base/sphingosine-1-phosphate, and glucosylsphingosine. We found no overall difference in total ceramide levels between the two groups (*P* = 0.66; Fig. 3A). We also did not detect a significant difference in total sphingomyelin (*P* = 0.30), despite finding a decreased sphingomyelin amount in POAG compared to controls (Fig. 3B). We combined sphingoid base and sphingosine-1-phospate and found the total amount of these species to be lower in glaucoma compared to controls (see Fig. 3D). However, the difference was also not significant *P* = 0.75). We next analyzed glucosylsphingosine and found total glucosylsphingosine to be significantly greater in glaucomatous ONs compared to controls (*P* < 0.01). A volcano plot was then used to visualize the differences in lipid classes between control and glaucoma (Supplementary Fig. S1). The fold change threshold was set at 1.5 and the FDR-adjusted *P* value threshold was set at 0.05. Only glucosylsphingosine (SoG1) was identified based on these parameters and was found to be enriched in glaucomatous ON.

A multiple regression was run to adjust for potential covariates (age and sex) for SoG1 peak intensity between glaucomatous and control groups. The multiple regression model explained 47% of the variance in SoG1 peak intensity, *F*(3, 19) = 5.64, *P* < 0.01, adjusted *R*^2^ = 0.47. Of the three predictor variables (age, sex, and disease state), only disease state was statistically significant (*P* = 0.001). Regression coefficients and standard errors can be found in Supplementary Table S5. The mean VIF value was 1.24, with none of the covariates rising above 1.35, suggesting no problems with multicollinearity. Posteriori power analysis yielded a power of 0.64 with our sample size of 23. To examine the interaction of increasing age on SoG1 peak intensity between control and glaucomatous ON, we modeled peak intensity from the regression for control and glaucoma and overlaid data with age (Fig. 3E). We observed a slight positive correlation between age and SoG1 peak intensity for control and glaucoma. We have presented species level details by group (control/glaucoma) and sex in a heat map (Fig. 3F). The analysis across all species for the ceramides, sphingomyelins, sphingosines, and SoG1s has been detailed in Supplementary Table S6.

**Figure 4:**
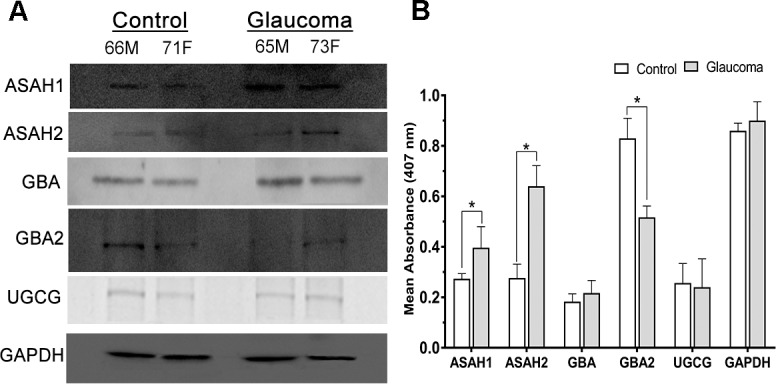
Western blot and ELISA of key enzymes involved in glucosylsphingosine metabolism. (A) Representative Western blot analysis from control and glaucomatous ON as indicated. Proteins from ON (20 μg) were run on 4% to 15% gradient SDS-PAGE and probed with antibodies to ASAH1, ASAH2, GBA, GBA2, and UGCG as shown. (B) Representative mean absorbance 407 nm from ELISA analysis with 1 μg protein per sample (n = 6 samples). Mean ± standard deviation from three independent experiments, *P ≤ 0.03. 66M, 65M = 66- and 65-year-old males; 71F, 73F = 71- and 73-year-old females as indicated.

Several enzymes are involved in critical conversion steps in the SoG1 metabolic pathway.^35^,^36^ Western blot analysis and ELISA were performed on control and glaucomatous ON protein extracts (Figs. 4A, 4B) to evaluate levels of lysosomal enzymes acylsphingosine deacylase 1 (ASAH1), glucosylceramidase beta (GBA), and nonlysosomal enzymes acylsphingosine deacylase 2 (ASAH2), glucosylceramidase beta 2 (GBA2), UDP-glucose UGCG that are critical for generation of SoG1. In GAPDH normalized Western blots, we found elevated ASAH1 and ASAH2 levels in glaucoma. We found similar levels of GBA and UGCG and lower GBA2 immunoreactivity in glaucoma compared to controls (Fig. 4A). These findings were consistent with the ELISA (Fig. 4B), showing a statistically significant increase in the absorbance of ASAH1 (*P* < 0.05) and ASAH2 (*P* < 0.01), and a decrease in GBA2 (*P* < 0.05) in glaucoma. Protein bands and mean absorbance from GBA and UGCG were approximately the same between control and glaucoma (Fig. 4B).

**Figure 5:**
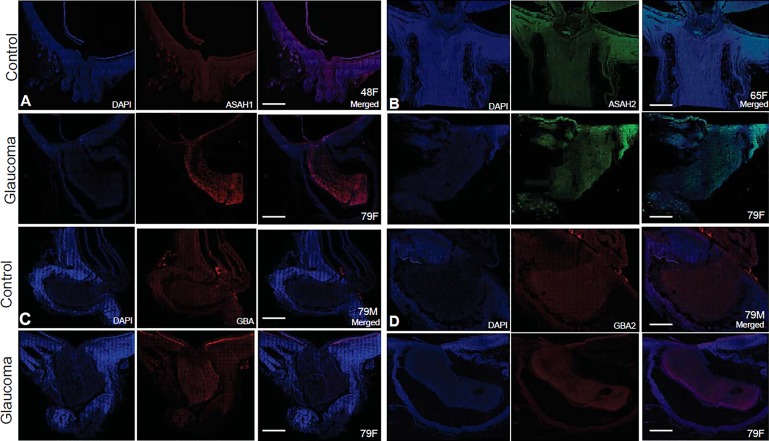
Representative immunohistochemistry of enzymes involved in glucosylsphingosine metabolism between control and glaucomatous human ON. ON sections (10 μm) were subjected to immunohistochemical analysis using primary and secondary antibodies as indicated. (A) Anti-ASAH1, probed with Alexa 647 goat anti-rabbit, (B) anti-ASAH2, Alexa 647 goat anti-rabbit, (C) anti-GBA, probed with Cy5-conjugated goat anti-rabbit, and (D) anti-GBA2, probed with Alexa 647 goat anti-rabbit. In each 4′,6-diamidino-2-phenylindole (DAPI) (blue) nucleus, stained image and merged image have been presented as indicated. Bar = 100 μm. Age and sex of (all Caucasian donors; Supplementary Table S1) are as indicated on each merged image.

We also examined the expression levels of alpha-synuclein, which appear to be comparable in control and POAG ONs (Supplementary Fig. S2). Immunohistochemical analyses showed localization of enzymes in the ONs (Figs. 5A–D). Identically treated control and glaucomatous ONs showed immunoreactivity of GBA, GBA2, ASAH1, and ASAH2 in the ONs of both groups. The overall fluorescence in the ON is consistent with Western blot and ELISA findings (Figs. 4A, 4B).

## Discussion

POAG and NTG both are characterized by a progressive loss of visual function. The primary difference between POAG and NTG is that the pathophysiological disease process progresses at a more normal level of IOP in NTG.^37^ The clinical presentation of the optic disc and visual function guides diagnosis of NTG in the absence of an elevated IOP. Interestingly, lowering the IOP remains a proven intervention strategy to slow down the progression of both pathologies.^37^ Thus, clinical outcomes may show a significant benefit due to alteration of a common and singular physical parameter: the IOP. It is conceivable that lowering the IOP would affect forces that result in excavation^9^,^10^ of the ON in glaucoma. Both forms of glaucoma are associated with remodeling of the ON resulting in progressive loss of visual function.

It is likely that disruption of cellular structure and connectivity occurs well before clinical presentations are manifested in glaucoma.^12^ The glaucomatous ON remodeling may occur even in the absence of elevated IOP, with latent molecular changes, warranting the examination of molecular mechanisms in glaucoma pathogenesis.

Lipids are important molecules that play a critical role in the maintenance of outer boundaries of cells and in dendrite stability.^12^,^15^ The genetic and genome-wide association studies (GWAS) have suggested impaired lipid pathways as possibilities to be associated with glaucoma pathology.^16^,^17^ To gain further insight into aberrations in lipids in glaucoma, we have performed high-resolution mass spectrometry on human ON extracted lipids. Whereas ONH is usually a nonmyelinated tissue, the ON is myelinated. Our analysis included both tissues. There is no good practical fractionation strategy to separate myelinated and nonmyelinated regions of ON yet for high throughput extractive mass spectrometric studies. Imaging mass spectrometry (IMS) is a great approach for localization of lipids in tissues, including the ON.^38^–^40^ However, IMS remains a poor method for comprehensive profiling due to several inherent limitations such as difficulties in volatilization, ionization, and nonuniformity in application of laser for ionization across the tissue. It is important to note that lipids demonstrate tremendous biodiversity surpassing that of metabolites.^41^ Currently it is estimated that there are approximately 10^5^ different lipid species grouped into 78 classes^42^,^43^ compared to approximately 10^4^ water-soluble metabolites in mammalian systems (ocular tissues included). Due to this tremendous diversity and complexity, the comprehensive biologically relevant results are based upon obtaining and performing analysis of lipids in selected classes. We focus here on phospholipid, sphingolipid, and ceramide lipid classes because of the enriched presence of these lipids in the membranes of (CNS) tissue^18^ and their potential implication in axonal degeneration,^20^,^21^ which is relevant for glaucoma and ON degeneration as well as for ON regeneration.

We report here our first analysis from human control and glaucomatous ON using extraction and high-resolution mass spectrometry. PCA analysis readily placed control and glaucomatous ON into two distinct groups based on lipid profiling (Fig. 1A). We found the total lipid amount to be similar in glaucoma and controls. Despite the mean total amount being slightly lower in glaucomatous ON, the difference for total lipids or total phospholipids amounts between control and glaucoma was statistically insignificant (Figs. 1B, 1C).

Several factors, such as diet, diabetes, alcoholism, smoking, and lack of exercise, to name a few, influence the total of amount of lipids in tissue. In ocular and CNS tissues the dietary partitioning is less significant. Determination of subtle changes in lipid metabolism in a neurodegenerative state thus necessitates assessment of lipid changes beyond total lipids, that is, lipid classes enriched within neuronal tissues, and investigation of specific lipid species. Two factors that can contribute to altered ON lipid profiles are cell death and remodeling of load-bearing tissues. Unless there is a significantly large asynchronous cell death with cells in different stages of decay, simple loss of cells is unlikely to significantly alter the gross lipid profile. As long as dead cells are cleared at a steady pace and their number at a given point of time is much smaller compared to total number of cells, the changes in lipid profile will not be significant. This is because that dead cells in the CNS immediately does not undergo a significant profile change and in proportion to total cells in tissue they are not significant contributors to lipid, especially phospholipid, amounts. The morphologic studies of cell death in glaucoma lack support for large asynchronous cell dealth.^44^–^46^ There is a possibility that remodeling of load-bearing tissues contributes to changes in lipid profiles. Experience from other fields and empirical estimates do not suggest that large changes compared to fraction in total lipids due to such changes render gross total lipid amount changes.^47^–^50^ Again, specific lipid species may provide insight into changes attributable to these factors.

To test whether our results indicate gross impairment of fatty acid metabolism, we analyzed acyl chains of varying lengths (Fig. 2). The relative amounts of fatty acids and the distribution of fatty acyl chain lengths showed statistically insignificant differences in glaucoma (Figs. 2A–C). However, we found a general decrease in acyl chain lengths 22 (Fig. 2D), 16, and 11 (Fig. 2E), respectively, in glaucoma, consistent with energy metabolism impairment.^51^,^52^

We next looked into total ceramides and sphingolipids [sphingomyelin, sphingoid base, sphingoid-1-phosphates (S1P)] and SoG1s (Figs. 3A–D). Sphingolipids and ceramides are often uniquely present in the membranes of the neurons where they likely play unique and important roles. Accumulation of these lipids in neuronal tissue has been known to cause defects in trafficking of proteins, impact calcium homeostasis, and activate apoptotic cascades that ultimately lead to cell death.^53^ Sphingomyelin, sphingoid base, and S1P were shown to have a statistically insignificant decrease in glaucomatous ON. The SoG1s, however, showed a significant increase in glaucomatous ON compared to control (Fig. 3D). A heat map of individual members of this group showed both an increase and decrease in specific SoG1 species in glaucomatous ON (Fig. 3E). It is evident that the species with increased levels outweigh any decrease by other members of the same group in glaucomatous ON (Fig. 3E, Supplementary Tables S5, S6).

The observed statistically significant increase in SoG1 (Figs. 3D, 3E) led us to investigate key lysosomal and nonlysosomal enzymes such as ASAH1, ASAH2, GBA, GBA2, UGCG, and GAPDH (Figs. 4A, 4B). Interestingly, we found increased levels of lysosomal ASAH1 but also nonlysosomal ASAH2 in the glaucomatous ON tissue. At the same time we found a decrease in lysosomal GBA2. Increased levels of ASAH1 and ASAH2 and decreased levels of GBA2 (Fig. 4) are consistent with the observed SoG1 levels (Figs. 3D, 3E). The levels of UGCG and GBA were similar with statistically insignificant differences between control and glaucomatous ON. The localization studies in the ON (Fig. 5) are consistent with the levels of enzymes found using other methods (Figs. 4A, 4B).

Elevated SoG1 is a biomarker for neurodegenerative Gaucher's disease,^54^,^55^ which is also characterized by simultaneous elevated levels of α-synuclein. To potentially distinguish Gaucher's from glaucomatous degeneration, we also assessed α-synuclein^56^,^57^ in the ON of control and glaucomatous donors (Supplementary Fig. S2). Consistent with previous reports,^58^,^59^ α-synuclein levels were similar in control and glaucomatous ON, suggesting that the observed elevated SoG1 levels (Figs. 3D, 3E) are unique for glaucomatous neurodegeneration, occurring in the absence of simultaneous elevated α-synuclein.

**Figure 6:**
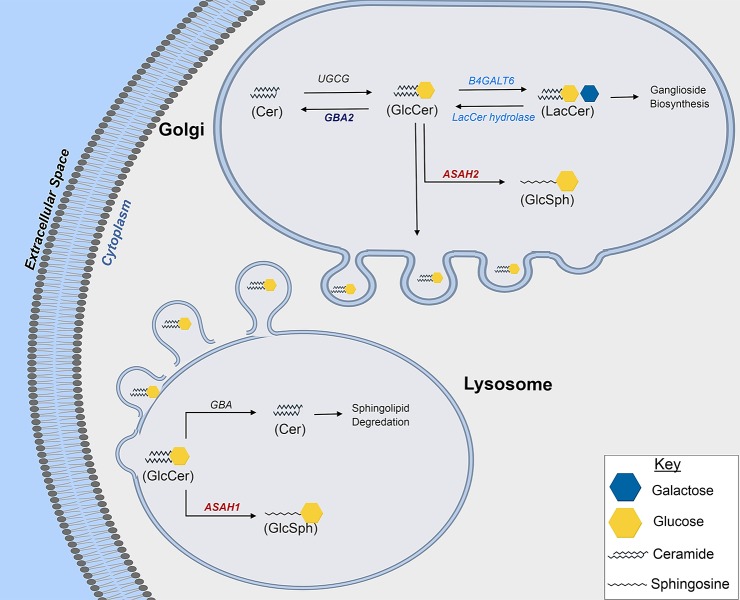
A schematic diagram summarizing the findings in the context of glucosylsphingosine metabolism pathway. The summary diagram depicts Golgi body, cytosol, and lysosome as indicated. In Golgi body the ceramide could be converted into GluCer by UGCG, which may then be converted into LacCer by LacCer synthase or into glucosylsphingosine (GluSph) by ASAH2 as indicated. GluCer is released by Golgi into the cytosol that reaches lysosomes. In lysosomes, ceramides can be generated from GluCer by GBA; alternatively, ASAH1 can convert the pool of GluCer into GluSph. Our findings on GluSph are consistent with elevated levels of ASAH1 and ASAH2. Observed reduced level of GBA2 will shift the equilibrium toward GluCer into glaucomatous eyes with elevated ASAH2 converting GluSph. Combining increased ASAH1 and ASAH2 and reduced GBA2 in glaucoma explains our observed increase in glucosylsphingosine in glaucomatous ON. Text highlighted in red and purple signifies upregulation and downregulation, respectively (determined by Western blot and ELISA analysis). Text in light blue indicates enzymes, whose levels have not been investigated due to the lack of observed differences in LacCer between control and glaucomatous ON.

Our results have been summarized in a schematic cartoon (Fig. 6). The observed decreased GBA2 and ASAH2 will convert glucosylceramide (GluCer) into SoG1. Importantly we did not determine LacCer synthase (B4GALT6) and LacCer hydrolase levels because no appreciable changes in LacCer were found in our analysis. Lysosomal glucosylceramidase beta (GBA) and nonlysosomal glucosylceramidase beta 2 (GBA2) are two enzymes that catalyze the conversion of GluCer to ceramide and free glucose. Decreases in glucosylceramidases have been associated with a large increase in both GluCer and SoG1.^35^ Lysosomal acylsphingosine deacylase 1 (ASAH1) and nonlysosomal acylsphingosine deacylase 2 (ASAH2) are responsible for direct intra-lysosomal and extra-lysosomal deacylation of GluCer to SoG1.^36^ UDP-glucose UGCG, a nonlysosomal Golgi resident enzyme, carries out catalysis of ceramide to GluCer. UGCG catalyzes the reverse reaction of GBA (Fig. 6). Our finding of elevated ASAH1 is consistent with increased conversion and accumulation of GlcSph in lysosomes. Importantly, lysosomal abnormalities in glaucoma have been noted both in the anterior^60^ and in the posterior ocular tissues, including the ON.^61^ Our findings presented here are a first step toward future investigations as to the precise region and cells within the ON contributing to increase in SoG1. Future investigations will pave the way to better understand the origin of the increased GlcSph and its precise role in the pathology of glaucoma at the ON.

In conclusion, we found a significant increase in SoG1 in glaucomatous ON compared to control, suggesting impaired biosynthesis and/or degradation in pathways leading to formation or accumulation of SoG1. We found decreased GBA2, as well as increased ASAH2 and ASAH1 levels in glaucomatous ON tissue, respectively. These enzymes are ordinarily resident in the Golgi body and lysosome compartments, suggesting that the lipid conversion pathways in these organelles are affected in glaucomatous ONs. Future investigations will enable mapping of tissue regions and cell types that precisely contribute to the observed increase in GlcSph and the levels of enzymes found in our current research work.

## Supplementary Material

Supplement 1
