# Supplementary material for: Optic Nerve Lipidomics Reveal Impaired Glucosylsphingosine Lipids Pathway in Glaucoma
Source: Invest Ophthalmol Vis Sci. 2019 Apr;60(5):1789–98. doi: 10.1167/iovs.18-25802 (PMC6485987; doi:10.1167/iovs.18-25802)
Supplement: Supplement 1 [file iovs-60-05-34_s01.pdf]

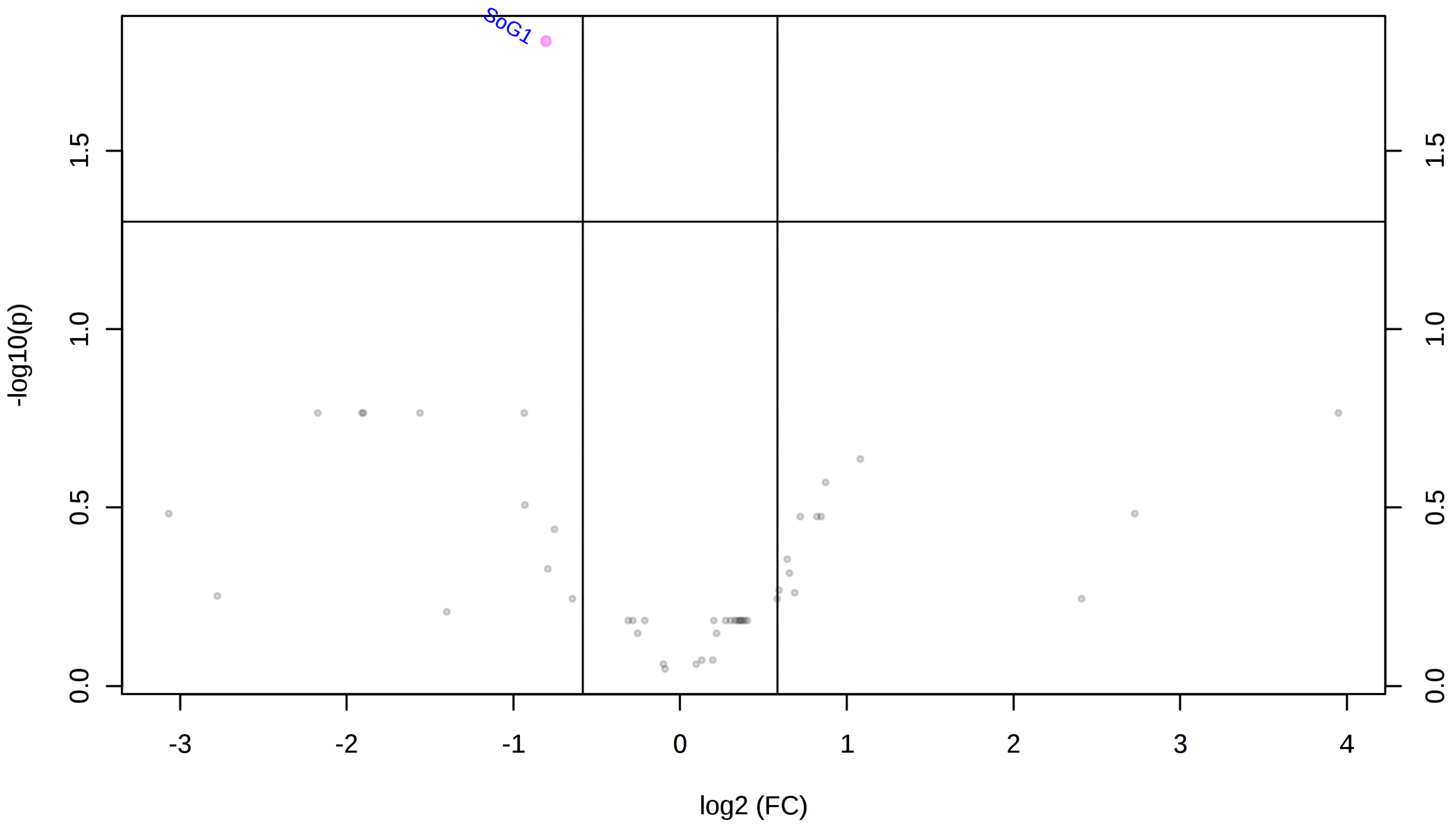

Supplemental Fig 1. Volcano plot of lipid classes identified in control and glaucomatous ON. When comparing lipid class profiles between two groups, a fold change (FC) threshold of 1.5 (x-axis) and an FDR-adjusted p-value (p) was set at 0.05 (y-axis). The fold changes have been log2 transformed and *p*-values have been -log10 transformed. Only one lipid class fell within those parameters and is highlighted in pink. SoG1 = glucosylsphingosine.

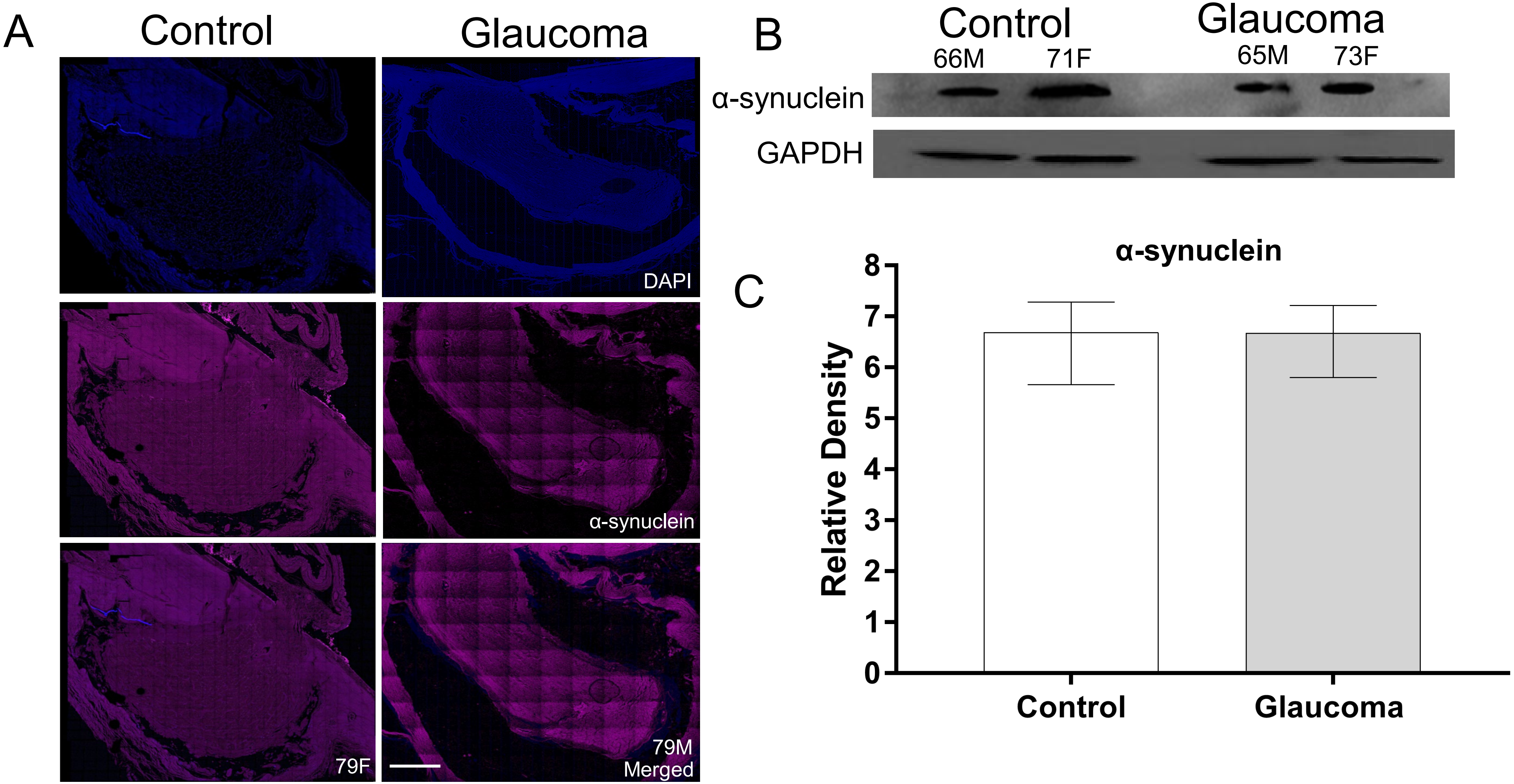

Supplemental Fig 2. Representative immunhistochemistry and Western blot analysis of α-synuclein protein. Control and glaucomatous donor optic nerves (ON) were subjected to immunohistochemical and Western blot analysis. (**A**) ON sections (10μm) were treated with Anti-α-synuclein and subsequently probed with Alexa 594-conjugated donkey anti-sheep secondary antibody. DAPI (blue) nuclei stained images and merged images have been presented as indicated. Bar = 100 μm. Age and gender are detailed on each merged image. (**B**) Proteins extracted from ON (20μg) were run on 4-15% gradient SDS-PAGE and probed with antibodies to α-synuclein. 66M and 65M indicate 66 and 65 year-old males; 71F and 73F indicate 71 and 73 year-old females, respectively. (C) Densitometric quantification of α-synuclein and GAPDH specific using ImageJ, α-synuclein immunoreactivity levels were normalized to GAPDH values. Mean values ± standard deviation are shown.

Supplemental Table 1: Optic Nerve donors‡

Donors used for Mass spectrometric lipidomics

Sample Number	Age	Gender	Time interval*	Cause of Death	MD	PSD (dB)	IOP (mmHg)	Max known IOP (mm Hg)
			Control †					
1N	78	M	8	Cardiac Arrest				
2N	78	M	9	Cardiac Arrest				
3N	69	F	11	Cardiac Arrest				
4N	69	M	12	Not known				
5N	65	M	14	COPD				
6N	64	M	10	COPD				
7N	77	F	12	Cardiac Arrest				
8N	78	F	9	Cardiac Arrest				
9N	80	F	10	Trauma				
10N	68	M	6	COPD				
11N	70	F	9	Cardiac arrest				
			Glaucoma					
1G	75	M	3	Cardiac arrest	-2.56 dB	2.54	14	22
2G	70	M	7	Subarachnoid hemorrhage	-4.93 dB	3.23	15	21
3G	68	M	6	Esophageal Cancer	-13.72 dB	9.52	17	27
4G	68	M	8	Lung Cancer	-6.44 dB	5.54	16	23
5G	69	F	19	Myocardial Infarction	-15.59 dB	9.54	19	30
6G	52	M	10	Not known	-3.96 dB	3.72	17	24
7G	82	F	21	COPD	-3.5 dB	2.52	14	22
8G	81	F	9	Ovarian Cancer	-6.9 dB	5.66	15	24
9G	79	M	19	Myocardial Infarction	-4.6 dB	3.96	16	22
10G	65	M	11	Not known	-2.2 dB	2.22	14	22
11G	83	F	10	Not known	-13.90 dB	9.55	17	26
12G	52	M	16	Myocardial Infarction	-6.55 dB	4.58	15	23

Western and ELISA analysis

Sample Number	Age	Gender	Time interval*	Cause of Death	MD	PSD (dB)	IOP (mmHg)	Max known IOP (mm Hg)
			Control					
12N	66	M	10	Not known				
13N	71	F	9	Cardiac Arrest				
1N	78	M	8	Cardiac Arrest				
2N	78	M	9	Cardiac Arrest				

3N	69	F	11	Cardiac Arrest				
4N	69	M	12	Not known				
			Glaucoma					
13G	65	M	8	Myocardial Infarction	-4.55 dB	3.33	16	23
14G	73	F	9	Cancer	-6.66 dB	5.52	16	25
1G	75	M	3	Cardiac arrest	-2.56 dB	2.54	14	22
2G	70	M	7	Subarachnoid hemorrhage	-4.93 dB	3.23	15	21
3G	68	M	6	Esophageal Cancer	-13.72 dB	9.52	17	27
4G	68	M	8	Lung Cancer	-6.44 dB	5.54	16	23
			**Representative Immunohistochemistry donors**					
			Control					
14N	48	F	21	Cardiac Arrest				
15N	65	F	10	Breast Cancer				
16N	79	M	9	Cardiac Arrest				
			Glaucoma					
15G	79	F	10	Cardiac Arrest	-9.66 dB	7.52	16	24

*Time interval refers time from death to preservation.‡Eyes were preserved in phosphate buffered saline upon enucleation, maintained at 2-8ºC and transported. The glaucoma donors were selected to avoid those who had hyperlimidemia and/or were on statins. Shaded area are donors who are common between lipidomics and biochemical experiments.

Supplemental Table 2: Common Phospholipid Species

Lipid Species✣	FA	Calc. Mass	Formula
PC(11:0/18:1)	11:0/18:1	689.4996	C37 H72 O8 N1 P1
PC(14:0p/18:1)	14:0p/18:1	715.5516	C40 H78 O7 N1 P1
PC(14:0p/23:1)	14:0p/23:1	785.6298	C45 H88 O7 N1 P1
PC(15:0/16:0)	15:0/16:0	719.5465	C39 H78 O8 N1 P1
PC(15:0/18:1)	15:0/18:1	745.5622	C41 H80 O8 N1 P1
PC(15:0/20:3)	15:0/20:3	769.5622	C43 H80 O8 N1 P1
PC(16:0/18:3)	16:0/18:3	755.5465	C42 H78 O8 N1 P1
PC(16:0/20:1)	16:0/20:1	787.6091	C44 H86 O8 N1 P1
PC(16:0/21:1)	16:0/21:1	801.6248	C45 H88 O8 N1 P1
PC(16:0/22:1)	16:0/22:1	815.6404	C46 H90 O8 N1 P1
PC(16:0/24:1)	16:0/24:1	843.6717	C48 H94 O8 N1 P1
PC(16:0p/14:0)	16:0p/14:0	689.5359	C38 H76 O7 N1 P1
PC(16:0p/16:0)	16:0p/16:0	717.5672	C40 H80 O7 N1 P1
PC(16:0p/18:1)	16:0p/18:1	743.5829	C42 H82 O7 N1 P1
PC(16:0p/18:2)	16:0p/18:2	741.5672	C42 H80 O7 N1 P1
PC(16:0p/20:4)	16:0p/20:4	765.5672	C44 H80 O7 N1 P1
PC(16:0p/22:4)	16:0p/22:4	793.5985	C44 H80 O7 N1 P2
PC(16:1)	16:01	507.2961	C44 H80 O7 N1 P3
PC(16:1/18:1)	16:1/18:1	757.5622	C42 H80 O8 N1 P1
PC(17:0/18:1)	17:0/18:1	773.5935	C43 H84 O8 N1 P1
PC(17:1/18:0)	17:1/18:0	773.5935	C43 H84 O8 N1 P1
PC(17:1/18:1)	17:1/18:1	771.5778	C43 H82 O8 N1 P1
PC(18:0/15:0)	18:0/15:0	747.5778	C41 H82 O8 N1 P1
PC(18:0p/16:0)	18:0p/16:0	745.5985	C42 H84 O7 N1 P1
PC(18:0p/18:1)	18:0p/18:1	771.6142	C44 H86 O7 N1 P1
PC(18:0p/20:1)	18:0p/20:1	799.6455	C46 H90 O7 N1 P1
PC(18:1/13:0)	18:1/13:0	717.5309	C39 H76 O8 N1 P1
PC(18:2p/18:0)	18:2p/18:0	769.5985	C44 H84 O7 N1 P1
PC(18:2p/18:1)	18:2p/18:1	767.5829	C44 H82 O7 N1 P1
PC(18:3/18:2)	18:3/18:2	779.5465	C44 H78 O8 N1 P1
PC(19:4e)	19:4e	529.3168	C27 H48 O7 N1 P1
PC(20:0e/22:4)	20:0e/22:4	851.6768	C50 H94 O7 N1 P1
PC(20:0p/16:0)	20:0p/16:0	773.6298	C44 H88 O7 N1 P1
PC(21:0p)	21:0p	563.3951	C29 H58 O7 N1 P1
PC(21:2e)	21:2e	561.3794	C29 H56 O7 N1 P1
PC(22:0/18:2)	22:0/18:2	841.6561	C48 H92 O8 N1 P1
PC(22:4/13:0)	22:4/13:0	767.5465	C43 H78 O8 N1 P1
PC(24:0/18:2)	24:0/18:2	869.6874	C50 H96 O8 N1 P1
PC(24:2/18:2)	24:2/18:2	865.6561	C50 H92 O8 N1 P1
PC(25:0e)	25:0e	621.4733	C33 H68 O7 N1 P1
PC(25:0p)	25:0p	619.4577	C33 H66 O7 N1 P1
PC(25:1/16:0)	25:1/16:0	857.6874	C49 H96 O8 N1 P1
PC(25:1/16:1)	25:1/16:1	855.6717	C49 H94 O8 N1 P1
PC(26:0/16:0)	26:0/16:0	873.7187	C50 H100 O8 N1 P1

PC(26:1/15:0)	26:1/15:0	857.6874	C49 H96 O8 N1 P1
PC(26:1/16:0)	26:1/16:0	871.703	C50 H98 O8 N1 P1
PC(26:1/17:1)	26:1/17:1	883.703	C51 H98 O8 N1 P1
PC(26:1/18:2)	26:1/18:2	895.703	C52 H98 O8 N1 P1
PC(26:1/19:1)	26:1/19:1	911.7343	C53 H102 O8 N1 P1
PC(27:0/16:0)	27:0/16:0	887.7343	C51 H102 O8 N1 P1
PC(27:0e)	27:0e	649.5046	C35 H72 O7 N1 P1
PC(27:0p)	27:0p	647.489	C35 H70 O7 N1 P1
PC(27:1/16:0)	27:1/16:0	885.7187	C51 H100 O8 N1 P1
PC(27:1/17:1)	27:1/17:1	897.7187	C52 H100 O8 N1 P1
PC(28:0)	28:00:00	677.4996	C36 H72 O8 N1 P1
PC(28:1/10:3)	28:1/10:3	809.5935	C46 H84 O8 N1 P1
PC(28:1/16:0)	28:1/16:0	899.7343	C52 H102 O8 N1 P1
PC(28:1/16:1)	28:1/16:1	897.7187	C52 H100 O8 N1 P1
PC(28:1/19:1)	28:1/19:1	939.7656	C55 H106 O8 N1 P1
PC(28:1/20:0)	28:1/20:0	955.7969	C56 H110 O8 N1 P1
PC(28:2e)	28:2e	659.489	C36 H70 O7 N1 P1
PC(28:2p)	28:2p	657.4733	C36 H68 O7 N1 P1
PC(29:0)	29:00:00	691.5152	C37 H74 O8 N1 P1
PC(29:0p)	29:0p	675.5203	C37 H74 O7 N1 P1
PC(29:1/16:0)	29:1/16:0	913.75	C53 H104 O8 N1 P1
PC(29:7)	29:07:00	677.4057	C37 H60 O8 N1 P1
PC(30:0)	30:00:00	705.5309	C38 H76 O8 N1 P1
PC(30:0e)	30:0e	691.5516	C38 H78 O7 N1 P1
PC(30:1)	30:01:00	703.5152	C38 H74 O8 N1 P1
PC(30:1/16:0)	30:1/16:0	927.7656	C54 H106 O8 N1 P1
PC(30:2e)	30:2e	687.5203	C38 H74 O7 N1 P1
PC(31:0e)	31:0e	705.5672	C39 H80 O7 N1 P1
PC(31:0p)	31:0p	703.5516	C39 H78 O7 N1 P1
PC(31:1)	31:01:00	717.5309	C39 H76 O8 N1 P1
PC(31:1/16:0)	31:1/16:0	941.7813	C55 H108 O8 N1 P1
PC(31:2)	31:02:00	715.5152	C39 H74 O8 N1 P1
PC(31:2p)	31:2p	699.5203	C39 H74 O7 N1 P1
PC(31:8)	31:08:00	703.4213	C39 H62 O8 N1 P1
PC(32:0)	32:00:00	733.5622	C40 H80 O8 N1 P1
PC(32:0e)	32:0e	719.5829	C40 H82 O7 N1 P1
PC(32:1)	32:01:00	731.5465	C40 H78 O8 N1 P1
PC(32:1/18:1)	32:1/18:1	981.8126	C58 H112 O8 N1 P1
PC(32:2)	32:02:00	729.5309	C40 H76 O8 N1 P1
PC(32:3)	32:03:00	727.5152	C40 H74 O8 N1 P1
PC(32:6p)	32:6p	705.4733	C40 H68 O7 N1 P1
PC(33:0)	33:00:00	747.5778	C41 H82 O8 N1 P1
PC(33:1)	33:01:00	745.5622	C41 H80 O8 N1 P1
PC(33:2)	33:02:00	743.5465	C41 H78 O8 N1 P1
PC(33:3)	33:03:00	741.5309	C41 H76 O8 N1 P1
PC(34:0)	34:00:00	761.5935	C42 H84 O8 N1 P1
PC(34:0/11:1)	34:0/11:1	913.75	C53 H104 O8 N1 P1
PC(34:0e)	34:0e	747.6142	C42 H86 O7 N1 P1

PC(34:1)	34:01:00	759.5778	C42 H82 O8 N1 P1
PC(34:1/12:3)	34:1/12:3	921.7187	C54 H100 O8 N1 P1
PC(34:1/12:4)	34:1/12:4	919.703	C54 H98 O8 N1 P1
PC(34:1/18:1)	34:1/18:1	1009.8439	C60 H116 O8 N1 P1
PC(34:2)	34:02:00	757.5622	C42 H80 O8 N1 P1
PC(34:3)	34:03:00	755.5465	C42 H78 O8 N1 P1
PC(34:4)	34:04:00	753.5309	C42 H76 O8 N1 P1
PC(35:0p)	35:0p	759.6142	C43 H86 O7 N1 P1
PC(35:1p)	35:1p	757.5985	C43 H84 O7 N1 P1
PC(35:2p)	35:2p	755.5829	C43 H82 O7 N1 P1
PC(35:3)	35:03:00	769.5622	C43 H80 O8 N1 P1
PC(35:4)	35:04:00	767.5465	C43 H78 O8 N1 P1
PC(35:5)	35:05:00	765.5309	C43 H76 O8 N1 P1
PC(36:0)	36:00:00	789.6248	C44 H88 O8 N1 P1
PC(36:1/11:1)	36:1/11:1	939.7656	C55 H106 O8 N1 P1
PC(36:1/12:3)	36:1/12:3	949.75	C56 H104 O8 N1 P1
PC(36:1/12:4)	36:1/12:4	947.7343	C56 H102 O8 N1 P1
PC(36:2)	36:02:00	785.5935	C44 H84 O8 N1 P1
PC(36:3)	36:03:00	783.5778	C44 H82 O8 N1 P1
PC(36:4e)	36:4e	767.5829	C44 H82 O7 N1 P1
PC(36:5p)	36:5p	763.5516	C44 H78 O7 N1 P1
PC(37:0p)	37:0p	787.6455	C45 H90 O7 N1 P1
PC(37:1)	37:01:00	801.6248	C45 H88 O8 N1 P1
PC(37:2)	37:02:00	799.6091	C45 H86 O8 N1 P1
PC(37:2p)	37:2p	783.6142	C45 H86 O7 N1 P1
PC(37:3)	37:03:00	797.5935	C45 H84 O8 N1 P1
PC(37:4)	37:04:00	795.5778	C45 H82 O8 N1 P1
PC(37:6)	37:06:00	791.5465	C45 H78 O8 N1 P1
PC(38:0)	38:00:00	817.6561	C46 H92 O8 N1 P1
PC(38:0e)	38:0e	803.6768	C46 H94 O7 N1 P1
PC(38:1e)	38:1e	801.6611	C46 H92 O7 N1 P1
PC(38:2)	38:02:00	813.6248	C46 H88 O8 N1 P1
PC(38:2e)	38:2e	799.6455	C46 H90 O7 N1 P1
PC(38:3)	38:03:00	811.6091	C46 H86 O8 N1 P1
PC(38:3e)	38:3e	797.6298	C46 H88 O7 N1 P1
PC(38:4e)	38:4e	795.6142	C46 H86 O7 N1 P1
PC(38:5)	38:05:00	807.5778	C46 H82 O8 N1 P1
PC(38:6e)	38:6e	791.5829	C46 H82 O7 N1 P1
PC(38:8)	38:08:00	801.5309	C46 H76 O8 N1 P1
PC(39:0p)	39:0p	815.6768	C47 H94 O7 N1 P1
PC(39:1)	39:01:00	829.6561	C47 H92 O8 N1 P1
PC(39:3)	39:03:00	825.6248	C47 H88 O8 N1 P1
PC(39:4)	39:04:00	823.6091	C47 H86 O8 N1 P1
PC(39:6)	39:06:00	819.5778	C47 H82 O8 N1 P1
PC(40:0)	40:00:00	845.6874	C48 H96 O8 N1 P1
PC(40:0e)	40:0e	831.7081	C48 H98 O7 N1 P1
PC(40:1e)	40:1e	829.6924	C48 H96 O7 N1 P1
PC(40:2e)	40:2e	827.6768	C48 H94 O7 N1 P1

PC(40:2p)	40:2p	825.6611	C48 H92 O7 N1 P1
PC(40:3)	40:03:00	839.6404	C48 H90 O8 N1 P1
PC(40:3p)	40:3p	823.6455	C48 H90 O7 N1 P1
PC(40:4)	40:04:00	837.6248	C48 H88 O8 N1 P1
PC(40:5)	40:05:00	835.6091	C48 H86 O8 N1 P1
PC(40:5e)	40:5e	821.6298	C48 H88 O7 N1 P1
PC(40:6)	40:06:00	833.5935	C48 H84 O8 N1 P1
PC(40:7)	40:07:00	831.5778	C48 H82 O8 N1 P1
PC(40:8)	40:08:00	829.5622	C48 H80 O8 N1 P1
PC(40:8p)	40:8p	813.5672	C48 H80 O7 N1 P1
PC(41:0)	41:00:00	859.703	C49 H98 O8 N1 P1
PC(41:1p)	41:1p	841.6924	C49 H96 O7 N1 P1
PC(41:2)	41:02:00	855.6717	C49 H94 O8 N1 P1
PC(42:0)	42:00:00	873.7187	C50 H100 O8 N1 P1
PC(42:0e)	42:0e	859.7394	C50 H102 O7 N1 P1
PC(42:2e)	42:2e	855.7081	C50 H98 O7 N1 P1
PC(42:2p)	42:2p	853.6924	C50 H96 O7 N1 P1
PC(42:3)	42:03:00	867.6717	C50 H94 O8 N1 P1
PC(42:4)	42:04:00	865.6561	C50 H92 O8 N1 P1
PC(42:4p)	42:4p	849.6611	C50 H92 O7 N1 P1
PC(42:5)	42:05:00	863.6404	C50 H90 O8 N1 P1
PC(42:6e)	42:6e	847.6455	C50 H90 O7 N1 P1
PC(43:3)	43:03:00	881.6874	C51 H96 O8 N1 P1
PC(44:0)	44:00:00	901.75	C52 H104 O8 N1 P1
PC(44:2e)	44:2e	883.7394	C52 H102 O7 N1 P1
PC(44:2p)	44:2p	881.7237	C52 H100 O7 N1 P1
PC(44:4)	44:04:00	893.6874	C52 H96 O8 N1 P1
PC(44:5)	44:05:00	891.6717	C52 H94 O8 N1 P1
PC(45:0)	45:00:00	915.7656	C53 H106 O8 N1 P1
PC(45:3)	45:03:00	909.7187	C53 H100 O8 N1 P1
PC(45:4)	45:04:00	907.703	C53 H98 O8 N1 P1
PC(45:5)	45:05:00	905.6874	C53 H96 O8 N1 P1
PC(46:0)	46:00:00	929.7813	C54 H108 O8 N1 P1
PC(46:2)	46:02:00	925.75	C54 H104 O8 N1 P1
PC(46:3)	46:03:00	923.7343	C54 H102 O8 N1 P1
PC(46:4)	46:04:00	921.7187	C54 H100 O8 N1 P1
PC(46:6)	46:06:00	917.6874	C54 H96 O8 N1 P1
PC(47:3)	47:03:00	937.75	C55 H104 O8 N1 P1
PC(47:4)	47:04:00	935.7343	C55 H102 O8 N1 P1
PC(47:5)	47:05:00	933.7187	C55 H100 O8 N1 P1
PC(47:6)	47:06:00	931.703	C55 H98 O8 N1 P1
PC(48:1)	48:01:00	955.7969	C56 H110 O8 N1 P1
PC(48:2)	48:02:00	953.7813	C56 H108 O8 N1 P1
PC(48:3)	48:03:00	951.7656	C56 H106 O8 N1 P1
PC(48:4)	48:04:00	949.75	C56 H104 O8 N1 P1
PC(48:6)	48:06:00	945.7187	C56 H100 O8 N1 P1
PC(49:2)	49:02:00	967.7969	C57 H110 O8 N1 P1
PC(49:5)	49:05:00	961.75	C57 H104 O8 N1 P1

PC(50:2)	50:02:00	981.8126	C58 H112 O8 N1 P1
PC(50:3)	50:03:00	979.7969	C58 H110 O8 N1 P1
PC(50:4)	50:04:00	977.7813	C58 H108 O8 N1 P1
PC(50:5)	50:05:00	975.7656	C58 H106 O8 N1 P1
PC(51:2)	51:02:00	995.8282	C59 H114 O8 N1 P1
PC(52:2)	52:02:00	1009.8439	C60 H116 O8 N1 P1
PC(52:3)	52:03:00	1007.8282	C60 H114 O8 N1 P1
PC(52:5)	52:05:00	1003.7969	C60 H110 O8 N1 P1
PC(52:6)	52:06:00	1001.7813	C60 H108 O8 N1 P1
PC(52:7)	52:07:00	999.7656	C60 H106 O8 N1 P1
PC(53:1)	53:01:00	1025.8752	C61 H120 O8 N1 P1
PC(54:2)	54:02:00	1037.8752	C62 H120 O8 N1 P1
PC(54:5)	54:05:00	1031.8282	C62 H114 O8 N1 P1
PC(54:6)	54:06:00	1029.8126	C62 H112 O8 N1 P1
PC(54:7)	54:07:00	1027.7969	C62 H110 O8 N1 P1
PC(56:5)	56:05:00	1059.8595	C64 H118 O8 N1 P1
PC(58:4)	58:04:00	1089.9065	C66 H124 O8 N1 P1
PC(58:5)	58:05:00	1087.8908	C66 H122 O8 N1 P1
PC(58:6)	58:06:00	1085.8752	C66 H120 O8 N1 P1
PC(60:6)	60:06:00	1113.9065	C68 H124 O8 N1 P1
PC(8:0e/18:1)	8:0e/18:1	633.4733	C34 H68 O7 N1 P1
PC(16:0/22:4)	16:0/22:4	809.5935	C46 H84 O8 N1 P1
PC(20:1p/16:0)	20:1p/16:0	771.6142	C44 H86 O7 N1 P1
PC(16:0/16:0)	16:0/16:0	733.5622	C40 H80 O8 N1 P1
PC(18:1/18:3)	18:1/18:3	781.5622	C44 H80 O8 N1 P1
PC(28:1/17:1)	28:1/17:1	911.7343	C53 H102 O8 N1 P1
PC(28:1/18:1)	28:1/18:1	925.75	C54 H104 O8 N1 P1
PC(30:1e)	30:1e	689.5359	C38 H76 O7 N1 P1
PC(31:0)	31:00:00	719.5465	C39 H78 O8 N1 P1
PC(32:1p)	32:1p	715.5516	C40 H78 O7 N1 P1
PC(34:2p)	34:2p	741.5672	C42 H80 O7 N1 P1
PC(36:1)	36:01:00	787.6091	C44 H86 O8 N1 P1
PC(36:1e)	36:1e	773.6298	C44 H88 O7 N1 P1
PC(36:4)	36:04:00	781.5622	C44 H80 O8 N1 P1
PC(36:4p)	36:4p	765.5672	C44 H80 O7 N1 P1
PC(36:5)	36:05:00	779.5465	C44 H78 O8 N1 P1
PC(38:1)	38:01:00	815.6404	C46 H90 O8 N1 P1
PC(38:4p)	38:4p	793.5985	C46 H84 O7 N1 P1
PC(38:6)	38:06:00	805.5622	C46 H80 O8 N1 P1
PC(40:1)	40:01:00	843.6717	C48 H94 O8 N1 P1
PC(40:2)	40:02:00	841.6561	C48 H92 O8 N1 P1
PC(40:7p)	40:7p	815.5829	C48 H82 O7 N1 P1
PC(42:2)	42:02:00	869.6874	C50 H96 O8 N1 P1
PC(44:3)	44:03:00	895.703	C52 H98 O8 N1 P1
PC(46:5)	46:05:00	919.703	C54 H98 O8 N1 P1
PC(48:5)	48:05:00	947.7343	C56 H102 O8 N1 P1
PC(50:6)	50:06:00	973.75	C58 H104 O8 N1 P1
PC(26:2e)	26:2e	631.4577	C34 H66 O7 N1 P1

PC(37:1p)	37:1p	785.6298	C45 H88 O7 N1 P1
PC(39:2p)	39:2p	811.6455	C47 H90 O7 N1 P1
PE(15:1)	15:01	451.2335	C20 H38 O8 N1 P1
PE(16:0/18:1)	16:0/18:1	717.5309	C39 H76 O8 N1 P1
PE(16:0/18:2)	16:0/18:2	715.5152	C39 H74 O8 N1 P1
PE(16:0/20:4)	16:0/20:4	739.5152	C41 H74 O8 N1 P1
PE(16:0/22:6)	16:0/22:6	763.5152	C43 H74 O8 N1 P1
PE(16:0p/16:0)	16:0p/16:0	675.5203	C37 H74 O7 N1 P1
PE(16:0p/18:1)	16:0p/18:1	701.5359	C39 H76 O7 N1 P1
PE(16:0p/19:1)	16:0p/19:1	715.5516	C40 H78 O7 N1 P1
PE(16:0p/20:1)	16:0p/20:1	729.5672	C41 H80 O7 N1 P1
PE(16:0p/20:3)	16:0p/20:3	725.5359	C41 H76 O7 N1 P1
PE(16:0p/20:4)	16:0p/20:4	723.5203	C41 H74 O7 N1 P1
PE(16:0p/22:3)	16:0p/22:3	753.5672	C43 H80 O7 N1 P1
PE(16:0p/22:4)	16:0p/22:4	751.5516	C43 H78 O7 N1 P1
PE(16:0p/22:6)	16:0p/22:6	747.5203	C43 H74 O7 N1 P1
PE(16:1/18:1)	16:1/18:1	715.5152	C39 H74 O8 N1 P1
PE(18:0/18:1)	18:0/18:1	745.5622	C41 H80 O8 N1 P1
PE(18:0/20:1)	18:0/20:1	773.5935	C43 H84 O8 N1 P1
PE(18:0/20:4)	18:0/20:4	767.5465	C43 H78 O8 N1 P1
PE(18:0/22:2)	18:0/22:2	799.6091	C45 H86 O8 N1 P1
PE(18:0/22:3)	18:0/22:3	797.5935	C45 H84 O8 N1 P1
PE(18:0/22:4)	18:0/22:4	795.5778	C45 H82 O8 N1 P1
PE(18:0/22:5)	18:0/22:5	793.5622	C45 H80 O8 N1 P1
PE(18:0/22:6)	18:0/22:6	791.5465	C45 H78 O8 N1 P1
PE(18:0e/20:1)	18:0e/20:1	759.6142	C43 H86 O7 N1 P1
PE(18:0p)	18:0p	479.3012	C23 H46 O7 N1 P1
PE(18:0p/19:1)	18:0p/19:1	743.5829	C42 H82 O7 N1 P1
PE(18:0p/20:1)	18:0p/20:1	757.5985	C43 H84 O7 N1 P1
PE(18:0p/21:1)	18:0p/21:1	771.6142	C44 H86 O7 N1 P1
PE(18:0p/22:1)	18:0p/22:1	785.6298	C45 H88 O7 N1 P1
PE(18:0p/22:3)	18:0p/22:3	781.5985	C45 H84 O7 N1 P1
PE(18:0p/22:4)	18:0p/22:4	779.5829	C45 H82 O7 N1 P1
PE(18:0p/22:6)	18:0p/22:6	775.5516	C45 H78 O7 N1 P1
PE(18:1/14:0)	18:1/14:0	689.4996	C37 H72 O8 N1 P1
PE(18:1/18:1)	18:1/18:1	743.5465	C41 H78 O8 N1 P1
PE(18:1/18:2)	18:1/18:2	741.5309	C41 H76 O8 N1 P1
PE(18:1/20:4)	18:1/20:4	765.5309	C43 H76 O8 N1 P1
PE(18:1p/14:0)	18:1p/14:0	673.5046	C37 H72 O7 N1 P1
PE(18:1p/16:1)	18:1p/16:1	699.5203	C39 H74 O7 N1 P1
PE(18:1p/18:1)	18:1p/18:1	727.5516	C41 H78 O7 N1 P1
PE(18:1p/20:1)	18:1p/20:1	755.5829	C43 H82 O7 N1 P1
PE(18:1p/20:4)	18:1p/20:4	749.5359	C43 H76 O7 N1 P1
PE(18:1p/21:1)	18:1p/21:1	769.5985	C44 H84 O7 N1 P1
PE(18:1p/22:1)	18:1p/22:1	783.6142	C45 H86 O7 N1 P1
PE(18:1p/22:4)	18:1p/22:4	777.5672	C45 H80 O7 N1 P1
PE(18:1p/22:6)	18:1p/22:6	773.5359	C45 H76 O7 N1 P1
PE(18:1p/23:1)	18:1p/23:1	797.6298	C46 H88 O7 N1 P1

PE(18:1p/24:1)	18:1p/24:1	811.6455	C47 H90 O7 N1 P1
PE(18:1p/24:2)	18:1p/24:2	809.6298	C47 H88 O7 N1 P1
PE(19:1)	19:01	507.2961	C24 H46 O8 N1 P1
PE(20:0p/22:4)	20:0p/22:4	807.6142	C47 H86 O7 N1 P1
PE(20:0p/22:5)	20:0p/22:5	805.5985	C47 H84 O7 N1 P1
PE(20:1/18:1)	20:1/18:1	771.5778	C43 H82 O8 N1 P1
PE(20:1/20:1)	20:1/20:1	799.6091	C45 H86 O8 N1 P1
PE(20:1/20:4)	20:1/20:4	793.5622	C45 H80 O8 N1 P1
PE(20:1p/22:4)	20:1p/22:4	805.5985	C47 H84 O7 N1 P1
PE(20:2e)	20:2e	505.3168	C25 H48 O7 N1 P1
PE(20:4e)	20:4e	501.2855	C25 H44 O7 N1 P1
PE(21:0p)	21:0p	521.3481	C26 H52 O7 N1 P1
PE(22:2)	22:02	547.3274	C27 H50 O8 N1 P1
PE(22:4e)	22:4e	529.3168	C27 H48 O7 N1 P1
PE(23:0e)	23:0e	551.3951	C28 H58 O7 N1 P1
PE(23:0p)	23:0p	549.3794	C28 H56 O7 N1 P1
PE(23:4)	23:04	557.3118	C28 H48 O8 N1 P1
PE(24:3e)	24:3e	559.3638	C29 H54 O7 N1 P1
PE(24:4e)	24:4e	557.3481	C29 H52 O7 N1 P1
PE(24:4p)	24:4p	555.3325	C29 H50 O7 N1 P1
PE(25:0e)	25:0e	579.4264	C30 H62 O7 N1 P1
PE(26:1/18:1)	26:1/18:1	855.6717	C49 H94 O8 N1 P1
PE(26:6)	26:06:00	595.3274	C31 H50 O8 N1 P1
PE(27:0e)	27:0e	607.4577	C32 H66 O7 N1 P1
PE(27:5)	27:05:00	611.3587	C32 H54 O8 N1 P1
PE(29:0e)	29:0e	635.489	C34 H70 O7 N1 P1
PE(29:1)	29:01:00	647.4526	C34 H66 O8 N1 P1
PE(29:1p)	29:1p	631.4577	C34 H66 O7 N1 P1
PE(30:9)	30:09:00	645.3431	C35 H52 O8 N1 P1
PE(32:4e)	32:4e	669.4733	C37 H68 O7 N1 P1
PE(32:5)	32:05:00	681.437	C37 H64 O8 N1 P1
PE(34:1)	34:01:00	717.5309	C39 H76 O8 N1 P1
PE(34:3p)	34:3p	697.5046	C39 H72 O7 N1 P1
PE(34:4p)	34:4p	695.489	C39 H70 O7 N1 P1
PE(34:6)	34:06:00	707.4526	C39 H66 O8 N1 P1
PE(34:6e)	34:6e	693.4733	C39 H68 O7 N1 P1
PE(34:8)	34:08:00	703.4213	C39 H62 O8 N1 P1
PE(35:3)	35:03:00	727.5152	C40 H74 O8 N1 P1
PE(36:4)	36:04:00	739.5152	C41 H74 O8 N1 P1
PE(36:4e)	36:4e	725.5359	C41 H76 O7 N1 P1
PE(36:6)	36:06:00	735.4839	C41 H70 O8 N1 P1
PE(37:0)	37:00:00	761.5935	C42 H84 O8 N1 P1
PE(37:1)	37:01:00	759.5778	C42 H82 O8 N1 P1
PE(37:2)	37:02:00	757.5622	C42 H80 O8 N1 P1
PE(37:3)	37:03:00	755.5465	C42 H78 O8 N1 P1
PE(37:3p)	37:3p	739.5516	C42 H78 O7 N1 P1
PE(37:4)	37:04:00	753.5309	C42 H76 O8 N1 P1
PE(38:4e)	38:4e	753.5672	C43 H80 O7 N1 P1

PE(38:4p)	38:4p	751.5516	C43 H78 O7 N1 P1
PE(38:7)	38:07:00	761.4996	C43 H72 O8 N1 P1
PE(39:6)	39:06:00	777.5309	C44 H76 O8 N1 P1
PE(40:1)	40:01:00	801.6248	C45 H88 O8 N1 P1
PE(40:3p)	40:3p	781.5985	C45 H84 O7 N1 P1
PE(40:5)	40:05:00	793.5622	C45 H80 O8 N1 P1
PE(40:5e)	40:5e	779.5829	C45 H82 O7 N1 P1
PE(40:6)	40:06:00	791.5465	C45 H78 O8 N1 P1
PE(40:6e)	40:6e	777.5672	C45 H80 O7 N1 P1
PE(41:5)	41:05:00	807.5778	C46 H82 O8 N1 P1
PE(42:2)	42:02:00	827.6404	C47 H90 O8 N1 P1
PE(42:3)	42:03:00	825.6248	C47 H88 O8 N1 P1
PE(42:3p)	42:3p	809.6298	C47 H88 O7 N1 P1
PE(42:4)	42:04:00	823.6091	C47 H86 O8 N1 P1
PE(42:6p)	42:6p	803.5829	C47 H82 O7 N1 P1
PE(42:8)	42:08:00	815.5465	C47 H78 O8 N1 P1
PE(43:0)	43:00:00	845.6874	C48 H96 O8 N1 P1
PE(44:2p)	44:2p	839.6768	C49 H94 O7 N1 P1
PE(44:3p)	44:3p	837.6611	C49 H92 O7 N1 P1
PE(44:6)	44:06:00	847.6091	C49 H86 O8 N1 P1
PE(44:7)	44:07:00	845.5935	C49 H84 O8 N1 P1
PE(46:2)	46:02:00	883.703	C51 H98 O8 N1 P1
PE(46:3)	46:03:00	881.6874	C51 H96 O8 N1 P1
PE(46:5)	46:05:00	877.6561	C51 H92 O8 N1 P1
PE(48:7)	48:07:00	901.6561	C53 H92 O8 N1 P1
PE(49:5)	49:05:00	919.703	C54 H98 O8 N1 P1
PE(50:1)	50:01:00	941.7813	C55 H108 O8 N1 P1
PE(50:5)	50:05:00	933.7187	C55 H100 O8 N1 P1
PE(50:7)	50:07:00	929.6874	C55 H96 O8 N1 P1
PE(52:7)	52:07:00	957.7187	C57 H100 O8 N1 P1
PE(53:2)	53:02:00	981.8126	C58 H112 O8 N1 P1
PE(54:5)	54:05:00	989.7813	C59 H108 O8 N1 P1
PE(56:5)	56:05:00	1017.8126	C61 H112 O8 N1 P1
PE(56:6)	56:06:00	1015.7969	C61 H110 O8 N1 P1
PE(8:0e/8:0)	8:0e/8:0	453.2855	C21 H44 O7 N1 P1
PE(18:0p/18:1)	18:0p/18:1	729.5672	C41 H80 O7 N1 P1
PE(18:0e)	18:0e	481.3168	C23 H48 O7 N1 P1
PE(38:5)	38:05:00	765.5309	C43 H76 O8 N1 P1
PE(18:0)	18:00	495.2961	C23 H46 O8 N1 P1
PE(32:6e)	32:6e	665.442	C37 H64 O7 N1 P1
PE(34:5)	34:05:00	709.4683	C39 H68 O8 N1 P1
PE(35:2p)	35:2p	713.5359	C40 H76 O7 N1 P1
PE(37:6)	37:06:00	749.4996	C42 H72 O8 N1 P1
PE(40:2p)	40:2p	783.6142	C45 H86 O7 N1 P1
PE(52:5)	52:05:00	961.75	C57 H104 O8 N1 P1
PE(53:6)	53:06:00	973.75	C58 H104 O8 N1 P1
PE(54:4)	54:04:00	991.7969	C59 H110 O8 N1 P1
PE(54:6)	54:06:00	987.7656	C59 H106 O8 N1 P1

PI(16:0/18:1)	16:0/18:1	836.5415	C43 H81 O13 N0 P1
PI(16:0/20:4)	16:0/20:4	858.5258	C45 H79 O13 N0 P1
PI(16:1)	16:01	584.2598	C25 H45 O13 N0 P1
PI(17:1)	17:01	598.2754	C26 H47 O13 N0 P1
PI(18:0/18:2)	18:0/18:2	862.5571	C45 H83 O13 N0 P1
PI(18:0/20:4)	18:0/20:4	886.5571	C47 H83 O13 N0 P1
PI(18:1/20:4)	18:1/20:4	884.5415	C47 H81 O13 N0 P1
PI(22:0)	22:00	670.3693	C31 H59 O13 N0 P1
PI(23:2)	23:02	680.3537	C32 H57 O13 N0 P1
PI(23:4e)	23:4e	662.3431	C32 H55 O12 N0 P1
PI(25:3)	25:03:00	706.3693	C34 H59 O13 N0 P1
PI(25:4)	25:04:00	704.3537	C34 H57 O13 N0 P1
PI(25:5)	25:05:00	702.338	C34 H55 O13 N0 P1
PI(26:2p)	26:2p	706.4057	C35 H63 O12 N0 P1
PI(26:3p)	26:3p	704.3901	C35 H61 O12 N0 P1
PI(27:0)	27:00:00	740.4476	C36 H69 O13 N0 P1
PI(27:3)	27:03:00	734.4006	C36 H63 O13 N0 P1
PI(27:4e)	27:4e	718.4057	C36 H63 O12 N0 P1
PI(29:4p)	29:4p	744.4214	C38 H65 O12 N0 P1
PI(30:7)	30:07:00	768.385	C39 H61 O13 N0 P1
PI(31:1p)	31:1p	778.4996	C40 H75 O12 N0 P1
PI(31:3)	31:03:00	790.4632	C40 H71 O13 N0 P1
PI(32:0)	32:00:00	810.5258	C41 H79 O13 N0 P1
PI(32:0e)	32:0e	796.5466	C41 H81 O12 N0 P1
PI(32:1p)	32:1p	792.5153	C41 H77 O12 N0 P1
PI(32:3)	32:03:00	804.4789	C41 H73 O13 N0 P1
PI(32:4)	32:04:00	802.4632	C41 H71 O13 N0 P1
PI(32:8)	32:08:00	794.4006	C41 H63 O13 N0 P1
PI(33:1)	33:01:00	822.5258	C42 H79 O13 N0 P1
PI(33:2)	33:02:00	820.5102	C42 H77 O13 N0 P1
PI(33:8)	33:08:00	808.4163	C42 H65 O13 N0 P1
PI(34:2)	34:02:00	834.5258	C43 H79 O13 N0 P1
PI(34:2e)	34:2e	820.5466	C43 H81 O12 N0 P1
PI(34:3)	34:03:00	832.5102	C43 H77 O13 N0 P1
PI(34:3p)	34:3p	816.5153	C43 H77 O12 N0 P1
PI(34:6p)	34:6p	810.4683	C43 H71 O12 N0 P1
PI(34:8)	34:08:00	822.4319	C43 H67 O13 N0 P1
PI(35:6)	35:06:00	840.4789	C44 H73 O13 N0 P1
PI(36:4e)	36:4e	844.5466	C45 H81 O12 N0 P1
PI(36:5)	36:05:00	856.5102	C45 H77 O13 N0 P1
PI(36:5p)	36:5p	840.5153	C45 H77 O12 N0 P1
PI(38:0)	38:00:00	894.6197	C47 H91 O13 N0 P1
PI(38:5)	38:05:00	884.5415	C47 H81 O13 N0 P1
PI(38:8)	38:08:00	878.4945	C47 H75 O13 N0 P1
PI(38:9)	38:09:00	876.4789	C47 H73 O13 N0 P1
PI(39:1p)	39:1p	890.6248	C48 H91 O12 N0 P1
PI(39:5)	39:05:00	898.5571	C48 H83 O13 N0 P1
PI(40:1e)	40:1e	906.6561	C49 H95 O12 N0 P1

PI(40:5e)	40:5e	898.5935	C49 H87 O12 N0 P1
PI(40:7)	40:07:00	908.5415	C49 H81 O13 N0 P1
PI(40:7p)	40:7p	892.5466	C49 H81 O12 N0 P1
PI(40:8)	40:08:00	906.5258	C49 H79 O13 N0 P1
PI(42:1)	42:01:00	948.6667	C51 H97 O13 N0 P1
PI(42:10)	42:10:00	930.5258	C51 H79 O13 N0 P1
PI(42:4)	42:04:00	942.6197	C51 H91 O13 N0 P1
PI(43:0)	43:00:00	964.698	C52 H101 O13 N0 P1
PI(43:1p)	43:1p	946.6874	C52 H99 O12 N0 P1
PI(43:6)	43:06:00	952.6041	C52 H89 O13 N0 P1
PI(44:5)	44:05:00	968.6354	C53 H93 O13 N0 P1
PI(45:0)	45:00:00	992.7293	C54 H105 O13 N0 P1
PI(47:5)	47:05:00	1010.6823	C56 H99 O13 N0 P1
PI(50:3)	50:03:00	1056.7606	C59 H109 O13 N0 P1
PI(50:7)	50:07:00	1048.698	C59 H101 O13 N0 P1
PI(52:4)	52:04:00	1082.7762	C61 H111 O13 N0 P1
PI(54:1)	54:01:00	1116.8545	C63 H121 O13 N0 P1
PI(56:7)	56:07:00	1132.7919	C65 H113 O13 N0 P1
PI(59:0)	59:00:00	1188.9484	C68 H133 O13 N0 P1
PI(18:0/20:3)	18:0/20:3	888.5728	C47 H85 O13 N0 P1
PI(15:2)	15:02	568.2285	C24 H41 O13 N0 P1
PI(25:0)	25:00:00	712.4163	C34 H65 O13 N0 P1
PI(27:0p)	27:0p	724.4527	C36 H69 O12 N0 P1
PI(28:2)	28:02:00	750.4319	C37 H67 O13 N0 P1
PI(28:5p)	28:5p	728.3901	C37 H61 O12 N0 P1
PI(31:1)	31:01:00	794.4945	C40 H75 O13 N0 P1
PI(32:2p)	32:2p	790.4996	C41 H75 O12 N0 P1
PI(33:0)	33:00:00	824.5415	C42 H81 O13 N0 P1
PI(34:1)	34:01:00	836.5415	C43 H81 O13 N0 P1
PI(36:1e)	36:1e	850.5935	C45 H87 O12 N0 P1
PI(36:2e)	36:2e	848.5779	C45 H85 O12 N0 P1
PI(37:3)	37:03:00	874.5571	C46 H83 O13 N0 P1
PI(38:1)	38:01:00	892.6041	C47 H89 O13 N0 P1
PI(40:9)	40:09:00	904.5102	C49 H77 O13 N0 P1
PI(55:1)	55:01:00	1130.8701	C64 H123 O13 N0 P1
PS(16:0/18:1)	16:0/18:1	761.5207	C40 H76 O10 N1 P1
PS(16:0p/18:1)	16:0p/18:1	745.5258	C40 H76 O9 N1 P1
PS(18:0/18:1)	18:0/18:1	789.552	C42 H80 O10 N1 P1
PS(18:0/20:1)	18:0/20:1	817.5833	C44 H84 O10 N1 P1
PS(18:0/20:3)	18:0/20:3	813.552	C44 H80 O10 N1 P1
PS(18:0/20:4)	18:0/20:4	811.5363	C44 H78 O10 N1 P1
PS(18:0/22:3)	18:0/22:3	841.5833	C46 H84 O10 N1 P1
PS(18:0/22:4)	18:0/22:4	839.5676	C46 H82 O10 N1 P1
PS(18:0/22:6)	18:0/22:6	835.5363	C46 H78 O10 N1 P1
PS(18:1/18:1)	18:1/18:1	787.5363	C42 H78 O10 N1 P1
PS(18:1/20:4)	18:1/20:4	809.5207	C44 H76 O10 N1 P1
PS(18:1/22:0)	18:1/22:0	845.6146	C46 H88 O10 N1 P1
PS(18:1/22:1)	18:1/22:1	843.5989	C46 H86 O10 N1 P1

PS(18:1/23:0)	18:1/23:0	859.6302	C47 H90 O10 N1 P1
PS(18:1/24:0)	18:1/24:0	873.6459	C48 H92 O10 N1 P1
PS(18:1/24:1)	18:1/24:1	871.6302	C48 H90 O10 N1 P1
PS(20:1/18:1)	20:1/18:1	815.5676	C44 H82 O10 N1 P1
PS(21:3)	21:03	575.2859	C27 H46 O10 N1 P1
PS(22:1)	22:01	593.3329	C28 H52 O10 N1 P1
PS(23:0)	23:00	609.3642	C29 H56 O10 N1 P1
PS(23:1)	23:01	607.3485	C29 H54 O10 N1 P1
PS(23:3)	23:03	603.3172	C29 H50 O10 N1 P1
PS(24:1/18:2)	24:1/18:2	869.6146	C48 H88 O10 N1 P1
PS(24:2/18:2)	24:2/18:2	867.5989	C48 H86 O10 N1 P1
PS(24:4e)	24:4e	601.338	C30 H52 O9 N1 P1
PS(26:1/18:1)	26:1/18:1	899.6615	C50 H94 O10 N1 P1
PS(27:0)	27:00:00	665.4268	C33 H64 O10 N1 P1
PS(28:0)	28:00:00	679.4424	C34 H66 O10 N1 P1
PS(28:0p)	28:0p	663.4475	C34 H66 O9 N1 P1
PS(28:1)	28:01:00	677.4268	C34 H64 O10 N1 P1
PS(28:1e)	28:1e	663.4475	C34 H66 O9 N1 P1
PS(29:0)	29:00:00	693.4581	C35 H68 O10 N1 P1
PS(29:3)	29:03:00	687.4111	C35 H62 O10 N1 P1
PS(29:5p)	29:5p	667.3849	C35 H58 O9 N1 P1
PS(30:0)	30:00:00	707.4737	C36 H70 O10 N1 P1
PS(30:4)	30:04:00	699.4111	C36 H62 O10 N1 P1
PS(30:4p)	30:4p	683.4162	C36 H62 O9 N1 P1
PS(31:0)	31:00:00	721.4894	C37 H72 O10 N1 P1
PS(31:4e)	31:4e	699.4475	C37 H66 O9 N1 P1
PS(31:5p)	31:5p	695.4162	C37 H62 O9 N1 P1
PS(31:9)	31:09:00	703.3485	C37 H54 O10 N1 P1
PS(32:0)	32:00:00	735.505	C38 H74 O10 N1 P1
PS(32:1)	32:01:00	733.4894	C38 H72 O10 N1 P1
PS(32:2)	32:02:00	731.4737	C38 H70 O10 N1 P1
PS(32:4e)	32:4e	713.4632	C38 H68 O9 N1 P1
PS(32:4p)	32:4p	711.4475	C38 H66 O9 N1 P1
PS(33:10)	33:10:00	729.3642	C39 H56 O10 N1 P1
PS(33:7)	33:07:00	735.4111	C39 H62 O10 N1 P1
PS(34:1)	34:01:00	761.5207	C40 H76 O10 N1 P1
PS(34:2)	34:02:00	759.505	C40 H74 O10 N1 P1
PS(34:6e)	34:6e	737.4632	C40 H68 O9 N1 P1
PS(34:6p)	34:6p	735.4475	C40 H66 O9 N1 P1
PS(36:0)	36:00:00	791.5676	C42 H82 O10 N1 P1
PS(36:1)	36:01:00	789.552	C42 H80 O10 N1 P1
PS(36:1e)	36:1e	775.5727	C42 H82 O9 N1 P1
PS(36:2p)	36:2p	771.5414	C42 H78 O9 N1 P1
PS(37:0)	37:00:00	805.5833	C43 H84 O10 N1 P1
PS(37:1)	37:01:00	803.5676	C43 H82 O10 N1 P1
PS(37:2)	37:02:00	801.552	C43 H80 O10 N1 P1
PS(37:5)	37:05:00	795.505	C43 H74 O10 N1 P1
PS(38:2e)	38:2e	801.5884	C44 H84 O9 N1 P1

PS(38:7p)	38:7p	789.4945	C44 H72 O9 N1 P1
PS(38:8)	38:08:00	803.4737	C44 H70 O10 N1 P1
PS(39:1)	39:01:00	831.5989	C45 H86 O10 N1 P1
PS(39:4)	39:04:00	825.552	C45 H80 O10 N1 P1
PS(39:6)	39:06:00	821.5207	C45 H76 O10 N1 P1
PS(39:7)	39:07:00	819.505	C45 H74 O10 N1 P1
PS(40:10)	40:10:00	827.4737	C46 H70 O10 N1 P1
PS(40:3p)	40:3p	825.5884	C46 H84 O9 N1 P1
PS(41:3p)	41:3p	839.604	C47 H86 O9 N1 P1
PS(42:3)	42:03:00	869.6146	C48 H88 O10 N1 P1
PS(42:4)	42:04:00	867.5989	C48 H86 O10 N1 P1
PS(43:4)	43:04:00	881.6146	C49 H88 O10 N1 P1
PS(44:0)	44:00:00	903.6928	C50 H98 O10 N1 P1
PS(44:4)	44:04:00	895.6302	C50 H90 O10 N1 P1
PS(45:0)	45:00:00	917.7085	C51 H100 O10 N1 P1
PS(45:6)	45:06:00	905.6146	C51 H88 O10 N1 P1
PS(47:7)	47:07:00	931.6302	C53 H90 O10 N1 P1
PS(48:7)	48:07:00	945.6459	C54 H92 O10 N1 P1
PS(49:0)	49:00:00	973.7711	C55 H108 O10 N1 P1
PS(21:0p)	21:0p	565.338	C27 H52 O9 N1 P1
PS(23:0p)	23:0p	593.3693	C29 H56 O9 N1 P1
PS(26:2)	26:02:00	647.3798	C32 H58 O10 N1 P1
PS(28:2)	28:02:00	675.4111	C34 H62 O10 N1 P1
PS(31:4p)	31:4p	697.4319	C37 H64 O9 N1 P1
PS(32:1p)	32:1p	717.4945	C38 H72 O9 N1 P1
PS(34:2e)	34:2e	745.5258	C40 H76 O9 N1 P1
PS(35:0)	35:00:00	777.552	C41 H80 O10 N1 P1
PS(35:1p)	35:1p	759.5414	C41 H78 O9 N1 P1
PS(37:1p)	37:1p	787.5727	C43 H82 O9 N1 P1
PS(40:4)	40:04:00	839.5676	C46 H82 O10 N1 P1
PS(40:8p)	40:8p	815.5101	C46 H74 O9 N1 P1
PS(44:11)	44:11:00	881.5207	C50 H76 O10 N1 P1
PC(14:0p/18:1)	14:0p/18:1	715.5516	C40 H78 O7 N1 P1
PC(17:1/18:1)	17:1/18:1	771.5778	C43 H82 O8 N1 P1
PC(18:0p/16:0)	18:0p/16:0	745.5985	C42 H84 O7 N1 P1
PC(25:0e)	25:0e	621.4733	C33 H68 O7 N1 P1
PC(25:0p)	25:0p	619.4577	C33 H66 O7 N1 P1
PC(27:0/16:0)	27:0/16:0	887.7343	C51 H102 O8 N1 P1
PC(27:0e)	27:0e	649.5046	C35 H72 O7 N1 P1
PC(27:0p)	27:0p	647.489	C35 H70 O7 N1 P1
PC(27:1/16:0)	27:1/16:0	885.7187	C51 H100 O8 N1 P1
PC(28:2e)	28:2e	659.489	C36 H70 O7 N1 P1
PC(29:0)	29:00:00	691.5152	C37 H74 O8 N1 P1
PC(29:0p)	29:0p	675.5203	C37 H74 O7 N1 P1
PC(30:1/16:0)	30:1/16:0	927.7656	C54 H106 O8 N1 P1
PC(31:0e)	31:0e	705.5672	C39 H80 O7 N1 P1
PC(31:1/16:0)	31:1/16:0	941.7813	C55 H108 O8 N1 P1
PC(31:8)	31:08:00	703.4213	C39 H62 O8 N1 P1

PC(33:2)	33:02:00	743.5465	C41 H78 O8 N1 P1
PC(33:3)	33:03:00	741.5309	C41 H76 O8 N1 P1
PC(34:0)	34:00:00	761.5935	C42 H84 O8 N1 P1
PC(34:1)	34:01:00	759.5778	C42 H82 O8 N1 P1
PC(34:4)	34:04:00	753.5309	C42 H76 O8 N1 P1
PC(35:0p)	35:0p	759.6142	C43 H86 O7 N1 P1
PC(35:1p)	35:1p	757.5985	C43 H84 O7 N1 P1
PC(35:1p)	35:1p	757.5985	C43 H84 O7 N1 P1
PC(35:4)	35:04:00	767.5465	C43 H78 O8 N1 P1
PC(35:5)	35:05:00	765.5309	C43 H76 O8 N1 P1
PC(36:0)	36:00:00	789.6248	C44 H88 O8 N1 P1
PC(37:2)	37:02:00	799.6091	C45 H86 O8 N1 P1
PC(37:2)	37:02:00	799.6091	C45 H86 O8 N1 P1
PC(37:2p)	37:2p	783.6142	C45 H86 O7 N1 P1
PC(37:4)	37:04:00	795.5778	C45 H82 O8 N1 P1
PC(37:6)	37:06:00	791.5465	C45 H78 O8 N1 P1
PC(37:6)	37:06:00	791.5465	C45 H78 O8 N1 P1
PC(38:1e)	38:1e	801.6611	C46 H92 O7 N1 P1
PC(38:4e)	38:4e	795.6142	C46 H86 O7 N1 P1
PC(38:6e)	38:6e	791.5829	C46 H82 O7 N1 P1
PC(39:1)	39:01:00	829.6561	C47 H92 O8 N1 P1
PC(39:4)	39:04:00	823.6091	C47 H86 O8 N1 P1
PC(39:6)	39:06:00	819.5778	C47 H82 O8 N1 P1
PC(40:0)	40:00:00	845.6874	C48 H96 O8 N1 P1
PC(40:1e)	40:1e	829.6924	C48 H96 O7 N1 P1
PC(40:2e)	40:2e	827.6768	C48 H94 O7 N1 P1
PC(40:8)	40:08:00	829.5622	C48 H80 O8 N1 P1
PC(40:8p)	40:8p	813.5672	C48 H80 O7 N1 P1
PC(41:0)	41:00:00	859.703	C49 H98 O8 N1 P1
PC(42:0e)	42:0e	859.7394	C50 H102 O7 N1 P1
PC(42:2e)	42:2e	855.7081	C50 H98 O7 N1 P1
PC(42:2p)	42:2p	853.6924	C50 H96 O7 N1 P1
PC(42:3)	42:03:00	867.6717	C50 H94 O8 N1 P1
PC(42:4p)	42:4p	849.6611	C50 H92 O7 N1 P1
PC(43:3)	43:03:00	881.6874	C51 H96 O8 N1 P1
PC(43:3)	43:03:00	881.6874	C51 H96 O8 N1 P1
PC(44:0)	44:00:00	901.75	C52 H104 O8 N1 P1
PC(44:2p)	44:2p	881.7237	C52 H100 O7 N1 P1
PC(44:4)	44:04:00	893.6874	C52 H96 O8 N1 P1
PC(44:4)	44:04:00	893.6874	C52 H96 O8 N1 P1
PC(44:5)	44:05:00	891.6717	C52 H94 O8 N1 P1
PC(45:3)	45:03:00	909.7187	C53 H100 O8 N1 P1
PC(45:3)	45:03:00	909.7187	C53 H100 O8 N1 P1
PC(45:4)	45:04:00	907.703	C53 H98 O8 N1 P1
PC(45:5)	45:05:00	905.6874	C53 H96 O8 N1 P1
PC(46:3)	46:03:00	923.7343	C54 H102 O8 N1 P1
PC(46:4)	46:04:00	921.7187	C54 H100 O8 N1 P1
PC(46:6)	46:06:00	917.6874	C54 H96 O8 N1 P1

PC(47:3)	47:03:00	937.75	C55 H104 O8 N1 P1
PC(47:4)	47:04:00	935.7343	C55 H102 O8 N1 P1
PC(47:5)	47:05:00	933.7187	C55 H100 O8 N1 P1
PC(48:3)	48:03:00	951.7656	C56 H106 O8 N1 P1
PC(49:2)	49:02:00	967.7969	C57 H110 O8 N1 P1
PC(50:3)	50:03:00	979.7969	C58 H110 O8 N1 P1
PC(50:5)	50:05:00	975.7656	C58 H106 O8 N1 P1
PC(52:5)	52:05:00	1003.7969	C60 H110 O8 N1 P1
PC(54:5)	54:05:00	1031.8282	C62 H114 O8 N1 P1
PC(54:6)	54:06:00	1029.8126	C62 H112 O8 N1 P1
PC(54:7)	54:07:00	1027.7969	C62 H110 O8 N1 P1
PC(58:5)	58:05:00	1087.8908	C66 H122 O8 N1 P1
PC(15:0/18:1)	15:0/18:1	745.5622	C41 H80 O8 N1 P1
PC(16:0p/16:0)	16:0p/16:0	717.5672	C40 H80 O7 N1 P1
PC(16:0p/18:1)	16:0p/18:1	743.5829	C42 H82 O7 N1 P1
PC(17:0/18:1)	17:0/18:1	773.5935	C43 H84 O8 N1 P1
PC(17:1/18:1)	17:1/18:1	771.5778	C43 H82 O8 N1 P1
PC(18:2p/18:0)	18:2p/18:0	769.5985	C44 H84 O7 N1 P1
PC(26:1/16:0)	26:1/16:0	871.703	C50 H98 O8 N1 P1
PC(26:1/17:1)	26:1/17:1	883.703	C51 H98 O8 N1 P1
PC(26:1/19:1)	26:1/19:1	911.7343	C53 H102 O8 N1 P1
PC(28:0)	28:00:00	677.4996	C36 H72 O8 N1 P1
PC(28:1/16:0)	28:1/16:0	899.7343	C52 H102 O8 N1 P1
PC(30:0)	30:00:00	705.5309	C38 H76 O8 N1 P1
PC(30:0e)	30:0e	691.5516	C38 H78 O7 N1 P1
PC(30:1)	30:01:00	703.5152	C38 H74 O8 N1 P1
PC(31:0p)	31:0p	703.5516	C39 H78 O7 N1 P1
PC(31:1)	31:01:00	717.5309	C39 H76 O8 N1 P1
PC(32:0e)	32:0e	719.5829	C40 H82 O7 N1 P1
PC(32:1)	32:01:00	731.5465	C40 H78 O8 N1 P1
PC(32:2)	32:02:00	729.5309	C40 H76 O8 N1 P1
PC(32:3)	32:03:00	727.5152	C40 H74 O8 N1 P1
PC(33:0)	33:00:00	747.5778	C41 H82 O8 N1 P1
PC(33:2)	33:02:00	743.5465	C41 H78 O8 N1 P1
PC(34:0)	34:00:00	761.5935	C42 H84 O8 N1 P1
PC(34:0e)	34:0e	747.6142	C42 H86 O7 N1 P1
PC(34:1)	34:01:00	759.5778	C42 H82 O8 N1 P1
PC(34:2)	34:02:00	757.5622	C42 H80 O8 N1 P1
PC(34:3)	34:03:00	755.5465	C42 H78 O8 N1 P1
PC(36:0)	36:00:00	789.6248	C44 H88 O8 N1 P1
PC(36:2)	36:02:00	785.5935	C44 H84 O8 N1 P1
PC(36:3)	36:03:00	783.5778	C44 H82 O8 N1 P1
PC(38:2)	38:02:00	813.6248	C46 H88 O8 N1 P1
PC(38:3)	38:03:00	811.6091	C46 H86 O8 N1 P1
PC(38:5)	38:05:00	807.5778	C46 H82 O8 N1 P1
PC(38:6)	38:06:00	805.5622	C46 H80 O8 N1 P1
PC(38:6e)	38:6e	791.5829	C46 H82 O7 N1 P1
PC(40:4)	40:04:00	837.6248	C48 H88 O8 N1 P1

PC(40:5)	40:05:00	835.6091	C48 H86 O8 N1 P1
PC(40:6)	40:06:00	833.5935	C48 H84 O8 N1 P1
PC(40:7)	40:07:00	831.5778	C48 H82 O8 N1 P1
PC(48:2)	48:02:00	953.7813	C56 H108 O8 N1 P1
PC(48:6)	48:06:00	945.7187	C56 H100 O8 N1 P1
PC(37:6)	37:06:00	791.5465	C45 H78 O8 N1 P1
PC(48:3)	48:03:00	951.7656	C56 H106 O8 N1 P1
PE(15:1)	15:01	451.2335	C20 H38 O8 N1 P1
PE(16:0p/18:1)	16:0p/18:1	701.5359	C39 H76 O7 N1 P1
PE(16:0p/18:1)	16:0p/18:1	701.5359	C39 H76 O7 N1 P1
PE(16:0p/20:3)	16:0p/20:3	725.5359	C41 H76 O7 N1 P1
PE(16:0p/22:3)	16:0p/22:3	753.5672	C43 H80 O7 N1 P1
PE(18:0/20:1)	18:0/20:1	773.5935	C43 H84 O8 N1 P1
PE(18:0/22:3)	18:0/22:3	797.5935	C45 H84 O8 N1 P1
PE(18:0e/20:1)	18:0e/20:1	759.6142	C43 H86 O7 N1 P1
PE(18:0p)	18:0p	479.3012	C23 H46 O7 N1 P1
PE(18:0p/21:1)	18:0p/21:1	771.6142	C44 H86 O7 N1 P1
PE(18:0p/22:1)	18:0p/22:1	785.6298	C45 H88 O7 N1 P1
PE(18:0p/22:3)	18:0p/22:3	781.5985	C45 H84 O7 N1 P1
PE(18:0p/22:4)	18:0p/22:4	779.5829	C45 H82 O7 N1 P1
PE(18:1/18:2)	18:1/18:2	741.5309	C41 H76 O8 N1 P1
PE(18:1/20:4)	18:1/20:4	765.5309	C43 H76 O8 N1 P1
PE(18:1p/14:0)	18:1p/14:0	673.5046	C37 H72 O7 N1 P1
PE(18:1p/16:1)	18:1p/16:1	699.5203	C39 H74 O7 N1 P1
PE(18:1p/18:1)	18:1p/18:1	727.5516	C41 H78 O7 N1 P1
PE(18:1p/18:1)	18:1p/18:1	727.5516	C41 H78 O7 N1 P1
PE(18:1p/21:1)	18:1p/21:1	769.5985	C44 H84 O7 N1 P1
PE(18:1p/22:6)	18:1p/22:6	773.5359	C45 H76 O7 N1 P1
PE(18:1p/23:1)	18:1p/23:1	797.6298	C46 H88 O7 N1 P1
PE(18:1p/24:1)	18:1p/24:1	811.6455	C47 H90 O7 N1 P1
PE(18:1p/24:2)	18:1p/24:2	809.6298	C47 H88 O7 N1 P1
PE(20:0p/22:4)	20:0p/22:4	807.6142	C47 H86 O7 N1 P1
PE(20:1/18:1)	20:1/18:1	771.5778	C43 H82 O8 N1 P1
PE(20:1p/22:4)	20:1p/22:4	805.5985	C47 H84 O7 N1 P1
PE(22:2)	22:02	547.3274	C27 H50 O8 N1 P1
PE(23:0e)	23:0e	551.3951	C28 H58 O7 N1 P1
PE(23:0p)	23:0p	549.3794	C28 H56 O7 N1 P1
PE(24:3e)	24:3e	559.3638	C29 H54 O7 N1 P1
PE(25:0e)	25:0e	579.4264	C30 H62 O7 N1 P1
PE(26:1/18:1)	26:1/18:1	855.6717	C49 H94 O8 N1 P1
PE(29:1)	29:01:00	647.4526	C34 H66 O8 N1 P1
PE(38:4e)	38:4e	753.5672	C43 H80 O7 N1 P1
PE(42:4)	42:04:00	823.6091	C47 H86 O8 N1 P1
PE(50:5)	50:05:00	933.7187	C55 H100 O8 N1 P1
PE(50:7)	50:07:00	929.6874	C55 H96 O8 N1 P1
PE(52:7)	52:07:00	957.7187	C57 H100 O8 N1 P1
PE(16:0p/18:1)	16:0p/18:1	701.5359	C39 H76 O7 N1 P1
PE(16:0p/20:4)	16:0p/20:4	723.5203	C41 H74 O7 N1 P1

PE(16:0p/22:4)	16:0p/22:4	751.5516	C43 H78 O7 N1 P1
PE(16:0p/22:6)	16:0p/22:6	747.5203	C43 H74 O7 N1 P1
PE(18:0/18:1)	18:0/18:1	745.5622	C41 H80 O8 N1 P1
PE(18:0/20:4)	18:0/20:4	767.5465	C43 H78 O8 N1 P1
PE(18:0/22:4)	18:0/22:4	795.5778	C45 H82 O8 N1 P1
PE(18:0/22:6)	18:0/22:6	791.5465	C45 H78 O8 N1 P1
PE(18:0p/20:1)	18:0p/20:1	757.5985	C43 H84 O7 N1 P1
PE(18:0p/22:4)	18:0p/22:4	779.5829	C45 H82 O7 N1 P1
PE(18:0p/22:6)	18:0p/22:6	775.5516	C45 H78 O7 N1 P1
PE(18:1/18:1)	18:1/18:1	743.5465	C41 H78 O8 N1 P1
PE(18:1p/18:1)	18:1p/18:1	727.5516	C41 H78 O7 N1 P1
PE(18:1p/20:1)	18:1p/20:1	755.5829	C43 H82 O7 N1 P1
PE(18:1p/20:4)	18:1p/20:4	749.5359	C43 H76 O7 N1 P1
PE(18:1p/22:1)	18:1p/22:1	783.6142	C45 H86 O7 N1 P1
PE(18:1p/22:4)	18:1p/22:4	777.5672	C45 H80 O7 N1 P1
PE(16:0/18:1)	16:0/18:1	717.5309	C39 H76 O8 N1 P1
PE(18:0/20:4)	18:0/20:4	767.5465	C43 H78 O8 N1 P1
PE(42:3)	42:03:00	825.6248	C47 H88 O8 N1 P1
PE(42:3p)	42:3p	809.6298	C47 H88 O7 N1 P1
PE(44:6)	44:06:00	847.6091	C49 H86 O8 N1 P1
PI(16:0/18:1)	16:0/18:1	836.5415	C43 H81 O13 N0 P1
PI(16:1)	16:01	584.2598	C25 H45 O13 N0 P1
PI(27:3)	27:03:00	734.4006	C36 H63 O13 N0 P1
PI(32:8)	32:08:00	794.4006	C41 H63 O13 N0 P1
PI(38:8)	38:08:00	878.4945	C47 H75 O13 N0 P1
PI(38:9)	38:09:00	876.4789	C47 H73 O13 N0 P1
PI(40:7)	40:07:00	908.5415	C49 H81 O13 N0 P1
PI(40:8)	40:08:00	906.5258	C49 H79 O13 N0 P1
PI(16:0/20:4)	16:0/20:4	858.5258	C45 H79 O13 N0 P1
PI(18:0/20:4)	18:0/20:4	886.5571	C47 H83 O13 N0 P1
PI(18:1/20:4)	18:1/20:4	884.5415	C47 H81 O13 N0 P1
PI(31:1p)	31:1p	778.4996	C40 H75 O12 N0 P1
PI(32:1p)	32:1p	792.5153	C41 H77 O12 N0 P1
PI(34:3p)	34:3p	816.5153	C43 H77 O12 N0 P1
PI(34:6p)	34:6p	810.4683	C43 H71 O12 N0 P1
PI(38:8)	38:08:00	878.4945	C47 H75 O13 N0 P1
PI(39:1p)	39:1p	890.6248	C48 H91 O12 N0 P1
PS(18:0/20:4)	18:0/20:4	811.5363	C44 H78 O10 N1 P1
PS(18:0/22:4)	18:0/22:4	839.5676	C46 H82 O10 N1 P1
PS(18:1/20:4)	18:1/20:4	809.5207	C44 H76 O10 N1 P1
PS(21:3)	21:03	575.2859	C27 H46 O10 N1 P1
PS(21:3)	21:03	575.2859	C27 H46 O10 N1 P1
PS(21:3)	21:03	575.2859	C27 H46 O10 N1 P1
PS(23:0)	23:00	609.3642	C29 H56 O10 N1 P1
PS(23:0)	23:00	609.3642	C29 H56 O10 N1 P1
PS(28:0p)	28:0p	663.4475	C34 H66 O9 N1 P1
PS(32:0)	32:00:00	735.505	C38 H74 O10 N1 P1
PS(32:1)	32:01:00	733.4894	C38 H72 O10 N1 P1

PS(33:7)	33:07:00	735.4111	C39 H62 O10 N1 P1
PS(39:1)	39:01:00	831.5989	C45 H86 O10 N1 P1
PS(16:0/18:1)	16:0/18:1	761.5207	C40 H76 O10 N1 P1
PS(18:0/18:1)	18:0/18:1	789.552	C42 H80 O10 N1 P1
PS(18:0/20:1)	18:0/20:1	817.5833	C44 H84 O10 N1 P1
PS(18:0/22:6)	18:0/22:6	835.5363	C46 H78 O10 N1 P1
PS(18:1/18:1)	18:1/18:1	787.5363	C42 H78 O10 N1 P1
PS(18:1/22:0)	18:1/22:0	845.6146	C46 H88 O10 N1 P1
PS(18:1/22:1)	18:1/22:1	843.5989	C46 H86 O10 N1 P1
PS(18:1/23:0)	18:1/23:0	859.6302	C47 H90 O10 N1 P1
PS(18:1/24:0)	18:1/24:0	873.6459	C48 H92 O10 N1 P1
PS(18:1/24:1)	18:1/24:1	871.6302	C48 H90 O10 N1 P1
PS(20:1/18:1)	20:1/18:1	815.5676	C44 H82 O10 N1 P1
PS(16:0p/18:1)	16:0p/18:1	745.5258	C40 H76 O9 N1 P1
PS(29:0)	29:00:00	693.4581	C35 H68 O10 N1 P1
PS(21:3)	21:03	575.2859	C27 H46 O10 N1 P1
PS(21:3)	21:03	575.2859	C27 H46 O10 N1 P1
PS(21:3)	21:03	575.2859	C27 H46 O10 N1 P1
PS(21:3)	21:03	575.2859	C27 H46 O10 N1 P1
PS(21:3)	21:03	575.2859	C27 H46 O10 N1 P1
PS(23:0)	23:00	609.3642	C29 H56 O10 N1 P1
PS(29:3)	29:03:00	687.4111	C35 H62 O10 N1 P1
PS(30:4)	30:04:00	699.4111	C36 H62 O10 N1 P1
PS(32:4e)	32:4e	713.4632	C38 H68 O9 N1 P1
PS(33:7)	33:07:00	735.4111	C39 H62 O10 N1 P1
PS(39:4)	39:04:00	825.552	C45 H80 O10 N1 P1
PS(39:4)	39:04:00	825.552	C45 H80 O10 N1 P1
PS(45:6)	45:06:00	905.6146	C51 H88 O10 N1 P1

✣Lipid identification and relative quantification were performed using LipidSearch 4.1 software (Thermo). The search criteria were as follows: product search; parent m/z tolerance 5 ppm; product m/z tolerance 5 ppm; quantification: m/z tolerance 5 ppm, retention time tolerance 1 min.

Supplemental Table 3: Phospholipid Class Comparison

	Control			Glaucoma			
**Lipid Class✣**	**Mean**	**Std. Deviation**	**95% CI**	**Mean**	**Std. Deviation**	**95% CI**	**p-Value***
PC	37.39	0.67	36.94-37.84	35.77	3.21	33.74-37.81	1.17E-01
PE	35.21	0.48	34.88-35.52	34.94	2.77	33.18-36.70	7.63E-01
PI	29.75	1.25	28.91- 30.59	29.56	0.98	28.94-30.18	6.98E-01
PS	33.37	0.61	32.96-33.78	32.81	1.42	31.91-33.71	2.37E-01

✣Lipid identification and relative quantification were performed using LipidSearch 4.1 software (Thermo). The search criteria were as follows: product search; parent m/z tolerance 5 ppm; product m/z tolerance 5ppm; quantification: m/z tolerance 5 ppm, retention time tolerance 0.5 min. T-test results (*p-values). Data presented has been log2 transformed.

Supplemental Table 4: Lipid Class Comparisions

**Lipid Class✣**	**Mean**		**Std. deviation**		**95% CI Difference**	***p-value**
	Control	*Glaucoma*	*Control*	*Glaucoma*		
Ceramides	31.83	32.18	6.05	4.28	-1.21 - 1.91	6.59E-01
Glucosylsphingosine	30.20	30.97	0.37	0.49	0.40 - 1.16	4.00E-04
Phospholipids	33.93	33.71	2.94	3.04	-1.49 - 1.05	7.33E-01
Sphingomyelin	36.90	36.12	0.71	1.57	-1.87 - 0.30	3.00E-01
Sphingoid Base/S1P	19.56	19.14	3.13	2.67	-3.00 - 2.17	7.50E-01
FA Chain Length: >24	40.56	39.69	0.46	0.46	-0.37 - 2.12	1.59E-01
FA Chain Length: 12-24	38.58	37.30	0.69	2.26	- 0.21 - 2.77	8.72E-02
FA Chain Length: <12	25.64	25.29	4.57	4.47	-4.00 - 3.31	8.46E-01
Total Lipid	1717.00	1685.00	54.80	142.30	-127 - 63.2	9.00E-01

✣Lipid identification and relative quantification were performed using LipidSearch 4.1 software (Thermo). The search criteria were as follows: product search; parent m/z tolerance 5 ppm; product m/z tolerance 5ppm; quantification: m/z tolerance 5 ppm, retention time tolerance 0.5 min. *p-values from t-tests and Wilcoxon rank-sum. Data presented has been log2 transformed.

Supplemental Table 5: Multiple Regression of Glucosylsphingosine Peak Intensity

Model	Unstandardized Coefficients✣		Standardized Coefficients	t	p-value	95% CI for B	
	B	*Std. Error*	*Beta*			*Lower Bound*	*Upper Bound*
(Constant)	28.81	1.22		23.56	0.000	26.25	31.37
Age	0.007	0.0133	0.103	0.53	0.602	-0.02	0.03
Gender	0.058	0.228	0.049	0.26	0.799	-0.42	0.53
Disease	0.784	0.192	0.687	4.07	0.001 *	0.38	1.19

✣Dependent variable: Glucosylsphingosine peak intensity. Pedictors: (constant), age, gender, and disease state (control/glaucoma). R2 = 0.47.

Supplemental Table 6: Species Level Details for Ceramides, Sphingomyelins, Sphingosines, and Glucosylsphingosine

Lipid Species✣	m/z	Mean		Standard deviation		Minimum		Maximum		Fold Change	Glaucoma /Control	*p -value
		Control	*Glaucoma*	*Control*	*Glaucom*	*Control*	*Glaucoma*	*Control*	*Glaucoma*			
Cer(d16:0+pO)	303.2	24.43	26.49	1.09	2.31	23.19	24.00	26.69	32.14	1.08	Up	1.34E-02
Cer(d16:1/22:1)	591.6	22.48	23.95	2.38	2.27	18.25	18.25	24.57	25.97	1.07	Up	7.93E-02
Cer(d16:1+hO)	301.2	10.79	15.36	5.30	0.94	2.63	14.75	15.42	18.25	1.42	Up	3.40E-03
Cer(d17:0+pO/33:1)	777.8	10.43	24.08	9.92	1.70	2.63	21.80	23.74	27.04	2.31	Up	2.20E-03
Cer(d17:0+pO/35:2)	803.8	24.05	25.71	1.19	1.72	21.57	23.65	25.78	28.47	1.07	Up	1.43E-02
Cer(d17:0+pO/35:3)	801.8	22.80	15.36	1.25	0.94	20.43	14.75	24.40	18.25	-1.48	Down	1.00E-04
Cer(d17:0+pO/37:2)	831.8	10.58	24.17	10.14	1.91	2.63	22.11	24.49	27.20	2.29	Up	4.90E-03
Cer(d18:0/24:0)	651.7	22.45	24.43	3.41	2.40	18.68	18.25	26.93	26.51	1.09	Up	5.25E-01
Cer(d18:0/24:0+O)	667.6	11.30	24.87	11.13	2.45	2.63	18.25	26.21	26.56	2.2	Up	4.00E-03
Cer(d18:0/24:1)	649.6	24.42	27.61	3.86	1.05	17.39	25.88	27.44	29.27	1.13	Up	4.20E-02
Cer(d18:0/50:2EO)	1042	11.44	15.36	9.34	0.94	2.63	14.75	22.50	18.25	1.34	Up	6.88E-01
Cer(d18:0/52:2EO)	1070	10.80	15.36	8.80	0.94	2.63	14.75	22.14	18.25	1.42	Up	2.80E-01
Cer(d18:0+pO)	331.3	17.99	23.55	2.89	1.70	12.61	21.16	21.42	28.07	1.31	Up	1.00E-04
Cer(d18:0+pO/18:0)	583.6	26.63	28.49	2.81	1.13	21.48	26.05	29.76	29.96	1.07	Up	6.88E-02
Cer(d18:0+pO/18:1)	581.5	23.75	23.85	0.85	2.24	21.94	18.25	25.11	26.34	1	Up	8.87E-01
Cer(d18:0+pO/22:0)	639.6	26.27	26.71	0.81	2.74	24.79	18.25	27.87	28.57	1.02	Up	7.00E-03
Cer(d18:0+pO/23:0)	653.6	23.54	25.11	2.89	2.30	18.93	18.25	26.13	27.10	1.07	Up	3.56E-01
Cer(d18:0+pO/23:1)	651.6	24.45	15.36	1.48	0.94	22.22	14.75	27.31	18.25	-1.59	Down	1.00E-04
Cer(d18:0+pO/24:0)	667.6	27.23	28.43	0.98	1.04	25.46	26.70	28.48	29.76	1.04	Up	9.50E-03
Cer(d18:0+pO/24:1)	665.6	30.27	28.62	0.70	1.68	29.20	24.73	32.02	30.51	-1.06	Down	7.20E-03
Cer(d18:0+pO/25:1)	679.6	27.57	26.39	1.16	1.67	24.52	22.78	29.22	28.37	-1.04	Down	4.39E-02
Cer(d18:0+pO/26:1)	693.7	26.18	15.36	1.36	0.94	22.81	14.75	27.61	18.25	-1.7	Down	1.00E-04
Cer(d18:0+pO/26:2)	691.6	24.77	25.27	2.94	2.65	18.39	18.25	27.09	27.66	1.02	Up	8.33E-01
Cer(d18:0+pO/36:3)	829.8	12.81	15.36	9.35	0.94	2.63	14.75	24.43	18.25	1.2	Up	8.53E-01
Cer(d18:1)	313.3	21.89	23.33	3.21	2.08	18.47	21.27	28.30	27.88	1.07	Up	2.12E-01
Cer(d18:1/16:0)	537.5	25.01	27.31	2.29	0.92	20.04	25.29	27.65	28.65	1.09	Up	8.40E-03
Cer(d18:1/18:0)	565.5	29.09	27.92	0.53	3.24	28.35	18.25	29.90	30.01	-1.04	Down	6.44E-01
Cer(d18:1/18:1)	563.5	24.85	24.22	1.13	2.30	23.88	18.25	27.44	26.58	-1.03	Down	9.75E-01
Cer(d18:1/20:0)	593.6	26.54	26.61	0.44	2.73	25.45	18.25	27.14	28.35	1	Up	2.68E-02
Cer(d18:1/22:0)	621.6	26.35	26.71	0.63	2.74	25.49	18.25	27.89	28.48	1.01	Up	5.60E-03

Cer(d18:1/22:1)	619.6	26.04	25.61	1.16	2.74	22.71	18.25	26.85	27.66	-1.02	Down	6.08E-01
Cer(d18:1/23:0)	635.6	26.18	25.80	1.08	2.51	23.74	18.25	28.37	27.97	-1.01	Down	9.02E-01
Cer(d18:1/23:1)	633.6	27.09	27.55	0.51	1.58	26.07	23.70	28.07	29.14	1.02	Up	3.58E-01
Cer(d18:1/24:0)	649.6	26.33	15.36	0.81	0.94	25.45	14.75	28.59	18.25	-1.71	Down	1.00E-04
Cer(d18:1/24:0+O)	665.6	17.69	23.38	8.72	1.82	2.63	18.25	24.44	25.46	1.32	Up	9.05E-02
Cer(d18:1/24:1)	647.6	30.56	29.40	0.98	1.84	29.16	25.58	32.77	31.51	-1.04	Down	7.78E-02
Cer(d18:1/24:2)	645.6	28.13	28.07	0.44	1.74	27.60	25.18	28.88	30.30	-1	Down	9.17E-01
Cer(d18:1/25:0)	663.7	24.76	15.36	2.12	0.94	19.19	14.75	27.19	18.25	-1.61	Down	1.00E-04
Cer(d18:1/25:1)	661.6	27.89	25.98	0.78	3.10	26.32	18.25	29.58	29.36	-1.07	Down	5.92E-02
Cer(d18:1/26:1)	675.7	26.71	25.83	1.32	2.97	23.45	18.25	28.87	28.62	-1.03	Down	7.86E-01
Cer(d18:1/26:2)	673.6	26.54	26.61	0.73	3.12	25.30	18.25	27.56	29.10	1	Up	2.55E-01
Cer(d18:1/52:2EO)	1068	12.80	15.36	9.24	0.94	2.63	14.75	23.71	18.25	1.2	Up	9.26E-01
Cer(d18:1/56:2EO)	1124	10.99	15.36	8.80	0.94	2.63	14.75	22.13	18.25	1.4	Up	6.88E-01
Cer(d18:1+hO)	329.3	16.84	22.90	2.54	1.84	13.07	20.91	22.85	27.35	1.36	Up	1.00E-04
Cer(d18:1+hO/24:1)	663.6	26.93	26.83	2.85	2.81	19.52	18.25	29.32	28.59	-1	Down	5.59E-01
Cer(d18:2/18:0)	563.5	22.95	26.10	4.07	2.69	15.50	18.25	27.95	29.22	1.14	Up	8.60E-03
Cer(d18:2/18:1)	561.5	18.80	15.36	5.72	0.94	2.63	14.75	22.26	18.25	-1.22	Down	1.90E-03
Cer(d18:2/24:1)	645.6	22.76	26.01	4.67	2.58	14.98	18.25	26.58	27.71	1.14	Up	1.56E-02
Cer(d18:2/25:1)	659.6	18.43	15.36	6.49	0.94	2.63	14.75	23.00	18.25	-1.2	Down	1.32E-01
Cer(d19:0)	329.3	18.27	20.71	3.63	4.61	12.76	15.00	25.35	27.13	1.13	Up	1.77E-01
Cer(d19:1+hO)	343.3	17.30	22.95	1.43	1.62	14.25	21.59	18.50	27.95	1.33	Up	1.00E-04
Cer(d20:0+pO)	359.3	22.47	24.51	1.70	0.92	18.53	23.01	24.52	25.91	1.09	Up	1.60E-03
Cer(d20:1)	341.3	22.43	25.18	1.43	1.10	20.88	23.53	24.08	27.01	1.12	Up	2.00E-04
Cer(d20:1+hO)	357.3	26.15	27.37	1.09	0.70	25.00	25.79	27.47	28.23	1.05	Up	6.70E-03
Cer(d20:2)	339.3	26.21	24.16	1.15	2.88	25.00	21.27	27.69	28.27	-1.08	Down	1.34E-01
Cer(d21:0)	357.3	16.15	23.08	1.92	2.41	13.46	21.09	19.65	30.19	1.43	Up	1.00E-04
Cer(d21:0+pO)	373.3	19.62	23.65	0.96	2.22	18.27	21.42	21.11	29.47	1.21	Up	1.00E-04
Cer(d21:1)	355.3	19.10	20.44	2.69	2.66	15.89	15.15	23.82	23.21	1.07	Up	1.86E-01
Cer(d21:1+hO)	371.3	18.26	15.36	1.79	0.94	15.31	14.75	20.44	18.25	-1.19	Down	1.00E-04
Cer(d22:0)	371.3	20.60	15.36	1.36	0.94	19.38	14.75	24.16	18.25	-1.34	Down	1.00E-04
Cer(d22:0+pO)	387.3	21.37	24.32	1.04	1.36	19.84	23.36	23.22	28.42	1.14	Up	1.00E-04
Cer(d22:1)	369.3	17.55	15.36	2.69	0.94	13.51	14.75	23.28	18.25	-1.14	Down	2.68E-02
Cer(d22:1+hO)	385.3	24.90	26.98	1.23	0.51	23.39	26.19	26.73	27.69	1.08	Up	2.00E-04
Cer(d22:2)	367.3	25.14	26.51	1.13	3.68	24.03	22.50	27.01	32.14	1.05	Up	2.41E-01
Cer(d23:1)	383.3	12.76	19.36	5.33	3.83	2.63	14.75	19.69	25.25	1.52	Up	1.50E-03
Cer(d24:1+hO)	413.4	22.16	23.57	1.30	1.03	20.12	22.16	23.82	25.85	1.06	Up	8.40E-03
Cer(d24:2)	395.3	22.33	25.08	1.58	3.79	19.95	21.25	24.82	31.40	1.12	Up	1.03E-01
Cer(d25:0+pO)	429.4	17.37	24.74	1.41	2.16	15.22	22.84	19.03	30.60	1.42	Up	1.00E-04

Cer(d25:1)	411.4	18.43	20.87	2.30	3.63	15.89	14.86	22.88	25.22	1.13	Up	9.05E-02
Cer(d26:0)	427.4	18.07	22.25	5.40	1.47	2.63	18.25	21.50	24.27	1.23	Up	5.00E-04
Cer(d26:2)	423.4	18.99	25.11	5.12	3.79	5.89	18.25	24.75	30.85	1.32	Up	2.20E-03
Cer(d27:0+O)	457.4	23.89	25.08	3.11	2.37	18.27	21.91	28.50	28.41	1.05	Up	3.12E-01
Cer(d27:1)	439.4	24.01	25.05	3.08	2.36	18.28	21.88	28.65	28.40	1.04	Up	3.68E-01
Cer(d28:0)	455.4	18.84	22.93	8.48	3.22	2.63	14.95	24.41	26.19	1.22	Up	2.81E-01
Cer(d28:1)	453.4	11.86	15.36	6.91	0.94	2.63	14.75	19.06	18.25	1.3	Up	6.01E-01
Cer(d29:0+pO)	485.4	23.20	24.81	2.95	2.23	18.39	21.96	27.58	27.87	1.07	Up	1.52E-01
Cer(d29:1)	467.4	23.41	24.81	2.88	2.23	18.40	21.96	27.68	27.86	1.06	Up	2.04E-01
Cer(d29:1+pO+2O)	515.4	9.78	15.36	6.50	0.94	2.63	14.75	19.54	18.25	1.57	Up	1.75E-02
Cer(d29:2)	465.4	17.38	19.27	4.63	3.93	5.89	14.86	22.73	24.77	1.11	Up	6.08E-01
Cer(d29:2+hO)	481.4	12.91	17.83	6.32	3.55	2.63	14.86	19.95	23.30	1.38	Up	1.50E-02
Cer(d30:0+pO+2O)	531.4	25.16	15.36	3.97	0.94	18.34	14.75	29.32	18.25	-1.64	Down	1.00E-04
Cer(d30:3)	477.4	7.41	19.86	9.51	5.28	2.63	15.00	26.99	27.47	2.68	Up	6.60E-03
Cer(d30:4)	475.4	15.97	15.36	7.44	0.94	2.63	14.75	24.90	18.25	-1.04	Down	5.38E-01
Cer(d30:4+pO)	491.4	15.70	22.93	7.20	3.27	2.63	15.00	26.60	27.60	1.46	Up	8.70E-03
Cer(d31:1)	495.5	9.65	17.78	7.74	3.46	2.63	14.86	19.93	23.04	1.84	Up	1.22E-02
Cer(d31:1+pO+2O)	543.4	19.73	23.39	1.56	2.40	16.79	18.25	22.97	27.33	1.19	Up	3.00E-04
Cer(d31:3)	491.4	13.15	22.94	7.81	5.45	2.63	14.75	23.65	32.02	1.74	Up	2.10E-03
Cer(d31:4)	489.4	15.27	21.71	5.91	2.60	2.63	14.95	21.60	23.77	1.42	Up	3.00E-04
Cer(d31:4+pO)	505.4	15.99	18.49	4.30	3.75	5.89	14.86	20.92	23.96	1.16	Up	3.89E-01
Cer(d32:0+pO+2O)	559.5	28.55	15.36	1.13	0.94	26.48	14.75	30.47	18.25	-1.86	Down	1.00E-04
Cer(d32:1)	509.5	15.04	23.16	7.06	1.78	2.63	18.25	22.17	25.23	1.54	Up	2.00E-04
Cer(d32:1+pO+2O)	557.5	30.49	31.88	0.83	1.22	29.38	29.94	32.35	33.63	1.05	Up	4.70E-03
Cer(d32:3)	505.4	18.01	22.57	10.89	6.64	2.63	15.11	30.22	31.51	1.25	Up	4.06E-01
Cer(d32:3+hO+2O)	553.4	21.06	24.95	1.48	1.96	19.45	23.00	23.84	30.35	1.18	Up	1.00E-04
Cer(d32:5)	501.4	17.09	20.92	4.31	3.03	5.89	15.07	21.98	23.72	1.22	Up	2.25E-02
Cer(d33:1+pO+2O)	571.5	19.08	24.34	2.31	1.79	17.08	22.29	24.03	28.16	1.28	Up	1.00E-04
Cer(d34:0)	539.5	8.95	21.50	8.81	4.37	2.63	15.00	26.08	27.06	2.4	Up	4.00E-03
Cer(d34:0+O)	555.5	22.44	25.65	2.26	3.03	19.14	21.59	26.37	30.25	1.14	Up	9.50E-03
Cer(d34:0+pO)	555.5	26.79	26.63	0.76	2.85	26.02	18.25	28.46	28.81	-1.01	Down	3.56E-01
Cer(d34:0+pO+2O)	587.5	26.40	26.67	0.76	1.16	25.43	25.08	27.94	29.10	1.01	Up	5.17E-01
Cer(d34:1)	537.5	26.50	19.21	1.51	5.50	24.79	14.97	28.67	28.55	-1.38	Down	8.90E-03
Cer(d34:1+hO)	553.5	19.66	23.91	6.13	2.03	2.63	18.25	23.98	25.84	1.22	Up	2.80E-03
Cer(d34:2)	535.5	19.48	23.85	6.05	2.02	2.63	18.25	24.02	25.81	1.22	Up	2.30E-03
Cer(d34:2+pO+2O)	583.5	27.32	23.96	2.83	3.98	22.46	15.07	30.22	30.15	-1.14	Down	3.63E-02
Cer(d34:3)	533.5	20.45	24.53	4.64	2.77	12.13	21.20	26.95	29.04	1.2	Up	1.73E-02
Cer(d34:3+hO+2O)	581.5	24.71	27.89	3.58	2.00	19.37	24.93	28.07	30.51	1.13	Up	5.95E-02

Cer(d35:0)	553.5	17.69	22.94	5.26	2.81	5.89	15.59	25.25	26.44	1.3	Up	6.50E-03
Cer(d35:0+O)	569.5	19.21	23.50	2.64	2.19	16.72	21.23	22.93	28.63	1.22	Up	4.50E-03
Cer(d35:1+hO+O)	583.5	25.03	27.99	1.70	2.74	23.12	23.75	28.69	33.63	1.12	Up	5.80E-03
Cer(d35:1+pO+2O)	599.5	17.03	26.02	5.65	2.14	2.63	23.25	24.23	31.14	1.53	Up	1.00E-04
Cer(d35:2+hO)	565.5	19.42	27.10	6.40	2.65	5.89	23.22	28.57	32.55	1.4	Up	2.60E-03
Cer(d35:2+pO+2O)	597.5	12.94	15.36	7.81	0.94	2.63	14.75	22.06	18.25	1.19	Up	8.29E-01
Cer(d35:3)	547.5	25.03	27.96	1.68	2.78	23.13	23.75	28.63	33.46	1.12	Up	6.40E-03
Cer(d36:0)	567.6	21.69	26.86	3.97	2.85	16.50	18.25	29.36	29.04	1.24	Up	1.88E-02
Cer(d36:0+O)	583.6	20.56	26.29	3.52	2.84	13.03	22.81	24.12	30.45	1.28	Up	3.00E-04
Cer(d36:0+pO)	583.6	27.78	25.68	0.55	1.49	26.85	23.40	29.07	27.73	-1.08	Down	4.00E-04
Cer(d36:0+pO+O)	599.5	20.08	15.36	1.35	0.94	17.08	14.75	21.80	18.25	-1.31	Down	1.00E-04
Cer(d36:1)	565.5	22.56	23.19	1.16	3.02	21.00	15.07	25.07	26.44	1.03	Up	1.18E-01
Cer(d36:1+hO)	581.5	24.85	26.08	2.47	2.68	18.88	18.25	28.05	29.16	1.05	Up	5.25E-02
Cer(d36:1+hO+2O)	613.5	24.89	15.36	1.92	0.94	20.80	14.75	27.52	18.25	-1.62	Down	1.00E-04
Cer(d36:2+hO+2O)	611.5	21.48	15.36	1.95	0.94	18.28	14.75	24.60	18.25	-1.4	Down	1.00E-04
Cer(d36:3)	561.5	19.55	21.18	8.21	6.92	2.63	15.00	29.82	31.06	1.08	Up	6.44E-01
Cer(d36:3+hO+2O)	609.5	27.01	29.34	1.23	1.10	25.73	26.97	28.65	30.82	1.09	Up	8.00E-04
Cer(d37:0)	581.6	18.30	15.36	5.02	0.94	5.89	14.75	24.71	18.25	-1.19	Down	1.70E-03
Cer(d37:0+O)	597.6	20.72	24.46	2.53	2.36	17.87	21.61	24.44	29.38	1.18	Up	8.60E-03
Cer(d37:1+hO+O)	611.5	24.41	26.73	1.05	2.77	22.38	22.90	25.53	31.45	1.09	Up	1.70E-02
Cer(d37:2+hO+O)	609.5	21.25	25.72	0.83	2.83	19.67	21.61	22.26	30.88	1.21	Up	2.00E-04
Cer(d37:4+pO)	589.5	8.99	15.36	9.10	0.94	2.63	14.75	24.49	18.25	1.71	Up	2.01E-01
Cer(d37:5)	571.5	8.96	15.36	9.07	0.94	2.63	14.75	23.50	18.25	1.72	Up	1.80E-01
Cer(d38:0)	595.6	25.19	18.40	1.37	2.92	23.10	14.86	27.85	21.72	-1.37	Down	1.00E-04
Cer(d38:0+O)	611.6	24.36	27.26	2.93	3.10	20.20	23.21	28.90	31.33	1.12	Up	3.16E-02
Cer(d38:0+pO)	611.6	26.61	26.64	0.39	2.78	25.88	18.25	27.28	29.08	1	Up	7.92E-02
Cer(d38:0+pO+O)	627.6	19.95	24.17	2.82	2.54	16.03	21.25	24.57	28.88	1.21	Up	1.10E-03
Cer(d38:1)	593.6	15.26	22.48	8.70	3.08	2.63	15.11	28.52	26.82	1.47	Up	2.25E-02
Cer(d38:1+pO+2O)	641.6	21.11	23.03	2.21	1.56	16.00	18.25	23.23	24.16	1.09	Up	1.30E-03
Cer(d38:2+pO+2O)	639.5	21.60	15.36	0.79	0.94	20.49	14.75	23.12	18.25	-1.41	Down	1.00E-04
Cer(d38:3+hO+2O)	637.5	21.05	15.36	1.62	0.94	18.79	14.75	23.72	18.25	-1.37	Down	1.00E-04
Cer(d38:7+hO)	597.5	19.80	20.87	5.81	3.70	2.63	14.95	24.27	24.71	1.05	Up	3.47E-01
Cer(d39:0)	609.6	21.07	15.36	1.99	0.94	18.88	14.75	25.14	18.25	-1.37	Down	1.00E-04
Cer(d39:0+pO+O)	641.6	24.53	28.12	1.19	2.64	22.57	23.73	26.28	32.91	1.15	Up	6.00E-04
Cer(d39:1)	607.6	14.79	15.36	4.40	0.94	4.21	14.75	21.60	18.25	1.04	Up	7.12E-01
Cer(d39:1+hO+O)	639.6	24.87	25.67	1.04	2.44	23.15	22.44	26.28	30.71	1.03	Up	3.16E-01
Cer(d39:6)	597.5	17.35	22.65	6.68	2.38	4.21	18.25	24.84	27.02	1.31	Up	2.81E-02
Cer(d39:6+hO)	613.5	19.44	22.49	9.32	4.94	4.21	15.00	31.51	30.68	1.16	Up	3.48E-01

Cer(d39:7+hO)	611.5	24.42	24.73	4.89	6.79	16.08	15.00	29.40	32.97	1.01	Up	8.80E-01
Cer(d40:0)	623.6	25.63	19.67	1.72	4.77	23.39	14.97	29.55	30.19	-1.3	Down	4.00E-04
Cer(d40:0+O)	639.6	16.54	23.74	4.80	2.22	4.21	21.52	22.53	29.84	1.44	Up	1.00E-04
Cer(d40:0+pO+2O)	671.6	21.61	22.15	1.77	3.77	19.72	14.95	24.12	26.07	1.02	Up	3.47E-01
Cer(d40:0+pO+O)	655.6	18.36	23.46	3.63	3.45	14.91	15.59	25.05	28.67	1.28	Up	5.60E-03
Cer(d40:1+hO)	637.6	24.83	15.36	0.86	0.94	23.87	14.75	26.46	18.25	-1.62	Down	1.00E-04
Cer(d40:1+pO)	637.6	22.07	25.05	4.07	2.19	15.69	18.25	25.55	26.47	1.14	Up	2.80E-03
Cer(d40:1+pO+2O)	669.6	18.66	15.36	1.92	0.94	16.26	14.75	22.01	18.25	-1.21	Down	1.00E-04
Cer(d40:2+pO+2O)	667.6	19.68	21.80	3.00	1.25	15.57	18.25	25.98	23.16	1.11	Up	2.25E-02
Cer(d40:6+hO)	627.5	21.56	23.16	1.81	1.89	18.34	18.25	24.55	25.63	1.07	Up	2.25E-02
Cer(d40:6+pO)	627.5	14.34	21.38	7.50	2.44	2.63	15.58	25.11	24.89	1.49	Up	1.17E-02
Cer(d40:8)	607.5	17.46	15.36	5.22	0.94	2.63	14.75	22.37	18.25	-1.14	Down	1.90E-03
Cer(d41:0)	637.6	23.96	24.63	0.77	1.45	22.66	21.86	24.96	26.49	1.03	Up	1.87E-01
Cer(d41:0+pO)	653.6	25.16	15.36	1.29	0.94	23.73	14.75	28.35	18.25	-1.64	Down	1.00E-04
Cer(d41:1+hO+O)	667.6	22.35	25.75	1.08	2.28	20.37	23.03	24.59	31.55	1.15	Up	3.00E-04
Cer(d41:1+O)	651.6	22.25	15.36	4.55	0.94	15.42	14.75	25.98	18.25	-1.45	Down	1.00E-04
Cer(d41:4+pO)	645.6	19.01	15.36	4.05	0.94	13.09	14.75	24.32	18.25	-1.24	Down	7.92E-02
Cer(d42:0)	651.7	23.53	22.92	3.28	4.15	16.68	15.15	26.00	28.09	-1.03	Down	6.44E-01
Cer(d42:1)	649.6	18.28	25.34	8.62	2.47	2.63	18.25	26.54	27.05	1.39	Up	1.38E-02
Cer(d42:1+O)	665.6	20.32	25.02	1.50	1.59	16.70	22.17	21.52	27.23	1.23	Up	1.00E-04
Cer(d42:1+pO)	665.6	19.89	15.36	1.71	0.94	16.55	14.75	21.27	18.25	-1.3	Down	1.00E-04
Cer(d42:1+pO+2O)	697.6	21.32	15.36	1.33	0.94	19.10	14.75	23.19	18.25	-1.39	Down	1.00E-04
Cer(d42:2)	647.6	21.09	23.82	2.15	1.80	15.24	21.45	23.12	27.02	1.13	Up	2.80E-03
Cer(d42:2+pO+2O)	695.6	20.83	15.36	3.74	0.94	15.73	14.75	25.60	18.25	-1.36	Down	1.00E-04
Cer(d42:3)	645.6	19.75	22.03	6.66	1.20	5.89	20.95	25.46	25.37	1.12	Up	9.76E-01
Cer(d42:5+pO)	657.6	19.23	24.64	2.69	1.91	17.15	21.59	25.43	28.59	1.28	Up	8.00E-04
Cer(d42:6+hO)	655.6	21.39	15.36	2.58	0.94	19.16	14.75	25.44	18.25	-1.39	Down	1.00E-04
Cer(d42:6+pO)	655.6	21.38	24.95	4.49	2.20	12.99	18.25	24.91	26.97	1.17	Up	5.00E-04
Cer(d42:7+hO)	653.5	23.54	24.69	0.77	2.32	22.25	18.25	24.84	27.60	1.05	Up	5.60E-03
Cer(d43:0)	665.7	22.09	24.30	0.81	1.23	21.12	21.88	23.53	26.55	1.1	Up	1.00E-04
Cer(d43:0+pO)	681.7	25.34	15.36	0.87	0.94	24.18	14.75	27.20	18.25	-1.65	Down	1.00E-04
Cer(d43:1+pO+2O)	711.6	22.42	15.36	1.20	0.94	19.83	14.75	24.32	18.25	-1.46	Down	1.00E-04
Cer(d43:2+hO)	677.6	22.07	15.36	2.04	0.94	17.76	14.75	23.99	18.25	-1.44	Down	1.00E-04
Cer(d43:2+pO+2O)	709.6	24.07	23.76	0.96	1.90	22.56	18.25	25.99	25.42	-1.01	Down	8.33E-01
Cer(d43:3+pO)	675.6	22.21	15.36	1.29	0.94	19.06	14.75	24.13	18.25	-1.45	Down	1.00E-04
Cer(d43:4+pO)	673.6	20.25	15.36	4.61	0.94	13.57	14.75	24.13	18.25	-1.32	Down	7.92E-02
Cer(d43:5+pO)	671.6	24.52	25.00	1.14	0.96	22.63	23.77	26.51	26.13	1.02	Up	5.25E-01
Cer(d44:0)	679.7	20.03	23.91	6.10	1.86	2.63	18.25	24.34	25.31	1.19	Up	1.20E-03

Cer(d44:1)	677.7	24.10	25.45	0.83	0.72	22.62	24.19	25.42	26.71	1.06	Up	4.00E-04
Cer(d44:4+pO)	687.6	25.57	25.58	0.83	2.39	24.29	18.25	27.15	27.33	1	Up	1.51E-01
Cer(d44:5+pO)	685.6	18.60	24.30	2.94	1.96	15.31	22.37	24.37	29.46	1.31	Up	4.00E-04
Cer(d44:6+pO)	683.6	22.95	24.84	1.49	0.54	18.95	24.06	24.78	25.69	1.08	Up	4.00E-04
Cer(d44:7+hO)	681.6	23.64	24.68	0.71	2.39	22.90	18.25	25.14	27.49	1.04	Up	8.60E-03
Cer(d45:4+pO)	701.6	22.70	22.32	1.37	2.56	20.26	15.58	25.14	26.77	-1.02	Down	7.35E-01
Cer(d45:5)	683.6	19.99	23.81	3.46	1.88	14.03	18.25	23.50	25.49	1.19	Up	2.00E-04
Cer(d45:5+pO)	699.6	24.29	24.34	1.29	1.77	22.19	22.13	26.46	29.27	1	Up	6.08E-01
Cer(d45:7)	679.6	13.92	17.31	10.07	3.47	2.63	14.86	25.95	24.75	1.24	Up	7.34E-01
Cer(d46:1)	705.7	17.23	15.36	5.38	0.94	2.63	14.75	20.98	18.25	-1.12	Down	2.67E-02
Cer(d47:5)	711.7	20.07	23.83	3.60	1.84	13.80	18.25	23.48	25.31	1.19	Up	1.00E-04
Cer(d47:6)	709.6	26.45	26.71	0.68	2.73	25.25	18.25	27.54	28.48	1.01	Up	1.50E-02
Cer(d47:7)	707.6	22.16	24.22	2.45	2.04	16.33	18.25	25.07	25.88	1.09	Up	1.30E-03
Cer(d47:7+hO)	723.6	15.07	15.36	5.85	0.94	2.63	14.75	21.74	18.25	1.02	Up	1.90E-01
Cer(d48:6)	723.7	23.23	23.73	1.23	2.03	21.04	18.25	25.34	26.47	1.02	Up	1.90E-01
Cer(d48:7)	721.6	8.52	15.36	7.58	0.94	2.63	14.75	20.92	18.25	1.8	Up	5.92E-02
Cer(d48:7+hO)	737.6	21.55	21.18	1.50	3.14	19.71	14.86	24.33	23.83	-1.02	Down	4.87E-01
Cer(d52:0)	791.8	13.96	15.36	9.27	0.94	2.63	14.75	24.77	18.25	1.1	Up	7.82E-01
Cer(d52:1+hO+O)	821.8	12.65	15.36	10.78	0.94	2.63	14.75	25.72	18.25	1.21	Up	7.34E-01
Cer(d52:7)	777.7	20.39	15.36	0.99	0.94	18.89	14.75	21.86	18.25	-1.33	Down	1.00E-04
Cer(d52:8)	775.7	23.68	28.47	3.61	1.55	17.05	26.41	28.32	31.62	1.2	Up	1.20E-03
Cer(d53:1)	803.8	9.34	15.36	7.66	0.94	2.63	14.75	20.29	18.25	1.64	Up	2.28E-01
Cer(d53:1+hO+O)	835.8	12.20	15.36	10.50	0.94	2.63	14.75	24.98	18.25	1.26	Up	7.34E-01
Cer(d53:6)	793.7	4.49	15.36	4.69	0.94	2.63	14.75	18.27	18.25	3.42	Up	8.00E-04
Cer(d53:8)	789.7	13.14	15.36	9.65	0.94	2.63	14.75	23.58	18.25	1.17	Up	7.81E-01
Cer(d54:0)	819.8	10.23	15.36	9.71	0.94	2.63	14.75	24.89	18.25	1.5	Up	2.79E-01
Cer(d54:2+pO)	831.8	16.59	15.36	8.40	0.94	2.63	14.75	23.27	18.25	-1.08	Down	2.81E-01
Cer(d54:3+pO)	829.8	17.04	23.53	8.10	2.41	2.63	18.25	23.99	27.46	1.38	Up	2.66E-02
Cer(d54:8)	803.7	24.51	22.35	4.63	4.90	17.21	14.75	28.90	27.71	-1.1	Down	1.18E-01
Cer(d55:1+hO+O)	863.8	26.77	15.36	1.24	0.94	24.04	14.75	28.38	18.25	-1.74	Down	1.00E-04
Cer(d56:0)	847.9	9.21	15.36	8.61	0.94	2.63	14.75	24.17	18.25	1.67	Up	1.84E-01
Cer(d56:0+pO+O)	879.9	8.02	15.36	6.63	0.94	2.63	14.75	17.28	18.25	1.92	Up	4.44E-02
Cer(d56:1+pOEO)	891.8	29.81	27.32	1.48	3.02	26.54	21.84	31.74	30.63	-1.09	Down	2.17E-02
Cer(d56:2+pOEO)	889.8	29.67	22.82	0.78	4.02	28.35	15.07	31.03	28.76	-1.3	Down	1.00E-04
Cer(d56:8)	831.7	25.25	29.77	6.07	1.71	15.94	26.88	30.54	31.83	1.18	Up	4.54E-02
Cer(d57:0+pOEO)	907.9	25.06	15.36	5.42	0.94	14.33	14.75	29.24	18.25	-1.63	Down	1.20E-03
Cer(d57:1+pOEO)	905.8	23.76	29.97	8.69	2.93	5.89	22.49	32.91	32.77	1.26	Up	1.90E-01
Cer(d57:1EO)	889.8	26.43	15.36	1.39	0.94	23.13	14.75	28.07	18.25	-1.72	Down	1.00E-04

Cer(d57:2+pOEO)	903.8	34.37	28.31	0.79	3.37	33.29	21.46	35.78	33.29	-1.21	Down	1.00E-04
Cer(d57:2EO)	887.8	26.57	15.36	1.84	0.94	23.08	14.75	29.42	18.25	-1.73	Down	1.00E-04
Cer(d58:0)	875.9	11.75	15.36	8.86	0.94	2.63	14.75	23.33	18.25	1.31	Up	6.88E-01
Cer(d58:1+pOEO)	919.9	21.09	15.36	6.75	0.94	4.21	14.75	28.16	18.25	-1.37	Down	4.00E-04
Cer(d58:2+pOEO)	917.8	20.24	15.36	8.96	0.94	2.63	14.75	28.25	18.25	-1.32	Down	1.29E-02
Cer(d58:3+hOEO)	915.8	22.99	22.91	4.71	3.75	16.99	15.07	29.82	27.94	-1	Down	9.64E-01
Cer(d58:7)	861.8	23.71	15.36	7.74	0.94	2.63	14.75	29.47	18.25	-1.54	Down	1.00E-03
Cer(d58:7+hO)	877.8	25.21	15.36	4.13	0.94	18.36	14.75	28.87	18.25	-1.64	Down	1.00E-04
Cer(d58:8)	859.8	27.18	32.48	5.24	0.98	19.64	30.82	31.93	33.46	1.19	Up	9.00E-04
Cer(d59:1+pOEO)	933.9	26.96	15.36	3.31	0.94	18.61	14.75	29.32	18.25	-1.75	Down	1.00E-04
Cer(d59:1EO)	917.9	15.20	15.36	8.39	0.94	2.63	14.75	23.73	18.25	1.01	Up	5.18E-01
Cer(d59:2EO)	915.9	22.09	15.36	2.83	0.94	17.21	14.75	26.37	18.25	-1.44	Down	1.00E-04
Cer(d59:3+hOEO)	929.8	31.30	33.00	1.26	1.83	29.55	28.11	32.77	34.86	1.05	Up	8.80E-03
Cer(d59:3EO)	913.8	22.89	15.36	3.19	0.94	19.06	14.75	26.82	18.25	-1.49	Down	1.00E-04
Cer(d60:0+pOEO)	949.9	19.60	15.36	2.59	0.94	16.49	14.75	23.42	18.25	-1.28	Down	1.00E-04
Cer(d60:3+hOEO)	943.9	15.23	15.36	10.40	0.94	2.63	14.75	26.49	18.25	1.01	Up	4.06E-01
Cer(d60:8)	887.8	27.38	31.82	5.59	0.92	17.07	30.45	31.68	33.63	1.16	Up	5.60E-03
Cer(d61:2EO)	943.9	12.89	15.36	11.02	0.94	2.63	14.75	26.19	18.25	1.19	Up	7.34E-01
Cer(d61:3)	911.9	4.97	15.36	4.14	0.94	2.63	14.75	13.70	18.25	3.09	Up	1.00E-04
Cer(d61:3+hOEO)	957.9	21.88	15.36	3.93	0.94	17.12	14.75	27.04	18.25	-1.42	Down	1.00E-04
Cer(d61:3EO)	941.9	21.38	15.36	3.27	0.94	17.29	14.75	25.89	18.25	-1.39	Down	1.00E-04
Cer(d62:1)	930	5.23	15.36	4.73	0.94	2.63	14.75	15.78	18.25	2.94	Up	7.00E-04
Cer(d62:3+hOEO)	971.9	15.46	15.36	9.02	0.94	2.63	14.75	24.69	18.25	-1.01	Down	1.85E-01
Cer(d62:7)	917.9	22.52	15.36	9.63	0.94	2.63	14.75	28.88	18.25	-1.47	Down	1.06E-02
Cer(d62:8)	915.8	31.16	32.27	0.55	1.02	30.35	30.22	31.93	34.30	1.04	Up	4.30E-03
Cer(d63:2EO)	971.9	14.56	15.36	9.70	0.94	2.63	14.75	25.42	18.25	1.05	Up	6.89E-01
Cer(d63:3+pO)	955.9	4.29	15.36	4.03	0.94	2.63	14.75	16.03	18.25	3.58	Up	6.00E-04
Cer(d63:3EO)	969.9	13.27	15.36	10.38	0.94	2.63	14.75	25.94	18.25	1.16	Up	8.29E-01
Cer(d63:4EO)	967.9	18.35	15.36	6.50	0.94	2.63	14.75	25.39	18.25	-1.19	Down	1.56E-02
Cer(d64:1EO)	988	11.97	15.36	6.88	0.94	2.63	14.75	20.66	18.25	1.28	Up	5.18E-01
Cer(d64:3+hOEO)	999.9	8.75	15.36	6.20	0.94	2.63	14.75	16.26	18.25	1.76	Up	2.07E-02
Cer(d65:0)	974	9.29	15.36	7.13	0.94	2.63	14.75	17.64	18.25	1.65	Up	2.05E-01
Cer(d66:1EO)	1016	8.54	15.36	6.50	0.94	2.63	14.75	19.91	18.25	1.8	Up	3.87E-02
Cer(d68:1EO)	1044	8.55	15.36	7.65	0.94	2.63	14.75	22.11	18.25	1.8	Up	4.44E-02
Cer(d72:3EO)	1096	9.76	15.36	9.03	0.94	2.63	14.75	23.01	18.25	1.57	Up	2.79E-01
Cer(d73:0+pOEO)	1132	12.33	15.36	9.88	0.94	2.63	14.75	27.93	18.25	1.25	Up	7.34E-01
Cer(d77:0+pOEO)	1188	14.40	15.36	7.38	0.94	2.63	14.75	25.16	18.25	1.07	Up	4.87E-01
Cer(d78:0EO)	1186	9.57	15.36	7.31	0.94	2.63	14.75	19.27	18.25	1.61	Up	6.87E-02

CerG1(d14:0/44:1EO)	1066	22.02	15.36	2.95	0.94	17.21	14.75	27.63	18.25	-1.43	Down	1.00E-04
CerG1(d14:0/45:1EO)	1080	18.38	15.36	5.78	0.94	4.21	14.75	24.91	18.25	-1.2	Down	6.80E-03
CerG1(d16:0+pO/18:1)	715.6	19.13	22.20	3.61	2.75	13.26	14.95	22.78	24.08	1.16	Up	2.30E-03
CerG1(d16:0+pO/22:0+O)	789.6	27.24	15.36	0.58	0.94	25.86	14.75	28.07	18.25	-1.77	Down	1.00E-04
CerG1(d16:0+pO/22:1)	771.6	20.26	15.36	9.75	0.94	2.63	14.75	27.17	18.25	-1.32	Down	2.10E-02
CerG1(d16:0+pO/23:1)	785.6	20.63	25.60	8.25	2.75	2.63	18.25	26.55	27.38	1.24	Up	4.39E-02
CerG1(d16:1/18:1)	697.5	17.91	22.04	5.93	2.70	2.63	14.95	22.35	24.32	1.23	Up	2.30E-03
CerG1(d16:1/23:1)	767.6	20.69	24.91	8.30	2.48	2.63	18.25	26.60	27.16	1.2	Up	6.44E-01
CerG1(d16:1/44:0EO)	1094	24.41	25.36	1.02	2.45	23.53	18.25	26.23	27.26	1.04	Up	1.29E-02
CerG1(d16:1/44:1EO)	1092	25.19	25.56	1.09	2.62	23.75	18.25	27.34	27.88	1.01	Up	1.90E-01
CerG1(d16:1/44:2EO)	1090	24.27	15.36	1.04	0.94	22.92	14.75	26.41	18.25	-1.58	Down	1.00E-04
CerG1(d17:0/44:1EO)	1108	15.63	15.36	6.79	0.94	2.63	14.75	22.77	18.25	-1.02	Down	4.49E-01
CerG1(d17:0/44:2EO)	1106	12.93	15.36	9.71	0.94	2.63	14.75	25.05	18.25	1.19	Up	8.29E-01
CerG1(d17:0+pO)	479.3	26.86	27.90	0.97	1.34	25.85	25.58	28.81	29.67	1.04	Up	6.88E-02
CerG1(d17:1/18:0)	713.6	16.14	22.75	8.72	1.51	2.63	18.25	22.76	24.46	1.41	Up	6.00E-04
CerG1(d17:1/44:2EO)	1104	14.95	15.36	8.38	0.94	2.63	14.75	23.81	18.25	1.03	Up	1.31E-01
CerG1(d18:0/18:0)	729.6	25.50	26.34	1.27	1.29	22.38	23.56	27.42	28.27	1.03	Up	1.30E-01
CerG1(d18:0/22:0)	785.7	22.25	15.36	1.98	0.94	20.48	14.75	26.55	18.25	-1.45	Down	1.00E-04
CerG1(d18:0/23:0)	799.7	25.35	25.52	0.56	2.79	24.25	18.25	26.16	27.38	1.01	Up	7.93E-02
CerG1(d18:0/24:0)	813.7	28.04	26.58	0.39	3.21	27.51	18.25	28.75	28.87	-1.05	Down	6.01E-01
CerG1(d18:0/24:0+O)	829.7	28.09	28.60	0.41	1.54	27.58	25.42	28.90	30.15	1.02	Up	2.88E-01
CerG1(d18:0/25:0)	827.7	25.42	25.22	0.93	2.66	23.54	18.25	26.82	27.76	-1.01	Down	3.72E-01
CerG1(d18:0/25:0+O)	843.7	28.24	29.17	1.30	2.22	25.84	25.45	30.06	32.97	1.03	Up	2.41E-01
CerG1(d18:0/26:0+O)	857.7	17.52	15.36	7.13	0.94	2.63	14.75	25.07	18.25	-1.14	Down	1.29E-02
CerG1(d18:0+pO/16:0)	717.6	16.55	25.26	11.04	0.75	2.63	23.83	25.12	25.99	1.53	Up	1.80E-03
CerG1(d18:0+pO/18:0)	745.6	23.76	28.80	7.85	1.52	12.31	26.43	30.08	30.60	1.21	Up	2.68E-01
CerG1(d18:0+pO/18:1)	743.6	26.92	26.44	1.74	2.93	24.69	18.25	29.72	29.27	-1.02	Down	6.51E-01
CerG1(d18:0+pO/20:0)	773.6	21.05	26.58	9.00	1.43	2.63	23.09	27.60	28.04	1.26	Up	2.07E-01
CerG1(d18:0+pO/20:3)	767.6	27.29	27.92	0.53	0.85	26.48	26.66	28.46	29.02	1.02	Up	4.85E-02
CerG1(d18:0+pO/22:0)	801.7	27.40	15.36	0.71	0.94	26.13	14.75	28.71	18.25	-1.78	Down	1.00E-04
CerG1(d18:0+pO/22:1)	799.7	23.51	22.98	4.66	3.14	18.12	15.58	29.46	27.00	-1.02	Down	8.05E-01
CerG1(d18:0+pO/23:0)	815.7	28.13	15.36	0.33	0.94	27.43	14.75	28.67	18.25	-1.83	Down	1.00E-04
CerG1(d18:0+pO/23:1)	813.7	22.75	23.25	1.46	1.93	19.45	18.25	25.39	25.30	1.02	Up	1.86E-01
CerG1(d18:0+pO/23:2)	811.7	16.38	25.37	8.98	3.42	2.63	14.95	27.43	27.31	1.55	Up	2.25E-02
CerG1(d18:0+pO/24:0+O)	845.7	28.86	30.54	3.82	1.68	21.50	27.90	32.14	32.62	1.06	Up	3.56E-01
CerG1(d18:0+pO/24:1)	827.7	31.55	25.87	0.43	1.69	30.82	23.10	32.55	28.23	-1.22	Down	1.00E-04
CerG1(d18:0+pO/24:2)	825.7	31.50	26.26	0.53	1.56	30.88	23.03	32.83	28.25	-1.2	Down	1.00E-04
CerG1(d18:0+pO/25:1)	841.7	30.34	30.84	0.94	2.18	27.81	27.23	31.55	33.13	1.02	Up	7.35E-01

CerG1(d18:0+pO/25:2)	839.7	29.84	30.87	0.51	1.82	29.38	28.41	31.03	33.29	1.03	Up	8.32E-02
CerG1(d18:0+pO/26:0)	857.7	22.13	25.54	7.33	1.64	2.63	21.68	27.11	27.84	1.15	Up	5.80E-01
CerG1(d18:0+pO/26:0+O)	873.7	26.29	28.71	1.13	1.87	25.37	25.06	28.97	31.66	1.09	Up	5.10E-03
CerG1(d18:0+pO/26:1)	855.7	29.99	29.64	1.20	2.02	26.73	25.66	31.62	32.20	-1.01	Down	1.00E+00
CerG1(d18:0+pO/26:2)	853.7	30.42	24.51	0.60	1.53	29.16	22.09	31.66	28.30	-1.24	Down	1.00E-04
CerG1(d18:0+pO/26:3)	851.7	27.35	28.24	1.11	1.00	24.63	26.29	28.92	29.79	1.03	Up	9.65E-02
CerG1(d18:0+pO/26:4)	849.7	24.05	27.68	4.57	1.41	16.70	25.45	28.59	29.58	1.15	Up	3.74E-02
CerG1(d18:0+pO/27:2)	867.7	26.43	27.81	1.19	1.60	23.26	24.48	27.49	31.10	1.05	Up	1.16E-02
CerG1(d18:0+pO/27:3)	865.7	25.91	25.55	0.81	2.68	23.95	18.25	27.08	27.90	-1.01	Down	5.66E-01
CerG1(d18:0+pO/27:4)	863.7	23.82	15.36	4.07	0.94	16.14	14.75	27.42	18.25	-1.55	Down	1.00E-04
CerG1(d18:0+pO/28:2)	881.7	24.58	24.81	1.29	2.50	21.08	18.25	26.15	26.77	1.01	Up	9.08E-02
CerG1(d18:0+pO/28:4)	877.7	27.25	27.34	0.58	1.36	26.34	24.44	28.27	28.87	1	Up	8.31E-01
CerG1(d18:0+pO/34:2)	965.8	12.04	20.94	8.94	2.94	2.63	14.95	23.21	23.24	1.74	Up	8.70E-03
CerG1(d18:0+pO/40:2)	1050	17.74	15.36	5.49	0.94	4.21	14.75	23.91	18.25	-1.15	Down	1.56E-02
CerG1(d18:0+pO/40:3)	1048	19.69	24.79	3.09	1.15	15.48	23.24	24.44	27.25	1.26	Up	2.00E-04
CerG1(d18:1/16:0)	699.6	25.93	22.12	1.83	2.66	22.49	15.58	28.13	24.82	-1.17	Down	1.10E-03
CerG1(d18:1/18:0)	727.6	28.95	29.75	1.07	1.55	26.29	27.61	30.25	31.58	1.03	Up	4.23E-01
CerG1(d18:1/19:0)	741.6	17.88	22.45	8.56	3.08	2.63	14.95	24.40	25.53	1.26	Up	1.86E-01
CerG1(d18:1/20:0)	755.6	26.56	27.68	0.84	1.29	25.02	24.58	27.52	29.30	1.04	Up	2.37E-02
CerG1(d18:1/20:0+O)	771.6	22.52	15.36	6.35	0.94	5.89	14.75	28.95	18.25	-1.47	Down	4.00E-04
CerG1(d18:1/20:1)	753.6	19.74	15.36	9.40	0.94	2.63	14.75	27.17	18.25	-1.29	Down	2.10E-02
CerG1(d18:1/20:3)	749.6	24.88	27.08	4.05	0.86	17.72	25.80	28.76	28.22	1.09	Up	6.22E-01
CerG1(d18:1/22:0)	783.7	27.36	28.20	0.72	0.96	26.12	26.56	28.67	29.32	1.03	Up	2.85E-02
CerG1(d18:1/22:0+O)	799.7	28.11	30.08	3.38	1.30	21.68	27.99	31.37	31.66	1.07	Up	3.47E-01
CerG1(d18:1/22:1)	781.6	29.07	29.61	0.44	1.69	28.18	26.54	29.82	31.55	1.02	Up	3.10E-01
CerG1(d18:1/22:2)	779.6	21.27	15.36	7.63	0.94	2.63	14.75	27.26	18.25	-1.38	Down	1.50E-02
CerG1(d18:1/23:0)	797.7	30.95	28.58	0.45	1.50	30.42	24.80	31.91	30.15	-1.08	Down	2.00E-04
CerG1(d18:1/23:0+O)	813.7	32.36	29.30	0.62	1.49	31.10	26.70	33.37	31.37	-1.1	Down	1.00E-04
CerG1(d18:1/23:1)	795.7	28.80	29.38	0.57	1.38	27.99	26.74	29.71	30.95	1.02	Up	1.99E-01
CerG1(d18:1/23:5)	787.6	22.94	15.36	1.40	0.94	20.17	14.75	25.69	18.25	-1.49	Down	1.00E-04
CerG1(d18:1/24:0)	811.7	30.22	30.06	0.33	1.49	29.47	26.91	30.60	31.68	-1.01	Down	7.13E-01
CerG1(d18:1/24:1)	809.7	31.55	25.93	0.53	1.49	30.51	23.77	32.70	29.10	-1.22	Down	1.00E-04
CerG1(d18:1/24:2)	807.7	29.68	30.07	0.92	1.29	28.32	28.62	30.92	33.23	1.01	Up	4.08E-01
CerG1(d18:1/25:0)	825.7	28.73	27.57	0.86	3.39	27.28	18.25	30.54	30.74	-1.04	Down	5.80E-01
CerG1(d18:1/25:1)	823.7	30.41	30.31	0.71	2.15	28.72	26.76	31.58	32.70	-1	Down	8.72E-01
CerG1(d18:1/25:2)	821.7	21.90	26.54	5.40	1.72	16.86	22.20	29.36	27.79	1.21	Up	1.34E-01
CerG1(d18:1/26:0)	839.7	26.62	26.04	0.82	2.94	24.98	18.25	27.95	28.71	-1.02	Down	4.98E-01
CerG1(d18:1/26:1)	837.7	30.18	29.68	0.96	2.18	27.60	25.66	31.26	32.35	-1.02	Down	9.76E-01

CerG1(d18:1/26:2)	835.7	27.84	27.47	1.26	2.02	26.58	22.22	30.01	29.64	-1.01	Down	8.33E-01
CerG1(d18:1/27:1)	851.7	24.70	23.84	1.83	3.66	19.69	14.95	27.10	27.18	-1.04	Down	1.00E+00
CerG1(d18:1/28:4)	859.7	26.13	26.47	1.98	1.29	20.68	23.50	28.01	27.87	1.01	Up	8.80E-01
CerG1(d18:1/44:2EO)	1118	23.12	15.36	1.11	0.94	21.09	14.75	25.28	18.25	-1.51	Down	1.00E-04
CerG1(d18:1+hO)	491.3	19.20	15.36	2.05	0.94	14.78	14.75	23.17	18.25	-1.25	Down	1.00E-03
CerG1(d18:1+hO/20:3)	765.6	25.31	24.13	1.10	2.23	24.17	18.25	27.30	26.37	-1.05	Down	3.40E-01
CerG1(d18:1+hO/22:0+O)	815.6	26.70	15.36	1.14	0.94	23.57	14.75	27.66	18.25	-1.74	Down	1.00E-04
CerG1(d18:1+hO/24:2)	823.7	27.57	15.36	0.82	0.94	26.19	14.75	29.24	18.25	-1.79	Down	1.00E-04
CerG1(d18:1+hO/25:0+O)	857.7	25.89	15.36	5.34	0.94	16.22	14.75	30.58	18.25	-1.69	Down	1.00E-04
CerG1(d18:1+hO/25:2)	837.7	24.21	25.03	2.59	2.69	18.03	18.25	26.77	27.84	1.03	Up	2.88E-01
CerG1(d18:2/18:0)	725.6	27.05	15.36	1.61	0.94	25.52	14.75	29.72	18.25	-1.76	Down	1.00E-04
CerG1(d18:2/18:0+O)	741.6	24.87	24.35	1.58	2.30	22.91	18.25	27.38	26.49	-1.02	Down	8.33E-01
CerG1(d18:2/18:1)	723.6	23.64	24.38	1.16	0.99	20.78	22.62	24.97	25.81	1.03	Up	1.11E-01
CerG1(d18:2/20:0)	753.6	19.41	26.48	7.16	1.48	5.89	22.60	28.67	27.97	1.36	Up	6.04E-02
CerG1(d18:2/20:0+O)	769.6	24.54	15.36	3.11	0.94	21.40	14.75	29.34	18.25	-1.6	Down	1.00E-04
CerG1(d18:2/22:0+O)	797.6	27.21	15.36	0.98	0.94	26.07	14.75	28.62	18.25	-1.77	Down	1.00E-04
CerG1(d18:2/23:0+O)	811.7	25.97	15.36	3.93	0.94	19.95	14.75	29.80	18.25	-1.69	Down	1.00E-04
CerG1(d18:2/25:1)	821.7	23.18	15.36	5.12	0.94	15.46	14.75	27.35	18.25	-1.51	Down	1.00E-04
CerG1(d19:0+pO)	507.3	22.93	24.70	3.03	2.34	18.28	18.25	27.41	27.16	1.08	Up	1.51E-01
CerG1(d19:1+hO)	505.3	25.36	21.32	0.85	1.18	24.48	18.25	27.00	23.06	-1.19	Down	1.00E-04
CerG1(d20:1)	503.3	15.23	21.73	6.74	2.51	2.63	15.58	21.76	24.59	1.43	Up	1.70E-03
CerG1(d21:0)	519.4	18.19	15.36	2.91	0.94	13.37	14.75	23.94	18.25	-1.18	Down	3.50E-03
CerG1(d21:0+pO)	535.4	19.10	23.80	2.34	2.25	15.46	21.35	23.52	29.60	1.25	Up	1.00E-04
CerG1(d22:0+pO)	549.4	17.02	24.26	7.53	1.67	2.63	22.46	23.40	27.56	1.43	Up	2.00E-04
CerG1(d22:1)	531.4	21.90	24.96	1.27	1.75	20.23	23.00	24.29	28.35	1.14	Up	1.00E-04
CerG1(d23:0+pO)	563.4	18.87	24.54	3.16	1.90	14.25	22.77	24.27	29.58	1.3	Up	1.00E-04
CerG1(d23:1)	545.4	19.17	24.54	2.77	1.94	15.16	22.76	24.28	29.67	1.28	Up	1.00E-04
CerG1(d23:1+hO)	561.4	14.50	22.98	8.01	1.46	2.63	21.33	23.81	25.34	1.58	Up	5.60E-03
CerG1(d24:0+pO/34:3)	1048	19.66	15.36	7.04	0.94	2.63	14.75	24.80	18.25	-1.28	Down	7.92E-02
CerG1(d24:0+pO/36:3)	1076	19.76	15.36	3.74	0.94	15.61	14.75	25.82	18.25	-1.29	Down	1.00E-04
CerG1(d24:0+pO/36:4)	1074	17.61	15.36	5.68	0.94	5.89	14.75	25.16	18.25	-1.15	Down	3.16E-01
CerG1(d24:1+hO)	575.4	21.54	24.84	1.07	1.60	19.88	22.75	23.53	27.65	1.15	Up	1.00E-04
CerG1(d25:0)	575.4	20.21	23.05	5.28	2.14	5.89	18.25	25.36	26.26	1.14	Up	1.18E-01
CerG1(d25:1+hO)	589.4	18.75	24.70	3.51	1.95	13.09	22.75	24.32	29.73	1.32	Up	1.00E-04
CerG1(d26:0+pO)	605.5	15.21	15.36	6.58	0.94	2.63	14.75	20.58	18.25	1.01	Up	9.05E-02
CerG1(d26:1)	587.4	16.55	15.36	5.33	0.94	2.63	14.75	20.74	18.25	-1.08	Down	7.92E-02
CerG1(d27:0)	603.5	21.16	22.16	2.45	2.93	17.24	15.58	24.92	25.90	1.05	Up	3.85E-01
CerG1(d27:0+O)	619.5	14.62	15.36	7.75	0.94	2.63	14.75	21.42	18.25	1.05	Up	7.91E-02

CerG1(d27:1)	601.5	18.89	22.98	2.48	1.60	14.28	18.25	21.70	24.54	1.22	Up	1.00E-04
CerG1(d28:0+2O)	649.5	16.73	15.36	7.52	0.94	2.63	14.75	22.81	18.25	-1.09	Down	7.93E-02
CerG1(d28:1+pO+2O)	663.5	21.62	25.69	0.87	1.85	20.94	23.16	23.72	30.04	1.19	Up	1.00E-04
CerG1(d28:2)	613.5	13.89	22.60	7.61	2.92	2.63	14.86	23.68	25.45	1.63	Up	1.20E-03
CerG1(d29:0+2O)	663.5	16.88	15.36	5.01	0.94	2.63	14.75	21.60	18.25	-1.1	Down	1.70E-03
CerG1(d29:1+2O)	661.5	13.03	20.53	6.83	3.38	2.63	15.00	20.58	25.15	1.57	Up	4.60E-03
CerG1(d29:1+pO+2O)	677.5	19.90	24.52	1.91	1.98	16.55	22.77	24.00	29.76	1.23	Up	1.00E-04
CerG1(d30:0+O)	661.5	15.22	22.84	6.43	2.43	4.21	18.25	25.11	26.84	1.5	Up	2.80E-03
CerG1(d30:0+pO+2O)	693.5	11.18	19.48	9.47	3.39	2.63	15.00	23.85	23.01	1.74	Up	8.90E-02
CerG1(d30:0+pO+O)	677.5	8.55	15.36	9.35	0.94	2.63	14.75	26.27	18.25	1.8	Up	6.25E-02
CerG1(d30:1)	643.5	19.46	23.34	6.14	2.78	4.21	18.25	25.73	27.89	1.2	Up	1.10E-01
CerG1(d30:1+hO+O)	675.5	14.66	19.52	7.02	4.08	2.63	15.07	20.72	25.03	1.33	Up	1.10E-01
CerG1(d30:2)	641.5	17.59	24.48	6.03	2.19	2.63	21.57	25.86	27.74	1.39	Up	3.60E-03
CerG1(d30:3)	639.5	13.04	19.01	8.51	3.80	2.63	14.75	23.91	24.35	1.46	Up	1.85E-01
CerG1(d31:0+O)	675.5	15.47	22.46	6.99	3.88	2.63	15.07	23.08	26.87	1.45	Up	7.40E-03
CerG1(d31:0+pO+2O)	707.5	26.84	28.02	0.63	3.15	26.15	18.25	27.89	30.58	1.04	Up	8.00E-04
CerG1(d31:1)	657.5	18.08	22.45	6.22	3.88	2.63	15.07	24.86	26.85	1.24	Up	3.74E-02
CerG1(d31:1+hO+O)	689.5	29.81	31.57	0.81	1.63	28.81	27.52	30.88	33.04	1.06	Up	5.60E-03
CerG1(d31:1+pO+2O)	705.5	15.83	25.01	8.60	2.18	2.63	23.05	22.49	31.10	1.58	Up	1.00E-04
CerG1(d31:2+2O)	687.5	20.48	24.97	1.48	2.09	17.76	23.05	23.04	30.63	1.22	Up	1.00E-04
CerG1(d31:2+hO)	671.5	16.15	15.36	1.47	0.94	13.46	14.75	18.10	18.25	-1.05	Down	1.51E-01
CerG1(d32:0+pO+2O)	721.5	19.26	15.36	6.56	0.94	2.63	14.75	24.96	18.25	-1.25	Down	4.50E-03
CerG1(d32:1)	671.5	6.98	15.36	5.29	0.94	2.63	14.75	14.97	18.25	2.2	Up	1.00E-04
CerG1(d32:1+O)	687.5	20.18	15.36	1.16	0.94	17.97	14.75	21.76	18.25	-1.31	Down	1.00E-04
CerG1(d32:2)	669.5	19.53	25.58	4.56	2.39	12.91	22.46	25.90	28.85	1.31	Up	1.40E-03
CerG1(d32:2+2O)	701.5	18.52	15.36	1.65	0.94	16.50	14.75	21.35	18.25	-1.21	Down	2.00E-04
CerG1(d32:2+hO+2O)	717.5	25.48	21.37	2.18	5.09	22.51	15.11	29.80	26.97	-1.19	Down	1.04E-01
CerG1(d32:2+pO)	685.5	25.29	30.37	6.64	1.24	15.79	27.89	31.30	32.08	1.2	Up	1.63E-02
CerG1(d32:3)	667.5	18.86	23.91	3.75	2.78	14.10	18.25	25.82	27.81	1.27	Up	1.40E-03
CerG1(d32:4+pO)	681.5	15.35	15.36	10.06	0.94	2.63	14.75	28.77	18.25	1	Up	3.72E-01
CerG1(d33:0)	687.6	16.72	22.79	2.77	1.99	12.72	21.14	23.15	27.14	1.36	Up	4.00E-04
CerG1(d33:0+pO)	703.6	23.67	27.16	0.93	2.30	22.41	23.59	25.23	32.97	1.15	Up	2.00E-04
CerG1(d33:0+pO+2O)	735.5	28.10	28.51	0.89	3.43	26.23	18.25	29.30	31.91	1.01	Up	2.68E-02
CerG1(d33:0+pO+O)	719.6	24.26	21.25	3.81	1.20	17.33	18.25	27.31	23.29	-1.14	Down	6.04E-02
CerG1(d33:1+hO+O)	717.5	31.95	15.36	0.61	0.94	31.06	14.75	33.04	18.25	-2.08	Down	1.00E-04
CerG1(d33:1+pO+2O)	733.5	25.68	26.86	0.99	0.93	23.77	24.65	27.26	28.27	1.05	Up	7.40E-03
CerG1(d33:2+2O)	715.5	31.21	32.83	0.75	1.71	30.27	28.71	32.40	34.17	1.05	Up	1.06E-02
CerG1(d33:4)	679.5	15.39	15.36	10.12	0.94	2.63	14.75	23.47	18.25	-1	Down	2.80E-01

CerG1(d34:0)	701.6	21.42	23.67	1.79	1.90	18.05	18.25	22.88	25.82	1.11	Up	2.00E-04
CerG1(d34:2)	697.5	18.61	24.53	3.91	2.69	12.65	21.93	24.66	29.20	1.32	Up	1.00E-04
CerG1(d34:2+2O)	729.5	29.17	30.01	0.71	1.48	28.41	26.24	30.45	31.42	1.03	Up	2.88E-02
CerG1(d34:3+2O)	727.5	23.81	15.36	6.39	0.94	13.92	14.75	29.47	18.25	-1.55	Down	2.80E-03
CerG1(d35:0)	715.6	18.85	22.11	2.73	2.85	15.36	15.58	25.09	26.55	1.17	Up	1.08E-02
CerG1(d35:1)	713.6	17.05	21.20	5.06	4.86	2.63	14.86	20.52	27.86	1.24	Up	1.18E-01
CerG1(d35:1+O)	729.6	23.94	21.41	5.42	4.25	19.31	15.15	30.82	26.19	-1.12	Down	5.66E-01
CerG1(d35:2)	711.6	28.56	21.61	2.68	2.46	23.21	15.07	31.78	25.33	-1.32	Down	1.00E-04
CerG1(d35:2+2O)	743.6	34.40	34.88	0.81	1.46	33.70	31.51	36.45	36.45	1.01	Up	6.42E-02
CerG1(d35:2+hO)	727.6	20.64	15.36	7.03	0.94	2.63	14.75	29.58	18.25	-1.34	Down	4.00E-04
CerG1(d35:3+2O)	741.5	31.57	33.15	0.65	1.76	30.68	28.20	32.55	34.38	1.05	Up	2.50E-03
CerG1(d35:4)	707.5	27.74	28.49	1.14	3.29	25.52	18.25	29.38	30.30	1.03	Up	9.70E-03
CerG1(d36:0+pO)	745.6	22.50	24.11	1.07	2.18	21.51	18.25	25.03	26.71	1.07	Up	8.90E-03
CerG1(d36:0+pO+2O)	777.6	24.90	22.26	7.93	2.23	17.97	18.25	35.78	27.58	-1.12	Down	4.42E-01
CerG1(d36:1)	727.6	20.84	23.96	2.74	2.13	15.61	18.25	25.46	26.71	1.15	Up	8.60E-03
CerG1(d36:1+hO+O)	759.6	24.59	23.76	1.61	2.05	22.37	18.25	27.33	25.47	-1.03	Down	6.01E-01
CerG1(d36:2)	725.6	22.78	22.19	1.83	3.80	18.85	15.07	24.67	27.77	-1.03	Down	6.32E-01
CerG1(d36:2+2O)	757.6	28.12	15.36	1.61	0.94	24.51	14.75	29.94	18.25	-1.83	Down	1.00E-04
CerG1(d36:3)	723.6	17.83	15.36	8.74	0.94	2.63	14.75	26.62	18.25	-1.16	Down	1.40E-01
CerG1(d36:3+2O)	755.6	20.33	23.18	2.77	3.68	15.88	15.58	24.87	28.05	1.14	Up	5.00E-02
CerG1(d36:3+O)	739.6	23.92	24.57	1.48	0.50	22.73	23.85	26.66	25.49	1.03	Up	7.93E-02
CerG1(d36:4)	721.5	23.85	24.48	1.54	0.59	22.01	23.40	26.65	25.47	1.03	Up	9.08E-02
CerG1(d36:4+pO)	737.5	18.02	21.74	5.31	2.53	2.63	14.95	21.70	23.64	1.21	Up	3.40E-03
CerG1(d37:0)	743.6	20.05	25.10	2.61	2.29	15.67	22.33	25.23	29.40	1.25	Up	1.00E-04
CerG1(d37:0+O)	759.6	21.20	23.31	2.32	3.59	15.22	15.07	23.85	27.85	1.1	Up	9.08E-02
CerG1(d37:1)	741.6	19.60	23.05	2.32	3.54	13.60	15.07	22.22	28.08	1.18	Up	1.70E-03
CerG1(d37:1+O)	757.6	23.98	15.36	3.22	0.94	20.95	14.75	29.14	18.25	-1.56	Down	1.00E-04
CerG1(d37:2+hO+O)	771.6	33.28	34.11	0.64	1.60	32.30	30.77	34.76	35.58	1.03	Up	4.87E-02
CerG1(d37:2+pO)	755.6	20.10	15.36	11.57	0.94	2.63	14.75	28.63	18.25	-1.31	Down	6.92E-02
CerG1(d37:3)	737.6	22.59	24.90	4.12	2.26	16.36	22.85	28.97	29.72	1.1	Up	1.18E-01
CerG1(d37:3+pO)	753.6	23.34	15.36	9.52	0.94	2.63	14.75	30.75	18.25	-1.52	Down	5.95E-02
CerG1(d37:4)	735.6	23.33	15.36	9.52	0.94	2.63	14.75	30.75	18.25	-1.52	Down	5.95E-02
CerG1(d38:0+O)	773.6	23.23	24.03	1.39	2.73	20.03	21.06	24.96	28.77	1.03	Up	3.82E-01
CerG1(d38:1)	755.6	18.85	23.46	2.38	2.40	12.76	20.92	21.84	27.58	1.24	Up	1.00E-04
CerG1(d38:2)	753.6	21.01	26.34	5.91	1.67	11.99	23.77	26.05	28.85	1.25	Up	4.50E-03
CerG1(d38:2+hO+O)	785.6	27.07	27.94	1.01	0.86	24.35	26.73	28.47	29.20	1.03	Up	6.48E-02
CerG1(d38:2+pO+2O)	801.6	26.01	25.05	2.19	2.54	20.41	18.25	29.08	27.10	-1.04	Down	3.25E-01
CerG1(d38:3)	751.6	18.04	25.14	9.11	1.58	2.63	22.82	25.35	27.74	1.39	Up	1.78E-02

CerG1(d38:3+2O)	783.6	26.74	25.84	1.33	2.81	25.58	18.25	29.46	28.11	-1.03	Down	6.89E-01
CerG1(d38:4)	749.6	17.67	15.36	8.87	0.94	2.63	14.75	27.41	18.25	-1.15	Down	1.03E-01
CerG1(d38:4+2O)	781.6	21.87	15.36	1.70	0.94	18.10	14.75	23.40	18.25	-1.42	Down	1.00E-04
CerG1(d38:4+pO)	765.6	20.60	15.36	3.12	0.94	17.04	14.75	25.51	18.25	-1.34	Down	1.00E-04
CerG1(d38:5)	747.6	24.68	23.96	1.63	2.17	22.73	18.25	27.91	26.24	-1.03	Down	8.80E-01
CerG1(d38:5+pO)	763.6	25.54	22.48	4.41	2.79	19.19	15.58	29.34	24.48	-1.14	Down	1.17E-01
CerG1(d39:0)	771.7	17.84	15.36	2.43	0.94	15.34	14.75	23.58	18.25	-1.16	Down	4.00E-04
CerG1(d39:0+O)	787.7	24.07	25.79	1.72	3.79	19.87	21.28	25.49	31.58	1.07	Up	6.95E-01
CerG1(d39:0+pO)	787.7	20.82	23.43	1.67	2.69	17.88	20.90	23.29	28.97	1.13	Up	1.88E-02
CerG1(d39:1)	769.6	24.83	25.41	2.45	3.51	19.47	21.36	28.67	30.51	1.02	Up	6.55E-01
CerG1(d39:1+hO+O)	801.6	18.62	21.47	5.41	2.35	2.63	15.58	21.76	23.94	1.15	Up	7.00E-03
CerG1(d39:3+pO)	781.6	23.85	24.80	5.68	1.69	14.68	21.78	28.88	27.13	1.04	Up	2.88E-01
CerG1(d39:4+hO)	779.6	23.32	15.36	7.10	0.94	15.45	14.75	32.35	18.25	-1.52	Down	2.00E-04
CerG1(d39:4+pO)	779.6	28.32	32.53	5.62	1.61	18.15	28.92	33.80	34.45	1.15	Up	2.89E-02
CerG1(d39:5)	761.6	27.69	32.41	6.24	1.60	18.21	28.87	33.70	34.30	1.17	Up	2.27E-02
CerG1(d39:5+pO)	777.6	27.40	15.36	3.96	0.94	22.19	14.75	34.17	18.25	-1.78	Down	1.00E-04
CerG1(d40:0+O)	801.7	24.13	24.14	1.60	3.97	19.88	15.07	25.91	29.96	1	Up	8.33E-01
CerG1(d40:0+pO)	801.7	21.21	24.30	2.38	3.41	14.97	20.91	24.48	30.04	1.15	Up	2.25E-02
CerG1(d40:1)	783.7	23.18	24.42	2.09	4.42	18.15	15.07	26.08	30.65	1.05	Up	3.98E-01
CerG1(d40:1+O)	799.7	22.74	23.86	1.37	2.57	19.79	21.06	24.84	27.91	1.05	Up	2.15E-01
CerG1(d40:2)	781.6	25.24	24.04	3.87	3.09	19.64	20.95	28.41	29.42	-1.05	Down	7.35E-01
CerG1(d40:3)	779.6	27.51	27.40	1.04	2.38	25.25	23.86	28.57	30.25	-1	Down	7.82E-01
CerG1(d40:3+2O)	811.6	24.83	25.21	2.46	2.56	18.68	18.25	26.70	27.05	1.02	Up	4.06E-01
CerG1(d40:4)	777.6	24.51	15.36	0.89	0.94	22.13	14.75	25.40	18.25	-1.6	Down	1.00E-04
CerG1(d40:4+pO)	793.6	24.81	25.23	2.44	2.58	18.68	18.25	26.66	27.08	1.02	Up	3.89E-01
CerG1(d41:0)	799.7	30.16	30.07	1.06	1.90	27.59	26.16	31.74	32.35	-1	Down	9.02E-01
CerG1(d41:0+O)	815.7	22.24	24.44	3.19	2.24	15.75	18.25	25.28	27.19	1.1	Up	6.48E-02
CerG1(d41:0+pO+2O)	847.7	14.52	15.36	9.45	0.94	2.63	14.75	22.86	18.25	1.06	Up	2.80E-01
CerG1(d41:0+pO+O)	831.7	23.92	15.36	3.23	0.94	17.93	14.75	28.11	18.25	-1.56	Down	1.00E-04
CerG1(d41:1)	797.7	17.83	24.56	3.51	2.00	11.70	21.65	22.83	28.67	1.38	Up	1.00E-04
CerG1(d41:1+O)	813.7	18.04	22.77	1.99	3.51	13.03	14.97	19.84	28.18	1.26	Up	2.00E-04
CerG1(d41:3)	793.6	23.66	15.36	3.74	0.94	17.72	14.75	26.65	18.25	-1.54	Down	1.00E-04
CerG1(d41:4+pO)	807.6	31.24	31.98	0.52	1.80	30.25	27.86	32.35	33.80	1.02	Up	1.94E-01
CerG1(d41:5)	789.6	27.38	15.36	5.57	0.94	19.14	14.75	32.40	18.25	-1.78	Down	1.00E-04
CerG1(d41:5+hO)	805.6	30.76	31.82	0.61	1.65	30.22	27.94	31.93	33.46	1.03	Up	3.36E-02
CerG1(d41:6)	787.6	26.82	22.27	5.50	2.65	15.20	15.59	31.74	24.25	-1.2	Down	6.94E-02
CerG1(d42:0+pO+2O)	861.7	23.26	15.36	5.82	0.94	14.19	14.75	27.65	18.25	-1.51	Down	1.50E-02
CerG1(d42:0+pO+O)	845.7	22.94	15.36	1.73	0.94	19.27	14.75	24.46	18.25	-1.49	Down	1.00E-04

CerG1(d42:1+O)	827.7	19.34	22.33	2.02	4.04	14.68	14.97	22.04	27.52	1.15	Up	3.65E-02
CerG1(d42:2)	809.7	18.71	23.60	2.10	3.01	13.70	20.78	21.28	28.81	1.26	Up	1.00E-04
CerG1(d42:3)	807.7	25.12	28.08	4.68	3.44	18.30	18.25	29.40	31.10	1.12	Up	1.66E-01
CerG1(d42:4)	805.6	26.32	29.73	2.15	1.68	21.65	26.02	28.16	31.33	1.13	Up	1.50E-03
CerG1(d42:4+pO)	821.6	22.50	26.84	4.16	1.25	15.89	24.37	26.43	28.59	1.19	Up	5.00E-04
CerG1(d43:0)	827.7	28.90	23.11	4.00	4.12	23.15	14.86	32.62	28.16	-1.25	Down	1.06E-02
CerG1(d43:0+pO)	843.7	21.75	25.35	2.19	2.17	18.45	22.23	25.88	28.90	1.17	Up	7.00E-04
CerG1(d43:0+pO+2O)	875.7	25.65	26.30	1.52	3.45	22.71	18.25	27.10	30.12	1.03	Up	2.60E-01
CerG1(d43:0+pO+O)	859.7	26.48	15.36	6.07	0.94	16.28	14.75	31.06	18.25	-1.72	Down	1.00E-04
CerG1(d43:1)	825.7	23.33	29.95	9.37	1.54	2.63	27.46	30.51	31.78	1.28	Up	2.68E-02
CerG1(d43:1+hO+O)	857.7	20.35	29.39	7.98	1.89	2.63	26.48	26.08	31.71	1.44	Up	1.00E-04
CerG1(d43:2+2O)	855.7	29.09	26.64	1.00	5.11	26.89	14.75	30.74	30.95	-1.09	Down	4.06E-01
CerG1(d43:4+pO)	835.7	23.88	27.42	3.40	1.11	16.12	24.60	27.05	28.52	1.15	Up	5.00E-04
CerG1(d43:5+pO)	833.6	14.33	23.99	8.22	2.09	2.63	18.25	25.61	26.30	1.67	Up	1.90E-03
CerG1(d44:0)	841.7	28.14	25.32	1.04	3.72	25.30	18.25	28.87	30.60	-1.11	Down	6.88E-02
CerG1(d44:1+hO+O)	871.7	26.60	15.36	5.81	0.94	17.02	14.75	31.68	18.25	-1.73	Down	1.00E-04
CerG1(d44:3)	835.7	23.70	24.19	5.96	2.25	14.42	18.25	29.02	26.71	1.02	Up	2.88E-01
CerG1(d44:4)	833.7	28.00	28.26	1.72	1.00	25.12	26.29	29.88	29.79	1.01	Up	1.00E+00
CerG1(d44:4+pO)	849.7	21.97	15.36	3.28	0.94	19.11	14.75	27.65	18.25	-1.43	Down	1.00E-04
CerG1(d44:5)	831.7	24.81	15.36	2.93	0.94	19.64	14.75	28.20	18.25	-1.61	Down	1.00E-04
CerG1(d44:5+pO)	847.7	26.86	29.13	1.48	1.38	23.88	26.97	29.10	31.40	1.08	Up	1.10E-03
CerG1(d44:6+pO)	845.6	22.12	24.34	4.18	2.38	14.27	18.25	26.02	26.79	1.1	Up	2.68E-01
CerG1(d45:0)	855.8	29.32	27.00	3.58	3.72	25.14	21.70	33.37	33.29	-1.09	Down	8.48E-02
CerG1(d45:0+pO)	871.7	22.11	15.36	2.55	0.94	17.88	14.75	26.69	18.25	-1.44	Down	1.00E-04
CerG1(d45:0+pO+O)	887.7	23.14	15.36	0.95	0.94	21.25	14.75	24.70	18.25	-1.51	Down	1.00E-04
CerG1(d45:1)	853.7	30.18	29.13	1.66	2.75	28.04	23.67	32.30	31.71	-1.04	Down	3.56E-01
CerG1(d45:1+hO+O)	885.7	27.48	26.86	0.86	3.42	25.42	18.25	28.54	30.22	-1.02	Down	7.12E-01
CerG1(d45:2)	851.7	29.85	30.56	1.07	1.58	26.99	28.23	31.33	32.55	1.02	Up	7.93E-02
CerG1(d45:3)	849.7	21.43	27.25	8.60	1.86	2.63	24.39	27.97	29.94	1.27	Up	1.04E-01
CerG1(d45:4+pO)	863.7	18.04	26.58	7.93	1.25	2.63	24.27	26.95	28.31	1.47	Up	5.10E-03
CerG1(d45:5+pO)	861.7	22.73	26.42	3.66	1.34	15.20	23.88	26.13	28.40	1.16	Up	1.10E-03
CerG1(d46:0)	869.8	25.28	30.45	7.55	1.08	15.06	28.46	31.45	32.14	1.2	Up	2.81E-01
CerG1(d46:0+pO+O)	901.8	13.55	15.36	9.12	0.94	2.63	14.75	23.73	18.25	1.13	Up	6.89E-01
CerG1(d46:1)	867.8	26.14	29.15	2.42	3.85	21.94	18.25	29.12	32.70	1.12	Up	7.00E-03
CerG1(d46:2)	865.7	23.85	15.36	7.16	0.94	2.63	14.75	28.81	18.25	-1.55	Down	4.00E-04
CerG1(d46:3+pO)	879.7	16.27	15.36	9.16	0.94	2.63	14.75	26.09	18.25	-1.06	Down	7.91E-02
CerG1(d46:4)	861.7	16.27	15.36	9.16	0.94	2.63	14.75	26.09	18.25	-1.06	Down	7.91E-02
CerG1(d46:5+pO)	875.7	26.52	28.10	1.27	1.45	23.83	26.13	28.81	30.25	1.06	Up	1.13E-02

CerG1(d47:0)	883.8	34.86	29.06	0.97	3.62	33.37	22.11	36.45	34.38	-1.2	Down	1.00E-04
CerG1(d47:0+O)	899.8	26.29	23.38	1.03	3.95	24.09	14.97	28.52	30.06	-1.12	Down	8.60E-03
CerG1(d47:1)	881.8	33.59	27.09	0.86	3.27	32.30	22.30	35.26	31.87	-1.24	Down	1.00E-04
CerG1(d47:1+hO+O)	913.8	22.16	24.95	1.85	1.48	19.19	21.76	25.47	26.60	1.13	Up	6.00E-04
CerG1(d47:1+O)	897.8	24.06	29.04	1.25	2.20	22.09	25.28	26.04	31.42	1.21	Up	1.00E-04
CerG1(d47:1+pO)	897.8	23.88	15.36	1.66	0.94	21.78	14.75	27.49	18.25	-1.55	Down	1.00E-04
CerG1(d47:2)	879.8	30.01	25.19	2.18	2.41	26.51	18.25	32.70	28.59	-1.19	Down	1.00E-04
CerG1(d47:3)	877.7	25.14	27.62	5.67	2.24	16.06	23.50	29.98	30.65	1.1	Up	7.12E-01
CerG1(d47:5+pO)	889.7	25.64	27.09	3.43	1.64	18.63	23.38	28.83	28.77	1.06	Up	5.80E-01
CerG1(d47:6)	871.7	24.44	26.95	4.61	1.76	16.42	23.06	29.22	28.72	1.1	Up	3.16E-01
CerG1(d47:6+pO)	887.7	21.89	26.12	2.42	1.51	18.47	23.30	25.23	28.22	1.19	Up	1.00E-04
CerG1(d48:1)	895.8	30.53	31.55	1.03	1.76	29.08	27.44	31.68	33.89	1.03	Up	5.24E-02
CerG1(d48:2)	893.8	28.32	28.47	1.14	3.74	26.05	18.25	29.71	31.96	1.01	Up	4.60E-01
CerG1(d48:3)	891.8	28.17	26.85	1.63	1.82	26.51	22.57	30.68	29.46	-1.05	Down	1.04E-01
CerG1(d48:5+pO)	903.7	20.05	15.36	7.15	0.94	2.63	14.75	25.07	18.25	-1.31	Down	1.93E-02
CerG1(d48:6)	885.7	18.62	15.36	9.04	0.94	2.63	14.75	25.23	18.25	-1.21	Down	7.93E-02
CerG1(d49:0)	911.8	35.30	29.75	0.30	3.55	34.86	23.68	35.78	35.04	-1.19	Down	2.00E-04
CerG1(d49:0+O)	927.8	26.29	22.84	1.03	4.56	24.00	14.86	27.88	29.47	-1.15	Down	2.53E-02
CerG1(d49:1)	909.8	35.56	28.78	0.61	3.75	34.63	22.60	36.45	34.86	-1.24	Down	1.00E-04
CerG1(d49:1+hO+O)	941.8	18.03	15.36	1.82	0.94	13.70	14.75	19.50	18.25	-1.17	Down	1.00E-03
CerG1(d49:1+O)	925.8	18.81	31.21	2.74	1.41	12.42	28.77	21.95	33.70	1.66	Up	1.00E-04
CerG1(d49:1+pO)	925.8	27.32	24.81	1.01	3.74	24.75	20.80	28.71	31.55	-1.1	Down	5.12E-02
CerG1(d49:2)	907.8	35.01	25.90	1.04	2.47	33.63	22.50	36.45	30.06	-1.35	Down	1.00E-04
CerG1(d49:2+pO)	923.8	26.17	15.36	3.40	0.94	21.30	14.75	29.24	18.25	-1.7	Down	1.00E-04
CerG1(d49:3)	905.8	28.56	30.87	5.56	1.78	18.45	28.57	33.37	33.80	1.08	Up	1.00E+00
CerG1(d49:4)	903.8	27.15	27.31	1.84	2.54	23.87	21.57	29.07	30.27	1.01	Up	8.61E-01
CerG1(d50:1)	923.8	30.30	30.62	0.66	1.22	29.50	27.97	31.19	32.62	1.01	Up	4.44E-01
CerG1(d50:2)	921.8	29.38	29.71	0.91	3.80	28.09	18.25	30.85	32.91	1.01	Up	6.92E-02
CerG1(d50:3)	919.8	25.36	27.78	3.58	2.16	20.03	25.16	28.54	30.35	1.1	Up	5.95E-02
CerG1(d50:4)	917.8	22.70	23.39	5.13	2.93	15.69	18.25	29.02	28.25	1.03	Up	6.89E-01
CerG1(d50:6)	913.7	23.33	27.62	6.42	3.26	12.72	18.25	28.67	31.33	1.18	Up	6.04E-02
CerG1(d51:0)	939.8	34.46	26.13	1.16	3.33	33.13	21.38	36.45	30.77	-1.32	Down	1.00E-04
CerG1(d51:0+pO)	955.8	23.50	26.19	2.01	1.53	19.43	23.29	25.80	29.04	1.11	Up	1.50E-03
CerG1(d51:1)	937.8	34.43	27.82	0.33	3.34	33.80	22.02	34.86	34.45	-1.24	Down	1.00E-04
CerG1(d51:1+O)	953.8	21.69	19.50	3.16	4.45	18.18	14.75	26.23	27.30	-1.11	Down	1.93E-01
CerG1(d51:1+pO)	953.8	24.76	15.36	1.08	0.94	23.06	14.75	25.79	18.25	-1.61	Down	1.00E-04
CerG1(d51:2)	935.8	34.27	26.96	0.52	5.09	33.63	15.07	35.04	33.13	-1.27	Down	4.00E-04
CerG1(d51:3)	933.8	33.77	34.02	0.75	1.48	31.93	31.40	34.45	35.78	1.01	Up	4.79E-01

CerG1(d51:3+pO)	949.8	22.25	15.36	1.89	0.94	19.08	14.75	25.15	18.25	-1.45	Down	1.00E-04
CerG1(d51:4)	931.8	26.97	31.78	7.61	1.81	16.03	28.52	33.23	34.10	1.18	Up	3.40E-01
CerG1(d51:5)	929.8	24.54	15.36	6.25	0.94	13.54	14.75	29.78	18.25	-1.6	Down	1.20E-03
CerG1(d52:0)	953.9	28.34	18.03	1.93	3.19	23.74	14.97	30.88	22.53	-1.57	Down	1.00E-04
CerG1(d52:4)	945.8	27.51	15.36	1.54	0.94	24.62	14.75	29.80	18.25	-1.79	Down	1.00E-04
CerG1(d52:6)	941.8	21.85	28.48	2.84	1.89	15.73	26.10	25.72	33.13	1.3	Up	1.00E-04
CerG1(d53:0)	967.9	24.49	28.36	9.66	5.64	4.21	15.07	32.55	31.93	1.16	Up	7.86E-01
CerG1(d53:1)	965.9	32.59	28.16	0.92	4.80	30.68	15.07	34.63	34.45	-1.16	Down	8.00E-04
CerG1(d53:2)	963.8	13.81	19.77	7.96	4.46	2.63	15.00	25.47	26.62	1.43	Up	1.31E-01
CerG1(d53:3)	961.8	31.73	32.00	0.56	1.26	30.79	29.32	32.70	34.10	1.01	Up	5.06E-01
CerG1(d53:6)	955.8	24.81	29.20	5.19	1.59	16.12	27.02	29.04	31.93	1.18	Up	3.74E-02
CerG1(d54:0)	981.9	22.35	15.36	4.21	0.94	16.47	14.75	27.65	18.25	-1.46	Down	1.00E-04
CerG1(d54:1)	979.9	26.57	15.36	1.77	0.94	21.55	14.75	27.90	18.25	-1.73	Down	1.00E-04
CerG1(d54:5)	971.8	21.95	15.36	4.75	0.94	16.53	14.75	28.27	18.25	-1.43	Down	1.00E-04
CerG1(d55:0)	995.9	27.69	15.36	2.07	0.94	24.37	14.75	30.30	18.25	-1.8	Down	1.00E-04
CerG1(d55:1)	993.9	27.42	15.36	2.67	0.94	24.38	14.75	31.06	18.25	-1.78	Down	1.00E-04
CerG1(d55:2)	991.9	29.67	26.07	0.87	2.66	27.90	21.23	30.92	29.88	-1.14	Down	6.00E-04
CerG1(d55:3)	989.9	21.00	30.14	5.98	1.10	15.36	28.54	29.98	31.91	1.43	Up	3.00E-04
CerG1(d56:0EO)	1040	20.74	15.36	1.68	0.94	16.19	14.75	22.61	18.25	-1.35	Down	1.00E-04
CerG1(d56:1)	1008	13.45	15.36	10.72	0.94	2.63	14.75	26.55	18.25	1.14	Up	6.88E-01
CerG1(d56:1EO)	1038	24.47	25.02	0.63	2.40	23.80	18.25	26.00	26.77	1.02	Up	8.48E-02
CerG1(d57:0EO)	1054	21.80	15.36	2.18	0.94	16.42	14.75	24.29	18.25	-1.42	Down	1.00E-04
CerG1(d57:1)	1022	25.43	15.36	3.41	0.94	20.43	14.75	29.94	18.25	-1.66	Down	1.00E-04
CerG1(d57:1+pOEO)	1068	13.10	15.36	7.73	0.94	2.63	14.75	22.60	18.25	1.17	Up	4.79E-01
CerG1(d57:1EO)	1052	24.32	24.92	0.98	2.45	23.27	18.25	26.23	27.06	1.02	Up	7.92E-02
CerG1(d57:3)	1018	28.08	18.47	1.44	4.37	25.53	14.75	30.06	27.51	-1.52	Down	1.00E-04
CerG1(d57:3+pO)	1034	16.72	15.36	5.31	0.94	2.63	14.75	22.65	18.25	-1.09	Down	3.60E-03
CerG1(d57:4)	1016	22.83	15.36	4.22	0.94	15.50	14.75	28.62	18.25	-1.49	Down	1.00E-04
CerG1(d58:0+pOEO)	1084	21.42	25.50	3.93	0.90	13.03	24.16	24.74	27.08	1.19	Up	1.00E-04
CerG1(d58:0EO)	1068	23.94	25.63	0.86	1.41	22.85	22.99	25.21	27.87	1.07	Up	2.40E-03
CerG1(d58:1EO)	1066	25.21	26.57	0.61	0.96	24.39	24.89	26.54	27.74	1.05	Up	6.00E-04
CerG1(d58:2EO)	1064	24.98	25.07	0.94	3.40	23.78	15.59	26.93	27.60	1	Up	1.86E-01
CerG1(d58:3+pO)	1048	19.29	15.36	2.23	0.94	14.10	14.75	22.46	18.25	-1.26	Down	5.00E-04
CerG1(d58:3EO)	1062	14.59	15.36	7.12	0.94	2.63	14.75	22.63	18.25	1.05	Up	1.34E-01
CerG1(d58:4+pO)	1046	17.70	15.36	5.68	0.94	2.63	14.75	23.84	18.25	-1.15	Down	1.70E-03
CerG1(d58:5+pO)	1044	10.58	15.36	6.74	0.94	2.63	14.75	18.61	18.25	1.45	Up	1.02E-01
CerG1(d58:6+hO)	1042	9.78	15.36	5.35	0.94	2.63	14.75	17.05	18.25	1.57	Up	8.00E-04
CerG1(d59:0+pOEO)	1098	15.37	15.36	8.67	0.94	2.63	14.75	24.95	18.25	-1	Down	1.66E-01

CerG1(d59:1EO)	1080	23.80	24.87	0.57	2.30	23.01	18.25	25.00	26.54	1.04	Up	8.60E-03
CerG1(d59:2EO)	1078	23.36	23.85	1.28	3.55	21.17	15.59	25.79	26.88	1.02	Up	1.34E-01
CerG1(d59:3+pO)	1062	22.82	15.36	1.21	0.94	20.80	14.75	25.30	18.25	-1.49	Down	1.00E-04
CerG1(d59:6+pO)	1056	16.78	15.36	7.67	0.94	2.63	14.75	24.35	18.25	-1.09	Down	2.89E-02
CerG1(d60:0+pOEO)	1112	19.45	15.36	8.41	0.94	2.63	14.75	24.02	18.25	-1.27	Down	1.06E-02
CerG1(d60:0EO)	1096	13.35	15.36	7.95	0.94	2.63	14.75	21.88	18.25	1.15	Up	6.01E-01
CerG1(d60:1+pO)	1080	15.52	15.36	2.71	0.94	8.17	14.75	18.27	18.25	-1.01	Down	1.90E-01
CerG1(d60:1+pOEO)	1110	19.25	24.26	8.66	2.16	2.63	18.25	24.93	26.29	1.26	Up	7.41E-02
CerG1(d60:2)	1062	16.56	15.36	1.48	0.94	14.50	14.75	19.01	18.25	-1.08	Down	5.12E-02
CerG1(d60:3+pO)	1076	23.72	24.10	1.15	3.44	21.63	15.59	25.12	26.51	1.02	Up	1.50E-02
CerG1(d60:4+pO)	1074	23.01	15.36	1.26	0.94	20.70	14.75	24.79	18.25	-1.5	Down	1.00E-04
CerG1(d62:2+pOEO)	1136	15.25	15.36	8.39	0.94	2.63	14.75	24.65	18.25	1.01	Up	3.40E-01
CerG1(d62:2EO)	1120	16.89	15.36	5.76	0.94	2.63	14.75	24.04	18.25	-1.1	Down	3.91E-02
CerG1(d64:1EO)	1150	9.36	15.36	7.24	0.94	2.63	14.75	18.47	18.25	1.64	Up	2.16E-01
CerG1(d65:3)	1130	14.81	15.36	7.86	0.94	2.63	14.75	22.99	18.25	1.04	Up	1.17E-01
CerG1(d67:0EO)	1194	11.40	15.36	9.37	0.94	2.63	14.75	23.68	18.25	1.35	Up	6.88E-01
CerG1(d68:1EO)	1206	9.86	20.84	9.23	2.87	2.63	15.58	23.86	23.65	2.11	Up	1.22E-02
CerG1(d68:2EO)	1204	7.31	18.32	7.14	3.39	2.63	15.00	20.45	22.56	2.51	Up	3.20E-03
CerG1(d73:2+pOEO)	1290	15.72	18.54	5.77	3.78	4.21	14.97	24.22	25.20	1.18	Up	5.66E-01
CerG1(d74:2EO)	1288	7.39	15.36	5.77	0.94	2.63	14.75	15.94	18.25	2.08	Up	4.00E-03
CerG1(d77:1EO)	1332	18.55	23.94	3.40	4.96	14.61	15.11	23.50	28.76	1.29	Up	8.60E-03
CerG1(d77:4EO)	1326	18.57	20.68	8.65	5.39	2.63	15.00	29.14	28.69	1.11	Up	7.86E-01
CerG1(d81:3+hOEO)	1400	10.96	15.36	11.55	0.94	2.63	14.75	36.45	18.25	1.4	Up	2.79E-01
CerG1(d83:0EO)	1418	19.44	15.36	8.10	0.94	2.63	14.75	26.21	18.25	-1.27	Down	1.06E-02
CerG1(d85:2EO)	1442	17.95	21.01	8.06	4.01	2.63	14.95	24.55	26.29	1.17	Up	5.18E-01
CerG2(d15:0+pO)	613.3	18.55	22.62	2.91	2.18	14.98	18.25	24.08	26.44	1.22	Up	1.00E-03
CerG2(d18:0)	639.4	22.64	21.27	2.83	2.91	17.39	15.58	27.69	25.18	-1.06	Down	6.01E-01
CerG2(d18:0+pO)	655.4	10.53	15.36	5.76	0.94	2.63	14.75	15.57	18.25	1.46	Up	6.91E-02
CerG2(d18:1/16:0)	861.6	22.04	24.38	1.25	2.06	20.61	18.25	24.39	26.23	1.11	Up	7.00E-04
CerG2(d18:1/18:0)	889.6	23.43	24.59	2.43	2.16	17.20	18.25	26.56	27.11	1.05	Up	7.93E-02
CerG2(d18:1/18:0+O)	905.6	13.51	15.36	6.81	0.94	2.63	14.75	23.27	18.25	1.14	Up	1.40E-01
CerG2(d18:1/24:1)	971.7	20.86	25.17	5.75	2.22	12.77	18.25	25.84	26.69	1.21	Up	1.50E-03
CerG2(d18:1+hO)	653.4	10.52	15.36	7.14	0.94	2.63	14.75	23.43	18.25	1.46	Up	5.61E-02
CerG2(d19:0)	653.4	5.52	15.36	5.34	0.94	2.63	14.75	16.49	18.25	2.78	Up	6.60E-03
CerG2(d19:1+hO)	667.4	20.33	20.05	1.56	3.83	17.97	14.97	22.70	24.62	-1.01	Down	6.51E-01
CerG2(d20:2)	663.4	17.98	23.02	2.39	2.65	13.22	15.58	21.28	26.84	1.28	Up	1.00E-04
CerG2(d26:0)	751.5	13.19	15.36	8.44	0.94	2.63	14.75	22.85	18.25	1.16	Up	8.29E-01
CerG2(d27:0)	765.5	18.90	24.00	2.53	1.70	13.64	22.50	23.16	28.50	1.27	Up	1.00E-04

CerG2(d27:0+O)	781.5	18.15	15.36	4.20	0.94	10.99	14.75	27.73	18.25	-1.18	Down	2.80E-03
CerG2(d28:0)	779.5	23.59	26.78	3.39	2.74	17.48	18.25	26.47	28.42	1.13	Up	2.00E-04
CerG2(d28:0+pO)	795.5	19.41	22.23	2.06	1.57	15.79	18.25	23.46	24.18	1.15	Up	1.30E-03
CerG2(d28:1+pO+2O)	825.5	8.80	15.36	8.07	0.94	2.63	14.75	22.63	18.25	1.75	Up	1.03E-02
CerG2(d29:0+2O)	825.5	15.68	15.36	6.49	0.94	2.63	14.75	19.57	18.25	-1.02	Down	1.63E-02
CerG2(d29:0+pO)	809.6	18.28	23.64	2.30	1.78	14.08	21.76	22.30	28.40	1.29	Up	1.00E-04
CerG2(d29:0+pO+2O)	841.5	15.52	15.36	8.32	0.94	2.63	14.75	21.59	18.25	-1.01	Down	6.92E-02
CerG2(d30:2)	803.5	21.42	29.91	3.69	2.43	17.97	26.53	28.09	35.78	1.4	Up	1.00E-04
CerG2(d30:3)	801.5	15.56	21.93	6.54	3.36	2.63	15.11	19.87	25.84	1.41	Up	3.50E-03
CerG2(d30:4+pO)	815.5	13.60	15.36	5.73	0.94	2.63	14.75	19.10	18.25	1.13	Up	9.75E-01
CerG2(d31:1+pO+2O)	867.6	4.27	15.36	3.97	0.94	2.63	14.75	15.83	18.25	3.6	Up	6.00E-04
CerG2(d31:2+2O)	849.5	9.91	15.36	10.07	0.94	2.63	14.75	27.09	18.25	1.55	Up	6.81E-02
CerG2(d31:3+pO)	831.5	6.64	17.39	5.12	2.89	2.63	14.75	15.52	21.28	2.62	Up	2.00E-04
CerG2(d32:2)	831.6	14.66	15.92	4.87	2.08	2.63	14.75	20.38	21.82	1.09	Up	7.86E-01
CerG2(d32:3+hO+2O)	877.5	7.35	15.36	6.00	0.94	2.63	14.75	15.73	18.25	2.09	Up	2.10E-03
CerG2(d33:0+O)	865.6	18.90	23.53	6.28	1.93	2.63	18.25	24.10	25.82	1.25	Up	1.00E-03
CerG2(d33:1+O)	863.6	11.04	18.17	9.56	3.84	2.63	14.86	27.15	25.14	1.65	Up	1.31E-01
CerG2(d33:1+pO)	863.6	22.01	23.75	1.70	1.87	17.58	18.25	23.52	25.73	1.08	Up	1.00E-03
CerG2(d33:3+pO)	859.6	11.38	19.65	8.94	5.06	2.63	15.00	26.21	27.49	1.73	Up	4.49E-02
CerG2(d33:5+pO)	855.5	14.04	18.63	9.06	3.13	2.63	14.86	21.33	22.06	1.33	Up	2.06E-01
CerG2(d34:0)	863.6	18.82	15.36	6.08	0.94	2.63	14.75	22.86	18.25	-1.23	Down	8.60E-03
CerG2(d34:1+O)	877.6	13.25	21.64	7.13	1.22	2.63	18.25	18.91	23.13	1.63	Up	1.00E-04
CerG2(d34:2)	859.6	5.95	15.36	4.77	0.94	2.63	14.75	13.60	18.25	2.58	Up	1.00E-04
CerG2(d34:2+O)	875.6	23.14	15.36	3.34	0.94	17.20	14.75	27.87	18.25	-1.51	Down	1.00E-04
CerG2(d34:3)	857.6	11.65	15.36	9.58	0.94	2.63	14.75	27.89	18.25	1.32	Up	1.85E-01
CerG2(d34:3+2O)	889.6	6.32	15.36	4.58	0.94	2.63	14.75	15.91	18.25	2.43	Up	7.00E-04
CerG2(d34:4)	855.6	12.80	15.36	8.15	0.94	2.63	14.75	23.61	18.25	1.2	Up	8.78E-01
CerG2(d34:6+pO)	867.5	23.69	25.60	1.24	2.48	22.04	23.15	26.47	32.25	1.08	Up	1.64E-02
CerG2(d35:0+O)	893.6	15.25	15.36	8.42	0.94	2.63	14.75	23.42	18.25	1.01	Up	2.81E-01
CerG2(d35:1+hO+O)	907.6	5.21	15.36	4.66	0.94	2.63	14.75	15.00	18.25	2.95	Up	1.00E-04
CerG2(d35:1+O)	891.6	17.77	15.36	6.34	0.94	2.63	14.75	23.45	18.25	-1.16	Down	2.88E-01
CerG2(d35:2+hO)	889.6	13.53	15.36	6.01	0.94	2.63	14.75	21.45	18.25	1.13	Up	2.55E-01
CerG2(d35:2+pO+2O)	921.6	5.02	15.36	4.26	0.94	2.63	14.75	14.08	18.25	3.06	Up	1.00E-04
CerG2(d35:3+2O)	903.6	6.11	15.36	5.84	0.94	2.63	14.75	19.38	18.25	2.51	Up	8.00E-04
CerG2(d36:0+O)	907.7	9.62	15.36	5.96	0.94	2.63	14.75	17.08	18.25	1.6	Up	7.40E-03
CerG2(d36:2+hO+2O)	935.6	7.18	15.36	5.46	0.94	2.63	14.75	15.11	18.25	2.14	Up	3.00E-04
CerG2(d36:4+2O)	915.6	5.41	15.88	3.98	1.90	2.63	14.75	13.72	21.13	2.94	Up	1.00E-04
CerG2(d37:2+pO)	917.6	14.46	22.49	8.12	3.42	2.63	14.95	22.02	28.47	1.56	Up	2.80E-03

CerG2(d37:4+pO)	913.6	7.03	15.36	5.92	0.94	2.63	14.75	17.94	18.25	2.19	Up	9.00E-04
CerG2(d38:0+pO+2O)	967.7	6.01	15.36	5.36	0.94	2.63	14.75	17.04	18.25	2.56	Up	7.00E-04
CerG2(d38:2+O)	931.7	8.37	15.36	7.10	0.94	2.63	14.75	18.07	18.25	1.84	Up	1.84E-01
CerG2(d38:2+pO+2O)	963.6	6.81	15.36	6.21	0.94	2.63	14.75	17.10	18.25	2.26	Up	4.36E-02
CerG2(d38:3+2O)	945.6	5.38	15.36	3.93	0.94	2.63	14.75	13.28	18.25	2.86	Up	1.00E-04
CerG2(d38:4+2O)	943.6	7.55	15.36	6.01	0.94	2.63	14.75	17.02	18.25	2.03	Up	9.00E-04
CerG2(d38:5+pO)	925.6	6.91	15.36	6.40	0.94	2.63	14.75	17.76	18.25	2.22	Up	3.22E-02
CerG2(d38:6+pO)	923.6	20.79	15.36	5.54	0.94	10.43	14.75	25.77	18.25	-1.35	Down	1.06E-02
CerG2(d39:0)	933.7	19.54	15.36	7.41	0.94	2.63	14.75	24.80	18.25	-1.27	Down	2.88E-01
CerG2(d39:0+O)	949.7	4.25	15.36	3.90	0.94	2.63	14.75	15.59	18.25	3.62	Up	5.00E-04
CerG2(d39:1)	931.7	9.20	15.36	6.90	0.94	2.63	14.75	19.88	18.25	1.67	Up	8.70E-03
CerG2(d39:3)	927.7	17.51	15.36	3.22	0.94	11.34	14.75	22.69	18.25	-1.14	Down	1.88E-02
CerG2(d39:6+hO)	937.6	5.25	15.36	4.78	0.94	2.63	14.75	15.78	18.25	2.92	Up	7.00E-04
CerG2(d39:7+hO)	935.6	9.44	15.36	6.32	0.94	2.63	14.75	16.51	18.25	1.63	Up	3.33E-02
CerG2(d40:2)	943.7	9.94	15.36	6.26	0.94	2.63	14.75	16.90	18.25	1.55	Up	1.85E-01
CerG2(d40:2+hO+O)	975.7	7.40	15.36	7.31	0.94	2.63	14.75	21.57	18.25	2.08	Up	5.05E-02
CerG2(d40:3)	941.7	9.79	23.59	9.05	2.63	2.63	18.25	22.49	27.95	2.41	Up	3.00E-04
CerG2(d41:1)	959.7	14.39	15.36	5.26	0.94	2.63	14.75	19.79	18.25	1.07	Up	4.79E-01
CerG2(d41:3+pO)	971.7	9.33	15.36	6.41	0.94	2.63	14.75	19.69	18.25	1.65	Up	8.70E-03
CerG2(d41:7+hO)	963.6	16.83	15.36	5.12	0.94	2.63	14.75	21.23	18.25	-1.1	Down	1.88E-02
CerG2(d43:0+pO+2O)	1038	10.75	24.15	8.43	2.60	2.63	22.01	23.21	31.30	2.25	Up	2.00E-04
CerG2(d43:0+pO+O)	1022	8.84	15.36	6.56	0.94	2.63	14.75	17.53	18.25	1.74	Up	8.98E-02
CerG2(d43:4)	981.7	20.10	15.36	4.35	0.94	13.40	14.75	27.54	18.25	-1.31	Down	5.60E-03
CerG2(d44:1+O)	1018	7.55	15.36	4.99	0.94	2.63	14.75	14.01	18.25	2.03	Up	1.00E-04
CerG2(d44:6+pO)	1008	8.60	15.36	5.95	0.94	2.63	14.75	16.28	18.25	1.79	Up	2.40E-03
CerG2(d45:1)	1016	12.96	26.40	7.93	3.27	2.63	21.78	23.83	33.37	2.04	Up	1.00E-04
CerG2(d45:3)	1012	14.34	15.36	9.04	0.94	2.63	14.75	25.75	18.25	1.07	Up	4.42E-01
CerG2(d46:4+pO)	1040	17.06	22.59	7.33	2.81	2.63	15.11	25.79	25.97	1.32	Up	1.16E-02
CerG2(d46:6)	1020	9.34	15.36	6.46	0.94	2.63	14.75	16.26	18.25	1.64	Up	8.60E-03
CerG2(d49:0+O)	1090	16.09	20.77	9.12	3.73	2.63	14.97	23.06	24.44	1.29	Up	1.66E-01
CerG2(d49:4+pO)	1082	12.56	15.36	6.20	0.94	2.63	14.75	19.31	18.25	1.22	Up	7.12E-01
CerG2(d49:5+pO)	1080	8.91	15.36	6.49	0.94	2.63	14.75	18.14	18.25	1.72	Up	4.49E-02
CerG2(d49:8)	1058	7.11	15.36	5.39	0.94	2.63	14.75	15.20	18.25	2.16	Up	5.00E-04
CerG2(d50:6+pO)	1092	8.65	15.36	6.52	0.94	2.63	14.75	17.77	18.25	1.78	Up	8.60E-03
CerG2(d51:6)	1090	8.21	17.04	7.94	4.09	2.63	14.75	22.01	27.11	2.07	Up	2.34E-02
CerG2(d52:8)	1100	11.19	23.83	8.41	5.29	2.63	14.97	24.39	33.94	2.13	Up	1.50E-03
CerG2(d54:4)	1136	7.69	15.36	5.28	0.94	2.63	14.75	15.72	18.25	2	Up	5.00E-03
CerG2(d55:5+pO)	1164	16.31	15.36	6.56	0.94	2.63	14.75	24.09	18.25	-1.06	Down	2.25E-02

CerG2(d55:6)	1146	21.85	23.72	1.72	1.22	17.53	21.50	24.19	26.25	1.09	Up	6.40E-03
CerG2(d55:7)	1144	21.33	15.36	1.97	0.94	16.05	14.75	23.50	18.25	-1.39	Down	1.00E-04
CerG2(d56:7)	1158	9.96	15.36	8.09	0.94	2.63	14.75	22.30	18.25	1.54	Up	6.87E-02
CerG2(d57:5+pO)	1192	15.13	15.36	7.47	0.94	2.63	14.75	23.48	18.25	1.02	Up	3.47E-01
CerG2(d57:6)	1174	15.01	22.82	7.40	2.79	2.63	15.59	23.54	27.90	1.52	Up	1.70E-03
CerG2(d57:6+pO)	1190	12.99	15.36	8.07	0.94	2.63	14.75	23.38	18.25	1.18	Up	9.26E-01
CerG2(d57:7)	1172	11.05	21.68	9.63	3.41	2.63	14.95	23.94	24.73	1.96	Up	1.33E-02
CerG2(d63:4+pO)	1278	8.93	18.01	7.74	3.99	2.63	14.86	23.56	26.65	2.02	Up	8.30E-03
CerG2(d70:4EO)	1390	25.10	26.13	0.82	2.86	24.16	18.25	26.81	28.54	1.04	Up	1.50E-02
CerG2(d71:2+pOEO)	1424	26.36	27.13	1.14	3.07	24.97	18.25	28.20	29.73	1.03	Up	3.74E-02
CerG2(d71:3EO)	1406	26.32	27.10	1.19	3.06	24.96	18.25	28.41	29.76	1.03	Up	4.54E-02
CerG2(d72:2+pOEO)	1438	20.45	20.99	6.10	3.86	2.63	14.95	24.77	25.97	1.03	Up	8.33E-01
CerG2(d73:4EO)	1432	9.05	20.56	8.17	3.49	2.63	14.95	21.32	24.03	2.27	Up	1.20E-03
CerG2(d74:4EO)	1446	26.13	25.00	2.02	4.12	21.21	15.59	28.97	28.92	-1.05	Down	5.25E-01
CerG2(d75:2+pOEO)	1480	22.72	25.67	7.86	3.10	2.63	18.25	27.87	29.07	1.13	Up	4.87E-01
CerG2(d75:3EO)	1462	27.16	26.38	1.77	3.35	23.16	18.25	30.04	29.94	-1.03	Down	4.98E-01
CerG2(d75:4EO)	1460	8.47	15.36	9.05	0.94	2.63	14.75	23.73	18.25	1.81	Up	6.70E-02
CerG2(d76:4EO)	1474	9.62	15.36	9.14	0.94	2.63	14.75	24.25	18.25	1.6	Up	6.81E-02
CerG2(d84:0+pOEO)	1610	18.91	15.36	8.75	0.94	2.63	14.75	25.69	18.25	-1.23	Down	1.50E-02
CerG2GNAc1(d16:0+pO)	830.4	6.71	15.36	6.08	0.94	2.63	14.75	17.36	18.25	2.29	Up	6.90E-03
CerG2GNAc1(d16:1+hO)	828.4	23.06	21.65	3.80	4.93	15.40	14.97	28.79	28.88	-1.07	Down	4.51E-01
CerG2GNAc1(d18:0)	842.5	13.95	22.42	7.56	1.46	2.63	18.25	20.47	24.00	1.61	Up	2.00E-04
CerG2GNAc1(d18:0+pO)	858.5	7.14	15.36	7.10	0.94	2.63	14.75	18.28	18.25	2.15	Up	4.93E-02
CerG2GNAc1(d18:1)	840.4	11.15	15.36	5.62	0.94	2.63	14.75	16.03	18.25	1.38	Up	6.02E-02
CerG2GNAc1(d19:0)	856.5	20.16	24.90	4.19	2.60	15.42	18.25	27.89	28.85	1.24	Up	1.56E-02
CerG2GNAc1(d19:1)	854.5	5.10	15.36	5.13	0.94	2.63	14.75	15.64	18.25	3.01	Up	4.20E-03
CerG2GNAc1(d20:0)	870.5	9.39	15.36	7.55	0.94	2.63	14.75	22.73	18.25	1.64	Up	5.14E-02
CerG2GNAc1(d20:0+pO)	886.5	13.52	20.68	7.41	2.77	2.63	15.11	19.74	23.17	1.53	Up	3.40E-03
CerG2GNAc1(d22:1)	896.5	4.35	15.36	4.23	0.94	2.63	14.75	16.72	18.25	3.53	Up	6.00E-04
CerG2GNAc1(d23:0)	912.5	4.13	16.40	3.52	2.46	2.63	14.75	14.27	21.51	3.97	Up	1.00E-04
CerG2GNAc1(d23:1)	910.5	15.49	21.08	8.91	6.77	2.63	15.00	28.65	30.62	1.36	Up	3.40E-01
CerG2GNAc1(d23:1+hO)	926.5	9.08	20.52	8.19	3.50	2.63	15.07	21.76	24.61	2.26	Up	1.40E-03
CerG2GNAc1(d24:1)	924.5	13.76	15.36	5.98	0.94	2.63	14.75	19.52	18.25	1.12	Up	6.95E-01
CerG2GNAc1(d25:0)	940.6	5.00	15.36	4.20	0.94	2.63	14.75	13.82	18.25	3.08	Up	1.00E-04
CerG2GNAc1(d25:0+pO)	956.6	6.84	15.36	6.30	0.94	2.63	14.75	17.60	18.25	2.25	Up	6.90E-03
CerG2GNAc1(d25:1)	938.6	4.15	15.36	3.60	0.94	2.63	14.75	14.53	18.25	3.7	Up	1.00E-04
CerG2GNAc1(d26:0)	954.6	22.30	26.42	2.03	2.58	18.34	22.63	24.19	32.62	1.18	Up	2.00E-04
CerG2GNAc1(d26:1+hO)	968.6	20.20	23.81	8.01	4.96	5.89	15.11	29.71	31.30	1.18	Up	2.03E-01

CerG2GNAc1(d26:2)	950.6	6.37	15.36	6.13	0.94	2.63	14.75	17.51	18.25	2.41	Up	6.60E-03
CerG2GNAc1(d27:0)	968.6	4.38	15.36	4.31	0.94	2.63	14.75	16.99	18.25	3.51	Up	6.00E-04
CerG2GNAc1(d27:1)	966.6	6.69	15.36	6.01	0.94	2.63	14.75	16.70	18.25	2.3	Up	2.34E-02
CerG2GNAc1(d28:0)	982.6	10.31	15.36	6.19	0.94	2.63	14.75	19.20	18.25	1.49	Up	5.23E-02
CerG2GNAc1(d28:0+2O)	1015	4.93	15.36	4.10	0.94	2.63	14.75	14.15	18.25	3.11	Up	1.00E-04
CerG2GNAc1(d28:2)	978.6	6.41	15.36	4.45	0.94	2.63	14.75	13.40	18.25	2.4	Up	1.00E-04
CerG2GNAc1(d29:1+hO)	1011	6.61	15.36	5.91	0.94	2.63	14.75	16.94	18.25	2.32	Up	6.90E-03
CerG2GNAc1(d29:2+hO)	1009	23.18	22.56	3.84	4.41	17.22	15.15	29.71	29.38	-1.03	Down	7.23E-01
CerG2GNAc1(d30:2+pO+2O)	1055	17.40	20.02	6.25	5.48	4.21	15.00	25.43	27.99	1.15	Up	2.12E-01
CerG2GNAc1(d30:4+pO)	1019	15.24	20.45	6.98	6.00	2.63	15.00	22.43	29.08	1.34	Up	1.17E-01
CerG2GNAc1(d31:1+pO+2O)	1071	20.44	20.63	3.02	5.42	16.35	15.00	25.51	28.40	1.01	Up	1.00E+00
CerG2GNAc1(d32:1+pO+2O)	1085	17.97	23.49	6.97	3.54	2.63	15.11	28.47	27.84	1.31	Up	3.28E-02
CerG2GNAc1(d32:5)	1029	17.10	21.29	9.26	5.26	2.63	15.07	27.43	28.77	1.24	Up	3.72E-01
CerG2GNAc1(d34:0+pO)	1083	4.26	15.36	3.95	0.94	2.63	14.75	15.75	18.25	3.6	Up	6.00E-04
CerG2GNAc1(d34:2+2O)	1095	18.61	15.36	4.28	0.94	14.85	14.75	25.86	18.25	-1.21	Down	2.46E-02
CerG2GNAc1(d34:5+hO)	1073	8.16	15.36	6.80	0.94	2.63	14.75	17.42	18.25	1.88	Up	1.84E-01
CerG2GNAc1(d35:0+pO)	1097	5.19	15.36	4.15	0.94	2.63	14.75	15.24	18.25	2.96	Up	5.00E-04
CerG2GNAc1(d35:2+2O)	1109	6.20	15.36	5.73	0.94	2.63	14.75	18.88	18.25	2.48	Up	5.70E-03
CerG2GNAc1(d35:3+2O)	1107	11.50	15.36	6.74	0.94	2.63	14.75	19.41	18.25	1.34	Up	3.40E-01
CerG2GNAc1(d36:0+pO)	1111	8.14	15.36	6.42	0.94	2.63	14.75	17.56	18.25	1.89	Up	1.03E-02
CerG2GNAc1(d38:3)	1117	7.12	15.36	5.37	0.94	2.63	14.75	14.27	18.25	2.16	Up	1.00E-04
CerG2GNAc1(d38:5+pO)	1129	10.32	15.36	7.16	0.94	2.63	14.75	19.18	18.25	1.49	Up	5.58E-01
CerG2GNAc1(d41:0)	1165	15.47	23.53	8.77	5.61	2.63	14.97	26.54	31.58	1.52	Up	1.50E-02
CerG2GNAc1(d41:0+pO+2O)	1213	11.16	15.36	6.57	0.94	2.63	14.75	17.85	18.25	1.38	Up	1.85E-01
CerG2GNAc1(d41:2+pO)	1177	12.80	24.04	9.25	2.36	2.63	21.32	25.63	28.54	1.88	Up	2.30E-03
CerG2GNAc1(d41:3+pO)	1175	10.30	15.36	8.13	0.94	2.63	14.75	22.17	18.25	1.49	Up	5.37E-01
CerG2GNAc1(d47:4)	1241	8.50	15.36	6.32	0.94	2.63	14.75	19.50	18.25	1.81	Up	6.00E-03
CerG2GNAc1(d48:5+pO)	1269	15.89	15.36	7.59	0.94	2.63	14.75	24.91	18.25	-1.03	Down	3.10E-01
CerG2GNAc1(d48:6)	1251	15.26	15.36	8.22	0.94	2.63	14.75	24.80	18.25	1.01	Up	3.72E-01
CerG2GNAc1(d51:7+hO)	1307	14.26	15.36	7.58	0.94	2.63	14.75	23.10	18.25	1.08	Up	1.86E-01
CerG2GNAc1(d55:0)	1361	7.91	15.36	6.61	0.94	2.63	14.75	18.36	18.25	1.94	Up	5.14E-02
CerG2GNAc1(d59:0EO)	1447	11.37	20.97	9.59	3.91	2.63	14.95	25.35	26.65	1.84	Up	2.85E-02
CerG2GNAc1(d61:3)	1439	25.95	25.70	0.82	4.18	24.28	15.59	27.10	28.47	-1.01	Down	1.06E-02
CerG2GNAc1(d63:0EO)	1503	29.64	29.83	1.11	4.11	28.50	18.25	31.51	32.83	1.01	Up	6.04E-02
CerG2GNAc1(d63:2)	1469	4.24	21.02	3.88	3.86	2.63	14.95	15.52	25.84	4.96	Up	1.00E-04
CerG2GNAc1(d63:2+pO)	1485	23.26	15.36	3.00	0.94	18.30	14.75	28.69	18.25	-1.51	Down	1.00E-04
CerG2GNAc1(d63:2EO)	1499	23.94	23.46	1.27	3.63	21.68	15.58	26.43	28.54	-1.02	Down	6.73E-01
CerG2GNAc1(d63:5)	1463	19.86	22.89	6.64	3.34	2.63	15.58	24.10	27.85	1.15	Up	3.16E-01

CerG2GNAc1(d64:3)	1481	6.63	15.36	6.58	0.94	2.63	14.75	18.06	18.25	2.32	Up	1.49E-02
CerG2GNAc1(d64:3+hOEO)	1527	18.29	22.23	10.26	3.76	2.63	15.58	25.67	26.29	1.22	Up	9.75E-01
CerG2GNAc1(d64:3+pO)	1497	27.43	28.06	0.98	3.47	24.82	18.25	28.90	30.74	1.02	Up	1.06E-02
CerG2GNAc1(d64:4)	1479	27.43	28.01	0.96	3.44	24.79	18.25	28.42	30.30	1.02	Up	3.37E-02
CerG2GNAc1(d64:5)	1477	16.38	15.36	10.91	0.94	2.63	14.75	24.69	18.25	-1.07	Down	2.80E-01
CerG2GNAc1(d64:5+pO)	1493	14.73	24.50	9.67	2.48	2.63	18.25	23.74	27.71	1.66	Up	8.00E-04
CerG2GNAc1(d64:6)	1475	16.26	24.28	10.82	2.34	2.63	18.25	24.86	27.66	1.49	Up	2.85E-02
CerG2GNAc1(d64:6+pO)	1491	24.10	22.80	1.71	4.05	19.98	15.58	26.25	26.62	-1.06	Down	8.33E-01
CerG2GNAc1(d65:0)	1501	19.84	15.86	5.99	1.78	2.63	14.86	23.58	20.70	-1.25	Down	1.30E-03
CerG2GNAc1(d65:3)	1495	15.76	24.20	10.42	2.18	2.63	18.25	23.80	26.18	1.53	Up	6.60E-03
CerG2GNAc1(d65:3+pO)	1511	16.40	15.36	9.70	0.94	2.63	14.75	27.97	18.25	-1.07	Down	1.48E-01
CerG2GNAc1(d65:3EO)	1525	13.79	15.36	8.45	0.94	2.63	14.75	25.61	18.25	1.11	Up	7.82E-01
CerG2GNAc1(d65:5)	1491	26.50	26.41	1.36	3.34	22.69	18.25	28.01	29.80	-1	Down	2.88E-01
CerG2GNAc1(d65:6)	1489	23.87	23.55	1.62	3.37	19.38	15.59	25.15	26.89	-1.01	Down	7.58E-01
CerG2GNAc1(d66:3EO)	1539	9.70	20.32	8.97	4.09	2.63	14.95	23.04	24.98	2.09	Up	8.60E-03
CerG2GNAc1(d67:0EO)	1559	27.60	25.95	1.70	4.61	22.99	15.59	29.52	30.51	-1.06	Down	6.08E-01
CerG2GNAc1(d67:2EO)	1555	30.27	29.88	0.99	4.14	28.16	18.25	31.87	32.97	-1.01	Down	2.07E-01
CerG2GNAc1(d67:3EO)	1553	16.64	23.50	9.65	3.50	2.63	15.59	27.61	27.95	1.41	Up	4.52E-02
CerG2GNAc1(d67:4EO)	1551	24.36	24.30	1.41	3.81	22.29	15.59	26.84	28.25	-1	Down	2.88E-01
CerG2GNAc1(d68:0EO)	1573	24.56	23.68	1.39	4.54	21.14	15.58	25.95	27.68	-1.04	Down	2.60E-01
CerG2GNAc1(d68:2EO)	1569	17.97	24.24	10.34	2.32	2.63	18.25	25.36	26.71	1.35	Up	4.06E-01
CerG2GNAc1(d69:1+pOEO)	1601	7.05	15.36	5.76	0.94	2.63	14.75	18.45	18.25	2.18	Up	9.00E-04
CerG2GNAc1(d69:3EO)	1581	17.49	15.36	8.53	0.94	2.63	14.75	27.88	18.25	-1.14	Down	3.17E-02
CerG2GNAc1(d69:4EO)	1579	18.24	15.36	8.31	0.94	2.63	14.75	27.22	18.25	-1.19	Down	1.29E-02
CerG2GNAc1(d71:1EO)	1613	16.78	15.36	9.82	0.94	2.63	14.75	24.14	18.25	-1.09	Down	2.81E-01
CerG2GNAc1(d71:2EO)	1611	27.50	26.70	1.10	4.00	24.54	15.59	28.63	30.63	-1.03	Down	1.00E+00
CerG2GNAc1(d71:4EO)	1607	25.15	25.29	1.53	3.31	21.53	15.59	27.79	28.30	1.01	Up	2.42E-01
CerG2GNAc1(d72:2EO)	1625	18.15	15.36	8.22	0.94	2.63	14.75	23.36	18.25	-1.18	Down	2.09E-02
CerG2GNAc1(d73:0EO)	1643	11.21	15.36	11.05	0.94	2.63	14.75	26.22	18.25	1.37	Up	2.79E-01
CerG2GNAc1(d78:0EO)	1713	19.05	15.36	8.73	0.94	2.63	14.75	24.97	18.25	-1.24	Down	1.50E-02
CerG2GNAc1(d78:2EO)	1709	4.65	15.36	5.20	0.94	2.63	14.75	20.01	18.25	3.3	Up	8.00E-04
CerG2GNAc1(d80:1EO)	1739	8.66	19.64	7.63	3.45	2.63	14.95	20.44	23.13	2.27	Up	1.40E-03
CerG2GNAc1(d84:0EO)	1797	12.96	27.68	11.21	1.84	2.63	25.36	27.06	32.48	2.14	Up	3.00E-04
CerG2GNAc1(d84:1EO)	1795	10.73	15.36	10.36	0.94	2.63	14.75	25.15	18.25	1.43	Up	2.79E-01
CerG2GNAc1(d85:0EO)	1811	14.99	27.97	9.76	2.25	2.63	25.58	26.28	33.80	1.87	Up	1.00E-04
CerG2GNAc1(d85:1EO)	1809	16.92	18.86	7.10	4.09	2.63	14.86	20.95	23.74	1.11	Up	8.78E-01
CerG3(d15:0+pO)	775.4	13.43	22.08	7.16	2.95	2.63	15.15	20.52	26.24	1.64	Up	4.00E-04
CerG3(d18:0)	801.4	4.16	15.36	4.12	0.94	2.63	14.75	16.23	18.25	3.69	Up	5.00E-04

CerG3(d18:1/16:0)	1024	20.30	23.75	1.09	1.89	19.04	18.25	22.38	25.61	1.17	Up	3.00E-04
CerG3(d18:1/18:0)	1052	19.61	24.64	5.99	2.23	2.63	18.25	23.30	27.33	1.26	Up	1.00E-04
CerG3(d21:0+pO)	859.5	3.90	15.36	2.82	0.94	2.63	14.75	11.82	18.25	3.93	Up	1.00E-04
CerG3(d21:1+hO)	857.5	18.81	27.14	6.40	1.52	2.63	24.16	24.58	29.46	1.44	Up	1.00E-04
CerG3(d22:1)	855.5	4.18	15.36	3.67	0.94	2.63	14.75	14.80	18.25	3.68	Up	1.00E-04
CerG3(d24:0)	885.5	6.39	15.36	4.43	0.94	2.63	14.75	13.29	18.25	2.4	Up	1.00E-04
CerG3(d25:0)	899.5	4.08	15.36	3.37	0.94	2.63	14.75	13.76	18.25	3.76	Up	1.00E-04
CerG3(d25:0+pO)	915.5	14.28	15.36	5.99	0.94	2.63	14.75	19.72	18.25	1.08	Up	4.42E-01
CerG3(d25:1)	897.5	6.70	15.36	5.46	0.94	2.63	14.75	15.14	18.25	2.29	Up	1.10E-03
CerG3(d26:0+pO)	929.6	5.23	15.36	4.52	0.94	2.63	14.75	15.83	18.25	2.94	Up	7.00E-04
CerG3(d26:0+pO+O)	945.6	18.02	25.61	1.20	1.35	16.12	23.70	19.77	28.13	1.42	Up	1.00E-04
CerG3(d26:2)	909.5	10.94	21.20	10.10	4.52	2.63	15.00	28.54	29.40	1.94	Up	1.22E-02
CerG3(d27:1)	925.6	10.11	17.70	7.83	3.35	2.63	14.75	19.62	23.36	1.75	Up	3.87E-02
CerG3(d27:1+pO)	941.6	3.98	15.36	3.06	0.94	2.63	14.75	12.65	18.25	3.86	Up	1.00E-04
CerG3(d28:0)	941.6	11.15	15.36	5.98	0.94	2.63	14.75	16.62	18.25	1.38	Up	5.59E-01
CerG3(d28:0+2O)	973.6	19.64	15.36	8.49	0.94	2.63	14.75	29.24	18.25	-1.28	Down	1.06E-02
CerG3(d28:1+hO)	955.6	5.03	15.36	4.26	0.94	2.63	14.75	13.68	18.25	3.06	Up	1.00E-04
CerG3(d29:0)	955.6	14.68	15.36	8.15	0.94	2.63	14.75	26.18	18.25	1.05	Up	1.66E-01
CerG3(d29:0+2O)	987.6	12.01	15.36	9.20	0.94	2.63	14.75	27.11	18.25	1.28	Up	1.66E-01
CerG3(d29:0+pO)	971.6	25.21	28.22	0.96	2.73	23.64	25.30	27.49	34.76	1.12	Up	3.00E-03
CerG3(d29:1)	953.6	24.38	28.19	0.93	2.68	23.16	25.38	26.87	34.63	1.16	Up	1.00E-04
CerG3(d29:1+hO)	969.6	5.55	15.36	5.43	0.94	2.63	14.75	17.53	18.25	2.77	Up	2.00E-03
CerG3(d30:0)	969.6	15.29	15.36	7.47	0.94	2.63	14.75	26.62	18.25	1	Up	1.03E-01
CerG3(d30:1)	967.6	11.44	17.05	7.65	3.66	2.63	14.75	19.83	25.67	1.49	Up	4.78E-01
CerG3(d30:3)	963.6	21.32	21.07	6.92	6.76	5.89	15.00	29.76	30.62	-1.01	Down	8.80E-01
CerG3(d30:3+pO)	979.6	20.34	20.79	2.08	2.91	18.30	15.15	25.00	24.51	1.02	Up	3.40E-01
CerG3(d30:4)	961.6	6.37	15.36	5.58	0.94	2.63	14.75	16.97	18.25	2.41	Up	3.90E-03
CerG3(d31:0)	983.6	9.49	15.36	9.36	0.94	2.63	14.75	27.37	18.25	1.62	Up	1.12E-02
CerG3(d31:0+O)	999.6	7.67	15.36	5.81	0.94	2.63	14.75	18.98	18.25	2	Up	1.00E-03
CerG3(d31:1+pO+2O)	1030	6.70	15.36	6.04	0.94	2.63	14.75	17.20	18.25	2.29	Up	2.34E-02
CerG3(d31:3)	977.6	18.26	15.36	2.42	0.94	15.13	14.75	22.30	18.25	-1.19	Down	2.00E-04
CerG3(d32:0)	997.7	8.22	15.36	8.76	0.94	2.63	14.75	23.90	18.25	1.87	Up	5.83E-02
CerG3(d33:1)	1010	14.35	15.36	10.29	0.94	2.63	14.75	29.52	18.25	1.07	Up	5.17E-01
CerG3(d34:3+hO+2O)	1068	9.69	15.36	6.06	0.94	2.63	14.75	17.53	18.25	1.58	Up	3.62E-02
CerG3(d35:5+pO)	1046	7.12	22.43	5.39	5.00	2.63	15.00	14.97	27.93	3.15	Up	1.00E-04
CerG3(d37:4+pO)	1076	25.08	28.09	0.96	2.61	23.74	24.86	27.01	34.17	1.12	Up	2.20E-03
CerG3(d37:5)	1058	24.98	28.09	0.92	2.65	23.78	24.86	26.81	34.30	1.12	Up	1.90E-03
CerG3(d37:6+hO)	1072	18.40	15.36	4.91	0.94	5.89	14.75	23.93	18.25	-1.2	Down	1.30E-03

CerG3(d38:1+hO+O)	1112	7.54	15.36	6.35	0.94	2.63	14.75	20.78	18.25	2.04	Up	9.00E-04
CerG3(d38:1+pO+2O)	1128	6.10	15.36	5.27	0.94	2.63	14.75	17.39	18.25	2.52	Up	7.00E-04
CerG3(d38:5+pO)	1088	6.78	15.36	6.20	0.94	2.63	14.75	17.87	18.25	2.26	Up	8.30E-03
CerG3(d39:0+pO)	1112	8.15	15.36	5.89	0.94	2.63	14.75	15.48	18.25	1.88	Up	6.00E-04
CerG3(d39:2)	1092	7.51	15.36	5.90	0.94	2.63	14.75	15.73	18.25	2.05	Up	8.00E-04
CerG3(d41:5+pO)	1130	6.03	15.36	5.02	0.94	2.63	14.75	16.10	18.25	2.55	Up	7.00E-04
CerG3(d44:1)	1164	16.22	23.09	5.83	2.34	2.63	21.08	23.88	29.16	1.42	Up	1.70E-03
CerG3(d56:1)	1332	5.13	15.36	5.26	0.94	2.63	14.75	17.46	18.25	2.99	Up	6.00E-04
CerG3(d58:3+hO)	1372	6.44	15.36	5.61	0.94	2.63	14.75	16.39	18.25	2.39	Up	7.00E-04
CerG3(d58:8)	1346	14.02	15.36	9.50	0.94	2.63	14.75	27.74	18.25	1.1	Up	3.55E-01
CerG3(d59:0)	1376	6.25	15.36	5.97	0.94	2.63	14.75	17.66	18.25	2.46	Up	4.50E-03
CerG3(d60:3+hOEO)	1430	8.61	15.36	7.62	0.94	2.63	14.75	22.65	18.25	1.78	Up	1.64E-01
CerG3(d61:0+pO)	1420	11.43	15.36	11.40	0.94	2.63	14.75	27.81	18.25	1.34	Up	2.79E-01
CerG3(d61:5)	1394	9.85	20.98	9.34	3.92	2.63	14.95	24.93	26.49	2.13	Up	1.46E-02
CerG3(d62:0)	1418	5.41	15.36	5.10	0.94	2.63	14.75	16.10	18.25	2.84	Up	1.00E-03
CerG3(d62:1+pOEO)	1462	25.19	25.12	1.22	2.58	21.82	18.25	26.88	27.68	-1	Down	6.89E-01
CerG3(d63:0EO)	1462	20.63	27.11	10.38	3.12	2.63	18.25	28.71	29.52	1.31	Up	1.78E-02
CerG3(d64:0+pO)	1462	9.23	15.36	8.30	0.94	2.63	14.75	20.95	18.25	1.66	Up	2.53E-01
CerG3(d64:0+pOEO)	1492	10.52	15.36	10.19	0.94	2.63	14.75	26.39	18.25	1.46	Up	2.79E-01
CerG3(d64:0EO)	1476	15.74	15.36	9.09	0.94	2.63	14.75	27.77	18.25	-1.02	Down	1.66E-01
CerG3(d65:1EO)	1488	16.84	15.36	8.28	0.94	2.63	14.75	27.01	18.25	-1.1	Down	3.37E-02
CerG3(d65:2+pOEO)	1502	4.31	15.36	4.11	0.94	2.63	14.75	16.30	18.25	3.56	Up	6.00E-04
CerG3(d65:5)	1450	25.62	25.44	1.42	4.20	23.78	15.59	28.25	29.12	-1.01	Down	2.12E-01
CerG3(d66:0+pOEO)	1520	30.56	29.92	0.82	4.12	29.64	18.25	31.78	32.62	-1.02	Down	1.85E-01
CerG3(d66:1+pOEO)	1518	20.42	15.36	3.06	0.94	17.36	14.75	25.58	18.25	-1.33	Down	1.00E-04
CerG3(d66:2+pOEO)	1516	24.78	24.60	1.13	3.89	22.25	15.59	26.47	28.85	-1.01	Down	1.66E-01
CerG3(d67:1EO)	1516	13.18	15.36	6.97	0.94	2.63	14.75	20.84	18.25	1.17	Up	8.29E-01
CerG3(d67:2EO)	1514	18.87	25.15	9.39	2.81	2.63	18.25	25.88	27.95	1.33	Up	2.66E-02
CerG3(d68:0EO)	1532	8.23	15.36	8.64	0.94	2.63	14.75	22.52	18.25	1.87	Up	6.70E-02
CerG3(d68:2+pOEO)	1544	16.12	15.36	9.16	0.94	2.63	14.75	26.16	18.25	-1.05	Down	1.31E-01
CerG3(d69:1+pOEO)	1560	16.81	15.36	9.55	0.94	2.63	14.75	24.23	18.25	-1.09	Down	2.81E-01
CerG3(d70:0+pOEO)	1576	27.87	26.59	1.35	4.90	24.40	15.59	29.32	30.54	-1.05	Down	7.40E-01
CerG3(d70:2EO)	1556	4.14	15.36	3.56	0.94	2.63	14.75	14.42	18.25	3.71	Up	1.00E-04
CerG3(d71:0+pOEO)	1590	24.38	15.36	1.95	0.94	20.45	14.75	26.53	18.25	-1.59	Down	1.00E-04
CerG3(d73:1EO)	1600	10.32	15.36	9.78	0.94	2.63	14.75	23.59	18.25	1.49	Up	2.79E-01
CerG3(d75:2EO)	1626	17.12	22.59	11.49	3.98	2.63	15.58	25.94	26.56	1.32	Up	6.88E-01
CerG3GNAc1(d14:0)	948.5	5.46	15.36	5.24	0.94	2.63	14.75	17.24	18.25	2.82	Up	7.00E-04
CerG3GNAc1(d15:0+pO)	978.5	5.20	15.36	4.75	0.94	2.63	14.75	16.60	18.25	2.95	Up	7.00E-04

CerG3GNAc1(d18:0+pO)	1021	7.25	15.36	6.61	0.94	2.63	14.75	19.60	18.25	2.12	Up	1.00E-03
CerG3GNAc1(d20:0)	1033	6.89	15.36	4.32	0.94	2.63	14.75	13.40	18.25	2.23	Up	1.00E-04
CerG3GNAc1(d20:1)	1031	5.35	15.36	4.97	0.94	2.63	14.75	15.27	18.25	2.87	Up	2.00E-03
CerG3GNAc1(d21:1)	1045	5.86	15.36	5.90	0.94	2.63	14.75	19.47	18.25	2.62	Up	3.70E-03
CerG3GNAc1(d22:1)	1059	6.67	15.36	5.71	0.94	2.63	14.75	15.72	18.25	2.3	Up	5.70E-03
CerG3GNAc1(d34:1)	1227	19.63	23.31	1.61	1.80	16.17	18.25	22.30	25.28	1.19	Up	6.00E-04
CerG3GNAc1(d35:4)	1235	10.50	19.17	9.02	4.44	2.63	15.00	22.85	25.61	1.82	Up	3.29E-02
CerG3GNAc1(d36:1)	1255	20.36	23.62	1.46	1.94	18.59	18.25	23.16	25.72	1.16	Up	1.20E-03
CerG3GNAc1(d38:4)	1277	9.88	21.35	7.74	2.16	2.63	15.59	21.20	25.12	2.16	Up	3.00E-04
CerG3GNAc1(d39:2)	1295	9.27	16.01	7.23	2.27	2.63	14.75	18.95	22.60	1.73	Up	1.46E-01
CerG3GNAc1(d43:3)	1349	6.21	15.36	5.21	0.94	2.63	14.75	14.68	18.25	2.47	Up	1.00E-04
CerG3GNAc1(d45:3+pO)	1393	5.06	15.36	4.44	0.94	2.63	14.75	15.78	18.25	3.04	Up	7.00E-04
CerG3GNAc1(d45:6)	1371	8.87	15.36	8.04	0.94	2.63	14.75	24.17	18.25	1.73	Up	2.05E-01
CerG3GNAc1(d46:5)	1387	10.11	22.86	8.16	4.30	2.63	15.11	24.73	31.42	2.26	Up	2.80E-03
CerG3GNAc1(d47:6)	1399	8.37	15.36	6.46	0.94	2.63	14.75	18.24	18.25	1.83	Up	1.03E-02
CerG3GNAc1(d49:2)	1435	6.17	15.36	5.48	0.94	2.63	14.75	15.31	18.25	2.49	Up	7.00E-04
CerG3GNAc1(d50:1+O)	1467	15.14	15.36	9.93	0.94	2.63	14.75	22.73	18.25	1.01	Up	2.80E-01
CerG3GNAc1(d50:7+hO)	1455	11.53	15.36	9.38	0.94	2.63	14.75	22.54	18.25	1.33	Up	7.34E-01
CerG3GNAc1(d51:0+pO+O)	1499	7.78	15.36	6.66	0.94	2.63	14.75	19.71	18.25	1.98	Up	1.03E-02
CerG3GNAc1(d53:4)	1487	10.11	15.36	8.42	0.94	2.63	14.75	20.49	18.25	1.52	Up	4.04E-01
CerG3GNAc1(d58:1)	1563	20.72	22.67	3.11	4.14	14.85	14.97	23.43	27.58	1.09	Up	5.95E-02
CerG3GNAc1(d60:2+pOEO)	1635	4.29	15.36	4.04	0.94	2.63	14.75	16.05	18.25	3.58	Up	6.00E-04
CerG3GNAc1(d61:5)	1597	25.04	23.50	1.39	3.56	21.46	14.95	26.58	26.76	-1.07	Down	3.72E-01
CerG3GNAc1(d62:0EO)	1651	6.40	15.36	5.55	0.94	2.63	14.75	16.30	18.25	2.4	Up	1.10E-03
CerG3GNAc1(d83:0EO)	1946	11.43	25.56	8.02	4.25	2.63	15.11	20.31	32.35	2.24	Up	2.00E-04
CerG3GNAc1(d84:0EO)	1960	10.62	24.41	6.07	3.94	2.63	15.11	18.85	31.78	2.3	Up	1.00E-04
CerG3GNAc2(d22:1)	1262	6.42	15.36	6.18	0.94	2.63	14.75	16.86	18.25	2.39	Up	9.60E-03
CerG3GNAc2(d23:0+pO)	1294	7.27	15.36	5.56	0.94	2.63	14.75	16.46	18.25	2.11	Up	1.20E-03
CerG3GNAc2(d38:1+hO+O)	1518	6.48	15.36	6.14	0.94	2.63	14.75	19.03	18.25	2.37	Up	2.70E-03
CerG3GNAc2(d41:0+pO+O)	1562	6.19	15.36	5.26	0.94	2.63	14.75	15.91	18.25	2.48	Up	1.10E-03
CerG3GNAc2(d42:4)	1536	8.05	15.36	7.07	0.94	2.63	14.75	22.50	18.25	1.91	Up	8.60E-03
CerG3GNAc2(d44:5)	1562	5.38	15.36	4.11	0.94	2.63	14.75	13.64	18.25	2.85	Up	1.00E-04
CerG3GNAc2(d44:7)	1558	7.33	15.36	6.26	0.94	2.63	14.75	16.33	18.25	2.1	Up	1.30E-02
CerG3GNAc2(d47:2)	1610	6.22	15.36	5.53	0.94	2.63	14.75	17.39	18.25	2.47	Up	6.90E-03
CerG3GNAc2(d47:4)	1606	8.24	15.36	6.57	0.94	2.63	14.75	21.00	18.25	1.86	Up	1.40E-03
CerG3GNAc2(d50:0)	1656	4.59	15.36	5.01	0.94	2.63	14.75	19.37	18.25	3.35	Up	8.00E-04
CerG3GNAc2(d53:0+pO)	1714	5.50	15.36	6.56	0.94	2.63	14.75	21.77	18.25	2.79	Up	3.20E-03
CerG3GNAc2(d54:0+pO)	1728	7.37	15.36	6.32	0.94	2.63	14.75	20.03	18.25	2.08	Up	8.60E-03

CerG3GNAc2(d59:1+pO)	1796	5.70	15.36	5.73	0.94	2.63	14.75	17.42	18.25	2.7	Up	6.60E-03
CerG3GNAc2(d59:3+pO)	1792	5.22	15.36	5.06	0.94	2.63	14.75	15.48	18.25	2.94	Up	1.20E-03
CerG3GNAc2(d62:2)	1820	4.07	15.36	3.34	0.94	2.63	14.75	13.64	18.25	3.77	Up	1.00E-04
CerG3GNAc2(d62:5)	1814	4.55	15.36	4.89	0.94	2.63	14.75	18.95	18.25	3.37	Up	8.00E-04
CerG3GNAc2(d64:2)	1848	5.50	15.36	5.68	0.94	2.63	14.75	17.07	18.25	2.79	Up	6.20E-03
CerP(d14:0+pO)	355.2	8.45	15.36	7.22	0.94	2.63	14.75	21.84	18.25	1.82	Up	1.00E-02
CerP(d15:0+pO)	369.2	18.73	19.74	2.52	3.61	15.05	14.86	22.29	23.73	1.05	Up	5.66E-01
CerP(d15:0+pO/8:0)	481.3	25.58	15.36	0.80	0.94	24.28	14.75	27.35	18.25	-1.67	Down	1.00E-04
CerP(d18:0+pO/33:2)	869.7	21.56	22.88	2.64	1.98	19.18	18.25	26.88	25.93	1.06	Up	1.16E-01
CerP(d18:1/32:2)	837.7	24.73	28.17	6.91	2.08	14.68	23.84	29.98	30.82	1.14	Up	6.51E-01
CerP(d18:1/32:3)	835.7	21.47	27.37	8.27	1.54	2.63	24.37	28.52	29.34	1.28	Up	2.46E-02
CerP(d18:1/33:2)	851.7	26.75	15.36	5.60	0.94	17.69	14.75	31.03	18.25	-1.74	Down	1.00E-04
CerP(d18:1/46:1EO)	1066	20.57	15.36	4.03	0.94	16.76	14.75	27.51	18.25	-1.34	Down	1.00E-04
CerP(d20:0+pO/27:5)	807.6	21.74	15.36	9.10	0.94	2.63	14.75	28.63	18.25	-1.42	Down	7.92E-02
CerP(d21:0)	437.3	24.28	24.46	1.03	2.15	22.90	18.25	25.84	26.26	1.01	Up	2.18E-01
CerP(d23:0)	465.3	24.61	24.46	0.87	2.11	23.69	18.25	26.74	26.02	-1.01	Down	3.72E-01
CerP(d23:1)	463.3	25.31	25.44	0.80	2.40	24.29	18.25	27.02	27.73	1.01	Up	6.88E-02
CerP(d29:2+hO)	561.4	19.85	15.36	5.33	0.94	5.89	14.75	26.91	18.25	-1.29	Down	4.00E-04
CerP(d32:0)	591.5	21.18	22.98	1.61	1.72	17.43	18.25	23.98	25.16	1.09	Up	5.10E-03
CerP(d33:1)	603.5	9.22	19.52	7.86	3.51	2.63	14.95	20.11	23.53	2.12	Up	1.70E-03
CerP(d33:5)	595.4	21.73	23.26	1.49	2.06	19.19	18.25	24.57	25.93	1.07	Up	5.69E-02
CerP(d34:2)	615.5	19.74	15.36	3.63	0.94	13.24	14.75	25.46	18.25	-1.29	Down	1.30E-03
CerP(d37:4)	653.5	16.69	20.79	7.37	3.80	2.63	15.07	25.82	26.04	1.25	Up	2.07E-01
CerP(d38:6)	663.5	7.47	15.36	6.81	0.94	2.63	14.75	18.63	18.25	2.06	Up	3.65E-02
CerP(d39:0)	689.6	17.79	15.36	6.51	0.94	2.63	14.75	25.44	18.25	-1.16	Down	2.81E-01
CerP(d40:0)	703.6	9.62	15.36	8.87	0.94	2.63	14.75	22.62	18.25	1.6	Up	2.79E-01
CerP(d40:0+O)	719.6	16.59	15.36	2.72	0.94	12.54	14.75	19.24	18.25	-1.08	Down	3.25E-01
CerP(d40:0+pO+2O)	751.6	19.99	23.70	6.03	1.98	2.63	18.25	24.41	25.75	1.19	Up	4.50E-03
CerP(d40:5)	693.5	19.24	15.36	2.77	0.94	14.15	14.75	21.91	18.25	-1.25	Down	8.10E-03
CerP(d41:5)	707.5	8.23	27.25	7.18	2.91	2.63	18.25	21.06	29.71	3.31	Up	1.00E-04
CerP(d41:7+hO)	719.5	4.41	18.09	4.41	3.30	2.63	14.75	17.33	22.19	4.11	Up	2.00E-04
CerP(d42:0)	731.6	16.21	15.36	9.31	0.94	2.63	14.75	24.17	18.25	-1.06	Down	2.81E-01
CerP(d42:4+pO)	739.6	17.93	15.36	3.95	0.94	11.26	14.75	22.57	18.25	-1.17	Down	1.34E-01
CerP(d42:5)	721.5	23.52	24.71	1.24	2.10	22.50	18.25	25.89	26.27	1.05	Up	2.66E-02
CerP(d42:8)	715.5	28.71	23.01	1.82	1.96	25.85	21.60	31.74	28.41	-1.25	Down	1.00E-04
CerP(d43:2)	741.6	27.38	28.62	0.94	3.32	26.05	18.25	28.79	30.47	1.05	Up	1.00E-03
CerP(d43:3)	739.6	32.28	32.93	0.62	1.73	31.30	28.48	33.23	34.45	1.02	Up	4.88E-02
CerP(d43:3+pO)	755.6	18.57	24.45	5.82	0.75	2.63	22.70	22.83	25.65	1.32	Up	1.00E-04

CerP(d43:4)	737.6	26.31	27.43	1.13	2.93	25.29	18.25	28.38	29.24	1.04	Up	1.06E-02
CerP(d44:0)	759.7	13.27	15.36	10.56	0.94	2.63	14.75	26.65	18.25	1.16	Up	9.26E-01
CerP(d44:3)	753.6	27.03	15.36	0.60	0.94	25.80	14.75	27.81	18.25	-1.76	Down	1.00E-04
CerP(d44:4+pO)	767.6	23.57	26.49	1.85	0.94	19.60	24.98	26.56	28.09	1.12	Up	3.00E-04
CerP(d44:5)	749.6	25.33	27.73	1.40	0.82	23.47	26.53	27.25	28.90	1.09	Up	1.00E-04
CerP(d44:5+pO)	765.6	25.84	25.14	4.12	2.60	17.51	18.25	29.46	27.84	-1.03	Down	1.34E-01
CerP(d45:0)	773.7	20.88	26.81	7.59	1.68	2.63	23.93	27.43	29.46	1.28	Up	1.06E-02
CerP(d45:2)	769.6	20.39	15.36	6.57	0.94	14.88	14.75	29.67	18.25	-1.33	Down	3.60E-03
CerP(d45:3)	767.6	32.65	33.33	0.97	1.73	30.95	29.88	33.94	35.04	1.02	Up	1.09E-01
CerP(d45:4)	765.6	30.16	30.80	1.02	1.82	28.90	26.43	31.71	32.77	1.02	Up	3.17E-01
CerP(d45:5)	763.6	23.01	15.36	5.35	0.94	15.24	14.75	27.74	18.25	-1.5	Down	2.00E-04
CerP(d45:6)	761.6	25.80	20.57	0.74	2.89	25.06	14.97	27.14	24.39	-1.25	Down	1.00E-04
CerP(d45:6+pO)	777.6	29.63	15.36	1.54	0.94	27.08	14.75	31.42	18.25	-1.93	Down	1.00E-04
CerP(d45:7)	759.6	14.23	15.36	7.91	0.94	2.63	14.75	26.73	18.25	1.08	Up	8.33E-01
CerP(d45:8)	757.5	9.43	15.36	9.45	0.94	2.63	14.75	25.51	18.25	1.63	Up	2.03E-01
CerP(d46:1+O)	801.7	12.09	15.36	5.27	0.94	2.63	14.75	17.76	18.25	1.27	Up	1.48E-01
CerP(d46:4)	779.6	26.88	15.36	0.91	0.94	25.94	14.75	29.10	18.25	-1.75	Down	1.00E-04
CerP(d46:5)	777.6	16.19	15.36	8.73	0.94	2.63	14.75	22.33	18.25	-1.05	Down	6.92E-02
CerP(d46:6+pO)	791.6	23.00	24.92	2.37	2.58	18.12	18.25	25.33	27.76	1.08	Up	3.12E-02
CerP(d46:7)	773.6	7.43	15.36	7.50	0.94	2.63	14.75	22.19	18.25	2.07	Up	1.00E-02
CerP(d46:7+hO)	789.6	6.92	16.66	8.41	3.08	2.63	14.75	23.80	24.63	2.41	Up	6.60E-03
CerP(d46:8)	771.6	6.92	25.69	8.40	1.86	2.63	23.13	23.78	28.63	3.72	Up	1.00E-04
CerP(d47:0)	801.7	24.21	29.79	5.17	1.48	16.39	27.46	29.42	31.42	1.23	Up	6.00E-04
CerP(d47:1)	799.7	22.84	15.36	9.44	0.94	2.63	14.75	31.14	18.25	-1.49	Down	7.93E-02
CerP(d47:3)	795.7	27.69	29.96	3.28	1.47	22.02	26.21	30.65	31.40	1.08	Up	2.68E-02
CerP(d47:4+pO)	809.6	16.90	24.05	8.76	3.76	2.63	18.25	28.62	29.12	1.42	Up	2.59E-02
CerP(d47:5)	791.6	16.92	24.07	8.77	3.75	2.63	18.25	28.65	29.14	1.42	Up	2.61E-02
CerP(d47:5+pO)	807.6	23.92	26.66	3.70	2.97	18.84	18.25	29.40	29.52	1.11	Up	7.92E-02
CerP(d47:6)	789.6	25.05	26.80	2.67	2.85	20.47	18.25	27.73	29.88	1.07	Up	1.50E-02
CerP(d47:6+pO)	805.6	28.27	27.96	0.96	3.42	25.78	18.25	29.50	30.98	-1.01	Down	2.68E-01
CerP(d47:7+hO)	803.6	23.10	15.36	1.73	0.94	19.83	14.75	24.91	18.25	-1.5	Down	1.00E-04
CerP(d48:1)	813.7	20.60	15.36	9.93	0.94	2.63	14.75	27.41	18.25	-1.34	Down	2.10E-02
CerP(d48:2+pO)	827.7	22.71	15.36	1.52	0.94	19.56	14.75	23.75	18.25	-1.48	Down	1.00E-04
CerP(d48:3)	809.7	22.74	15.36	1.54	0.94	19.56	14.75	23.75	18.25	-1.48	Down	1.00E-04
CerP(d49:0)	829.7	30.03	30.54	4.76	2.08	22.04	26.58	34.38	33.13	1.02	Up	4.42E-01
CerP(d49:1)	827.7	27.77	29.75	1.18	1.78	25.44	26.69	30.04	33.13	1.07	Up	5.40E-03
CerP(d49:2)	825.7	26.77	27.17	0.43	1.94	25.85	23.19	27.51	29.38	1.01	Up	5.01E-01
CerP(d49:3)	823.7	26.23	29.59	5.52	3.70	14.05	18.25	30.45	32.20	1.13	Up	2.09E-02

CerP(d49:4)	821.7	25.93	29.56	4.82	3.67	18.89	18.25	30.12	31.68	1.14	Up	5.60E-03
CerP(d50:0)	843.7	23.72	29.24	9.64	3.66	2.63	18.25	30.95	32.40	1.23	Up	2.95E-01
CerP(d50:1)	841.7	23.19	16.98	2.37	3.99	19.11	14.75	25.97	28.13	-1.37	Down	5.00E-04
CerP(d50:1+pO)	857.7	24.44	15.36	2.41	0.94	21.48	14.75	28.22	18.25	-1.59	Down	1.00E-04
CerP(d50:2)	839.7	24.47	15.36	2.41	0.94	21.63	14.75	28.30	18.25	-1.59	Down	1.00E-04
CerP(d50:3)	837.7	16.46	15.36	2.55	0.94	13.50	14.75	21.90	18.25	-1.07	Down	2.95E-01
CerP(d50:3+pO)	853.7	16.49	15.36	1.99	0.94	14.03	14.75	20.53	18.25	-1.07	Down	3.47E-01
CerP(d50:5)	833.7	20.09	15.36	9.74	0.94	2.63	14.75	27.11	18.25	-1.31	Down	1.03E-01
CerP(d51:0)	857.8	34.31	35.88	0.77	1.29	32.97	31.96	35.04	36.45	1.05	Up	6.00E-04
CerP(d51:1)	855.7	27.92	29.77	2.21	2.68	24.48	22.43	30.68	32.83	1.07	Up	2.88E-02
CerP(d51:2)	853.7	28.16	28.21	1.42	2.23	25.34	23.39	30.45	30.58	1	Up	9.47E-01
CerP(d51:3)	851.7	28.70	25.48	2.01	4.50	25.37	15.58	31.14	33.70	-1.13	Down	3.92E-02
CerP(d51:4)	849.7	33.00	33.65	0.49	1.57	32.48	30.79	34.30	35.26	1.02	Up	1.39E-01
CerP(d51:5)	847.7	24.60	30.19	4.54	3.87	20.56	18.25	30.47	32.40	1.23	Up	6.10E-03
CerP(d51:5+pO)	863.7	26.14	26.91	0.88	1.58	24.22	24.40	27.18	28.92	1.03	Up	1.70E-01
CerP(d52:0)	871.8	27.77	29.18	2.18	3.58	24.40	18.25	30.51	32.08	1.05	Up	2.27E-02
CerP(d52:3)	865.7	28.25	22.12	0.55	4.21	27.49	14.95	29.38	27.39	-1.28	Down	4.00E-04
CerP(d52:3+pO)	881.7	21.45	15.36	3.64	0.94	15.59	14.75	25.60	18.25	-1.4	Down	2.00E-04
CerP(d52:4)	863.7	31.71	31.89	0.67	1.94	29.98	29.36	32.48	34.17	1.01	Up	8.29E-01
CerP(d52:5+pO)	877.7	25.28	25.28	1.22	2.60	21.98	18.25	26.22	27.77	-1	Down	2.81E-01
CerP(d52:6)	859.7	25.40	15.36	2.94	0.94	18.32	14.75	27.97	18.25	-1.65	Down	1.00E-04
CerP(d52:6+pO)	875.7	26.12	26.87	1.06	3.15	23.65	18.25	27.54	30.08	1.03	Up	1.18E-01
CerP(d53:0)	885.8	33.81	35.10	0.46	1.04	33.23	32.08	34.45	35.78	1.04	Up	7.00E-04
CerP(d53:0+pO+O)	917.8	21.70	15.36	5.05	0.94	15.67	14.75	28.25	18.25	-1.41	Down	1.00E-04
CerP(d53:1)	883.8	28.21	32.73	5.00	1.44	20.22	30.42	32.97	34.38	1.16	Up	1.05E-02
CerP(d53:2)	881.8	30.55	18.48	1.77	3.84	28.62	14.75	33.46	24.03	-1.65	Down	1.00E-04
CerP(d53:3)	879.7	30.56	15.36	1.25	0.94	28.04	14.75	31.68	18.25	-1.99	Down	1.00E-04
CerP(d53:4)	877.7	30.53	30.77	1.17	1.56	27.33	28.87	31.96	32.83	1.01	Up	8.05E-01
CerP(d54:0)	899.8	27.81	15.36	1.28	0.94	24.75	14.75	29.73	18.25	-1.81	Down	1.00E-04
CerP(d54:1)	897.8	25.77	15.36	4.18	0.94	15.16	14.75	29.04	18.25	-1.68	Down	1.00E-04
CerP(d54:3)	893.8	20.51	15.36	4.97	0.94	14.68	14.75	27.88	18.25	-1.34	Down	1.70E-03
CerP(d54:4)	891.7	22.10	15.36	8.77	0.94	2.63	14.75	28.27	18.25	-1.44	Down	3.63E-02
CerP(d54:4+pO)	907.7	20.55	15.36	4.73	0.94	15.22	14.75	27.38	18.25	-1.34	Down	1.00E-04
CerP(d55:0)	913.8	33.20	26.31	0.43	3.39	32.40	21.25	33.80	31.83	-1.26	Down	1.00E-04
CerP(d55:1)	911.8	33.51	34.02	0.84	0.98	32.62	31.68	34.76	35.39	1.02	Up	1.02E-01
CerP(d55:2)	909.8	34.93	33.73	1.30	1.57	31.66	30.95	36.45	35.58	-1.04	Down	3.28E-02
CerP(d55:3)	907.8	27.04	29.34	5.77	2.49	17.21	25.82	34.86	32.08	1.08	Up	4.06E-01
CerP(d55:4)	905.8	24.37	15.36	4.39	0.94	19.35	14.75	30.58	18.25	-1.59	Down	1.00E-04

CerP(d55:5)	903.7	25.95	15.36	3.15	0.94	19.84	14.75	29.50	18.25	-1.69	Down	1.00E-04
CerP(d55:7)	899.7	17.46	15.36	8.26	0.94	2.63	14.75	26.34	18.25	-1.14	Down	7.93E-02
CerP(d56:0+pO)	943.8	17.03	15.36	6.34	0.94	4.21	14.75	22.14	18.25	-1.11	Down	7.92E-02
CerP(d56:1)	925.8	26.67	18.14	2.58	4.42	20.88	14.75	29.00	28.59	-1.47	Down	1.10E-03
CerP(d56:2)	923.8	23.88	26.88	5.00	3.14	15.52	18.25	28.37	30.42	1.13	Up	2.07E-01
CerP(d56:2+pOEO)	969.8	8.13	15.36	5.53	0.94	2.63	14.75	15.84	18.25	1.89	Up	8.00E-04
CerP(d56:3+pO)	937.8	12.51	15.36	9.58	0.94	2.63	14.75	23.83	18.25	1.23	Up	6.88E-01
CerP(d56:4)	919.8	14.53	17.53	9.62	4.28	2.63	14.75	23.77	28.35	1.21	Up	8.78E-01
CerP(d57:0)	941.9	31.85	25.15	0.89	4.54	30.15	15.07	33.37	31.10	-1.27	Down	3.00E-04
CerP(d57:1)	939.8	32.58	25.21	1.06	4.11	31.40	15.07	34.63	30.01	-1.29	Down	1.00E-04
CerP(d57:2)	937.8	29.02	22.23	4.70	3.77	19.59	14.86	32.83	27.23	-1.31	Down	4.20E-03
CerP(d57:3)	935.8	33.03	34.66	3.47	1.37	22.86	30.65	34.86	35.58	1.05	Up	7.10E-03
CerP(d57:4)	933.8	29.02	29.27	6.64	2.16	15.75	25.85	34.38	31.58	1.01	Up	3.10E-01
CerP(d58:0)	955.9	25.49	16.91	2.68	2.76	18.67	14.75	28.05	21.60	-1.51	Down	1.00E-04
CerP(d58:4)	947.8	20.08	15.36	7.01	0.94	2.63	14.75	25.73	18.25	-1.31	Down	1.06E-02
CerP(d59:0)	969.9	22.89	15.36	8.13	0.94	4.21	14.75	30.37	18.25	-1.49	Down	7.00E-04
CerP(d59:1)	967.9	26.25	15.36	3.78	0.94	20.62	14.75	31.42	18.25	-1.71	Down	1.00E-04
CerP(d59:3)	963.8	31.66	31.75	0.53	2.04	30.22	26.08	32.14	34.38	1	Up	4.41E-01
CerP(d59:4)	961.8	31.30	31.37	0.42	0.98	30.77	29.90	32.02	33.04	1	Up	8.19E-01
CerP(d59:5)	959.8	25.59	32.30	9.40	1.73	2.63	27.60	31.96	34.17	1.26	Up	7.00E-04
CerP(d59:6)	957.8	25.13	29.50	5.94	1.57	16.56	26.16	30.42	31.83	1.17	Up	1.09E-01
CerP(d60:2)	979.9	8.69	15.36	6.49	0.94	2.63	14.75	17.94	18.25	1.77	Up	7.35E-02
CerP(d61:1)	995.9	19.87	15.36	8.79	0.94	4.21	14.75	29.14	18.25	-1.29	Down	1.29E-02
CerP(d61:2)	993.9	24.21	15.36	3.27	0.94	19.43	14.75	28.65	18.25	-1.58	Down	1.00E-04
CerP(d61:3)	991.9	28.47	15.36	0.90	0.94	27.46	14.75	30.12	18.25	-1.85	Down	1.00E-04
CerP(d62:0+pOEO)	1058	15.15	15.36	7.60	0.94	2.63	14.75	23.95	18.25	1.01	Up	1.51E-01
CerP(d63:0)	1026	12.66	15.36	10.95	0.94	2.63	14.75	25.98	18.25	1.21	Up	6.88E-01
CerP(d63:1)	1024	16.72	15.36	9.79	0.94	2.63	14.75	27.66	18.25	-1.09	Down	8.05E-01
CerP(d63:1+pO)	1040	6.32	15.36	4.84	0.94	2.63	14.75	17.20	18.25	2.43	Up	7.00E-04
CerP(d63:2)	1022	18.31	15.36	8.41	0.94	2.63	14.75	26.71	18.25	-1.19	Down	1.78E-02
CerP(d64:3+hOEO)	1080	22.59	22.63	1.87	2.99	17.30	15.59	24.02	25.65	1	Up	7.12E-01
CerP(d64:3EO)	1064	19.63	15.36	6.91	0.94	2.63	14.75	24.77	18.25	-1.28	Down	1.16E-02
CerP(d65:4+pO)	1062	18.59	15.36	4.70	0.94	10.08	14.75	23.73	18.25	-1.21	Down	2.88E-01
CerP(d66:0EO)	1098	16.97	15.36	2.02	0.94	12.09	14.75	19.11	18.25	-1.1	Down	3.37E-02
CerP(d76:3EO)	1232	4.60	15.36	3.55	0.94	2.63	14.75	13.74	18.25	3.34	Up	1.00E-04
CerP(d79:3+hOEO)	1290	13.82	18.54	6.26	3.77	2.63	14.97	18.47	25.20	1.34	Up	3.10E-01
CerP(d81:1EO)	1306	13.50	15.36	8.59	0.94	2.63	14.75	22.32	18.25	1.14	Up	3.72E-01
CerP(d83:2EO)	1332	12.79	23.41	9.24	4.63	2.63	15.11	24.13	27.73	1.83	Up	2.80E-03

SM(d16:0+pO)	468.3	19.04	22.81	3.74	3.74	12.99	21.21	23.56	27.16	1.2	Up	5.95E-02
SM(d16:1/16:0)	674.5	24.35	15.36	4.71	4.71	17.22	14.75	28.48	18.25	-1.59	Down	1.00E-04
SM(d16:1/18:3)	696.5	12.34	15.36	9.58	9.58	2.63	14.75	25.02	18.25	1.25	Up	6.00E-01
SM(d16:1/19:1)	714.6	20.62	25.01	7.02	7.02	2.63	18.25	25.51	27.00	1.21	Up	1.26E-02
SM(d16:1/21:3)	738.6	21.16	15.36	2.57	2.57	17.08	14.75	23.53	18.25	-1.38	Down	1.00E-04
SM(d16:1/24:1)	784.6	23.07	15.36	5.72	5.72	15.59	14.75	30.25	18.25	-1.5	Down	1.00E-04
SM(d17:0/19:0)	732.6	20.82	15.36	11.88	11.88	2.63	14.75	29.16	18.25	-1.36	Down	6.92E-02
SM(d17:0/25:0)	816.7	26.38	27.59	2.39	2.39	23.93	18.25	29.88	29.55	1.05	Up	2.55E-01
SM(d17:1/16:0)	688.6	27.32	27.99	1.28	1.28	25.97	18.25	29.73	29.82	1.02	Up	3.92E-02
SM(d17:1/18:0)	716.6	26.62	15.36	2.97	2.97	23.04	14.75	30.95	18.25	-1.73	Down	1.00E-04
SM(d17:1/18:3)	710.5	12.16	15.36	9.44	9.44	2.63	14.75	23.93	18.25	1.26	Up	9.26E-01
SM(d17:1/20:3)	738.6	17.80	23.73	4.45	4.45	13.80	18.25	25.03	26.21	1.33	Up	4.20E-03
SM(d18:0/18:1)	730.6	29.40	15.36	4.92	4.92	18.55	14.75	35.39	18.25	-1.91	Down	1.00E-04
SM(d18:0+pO/16:1)	718.6	22.36	23.22	1.59	1.59	20.84	18.25	26.05	24.51	1.04	Up	6.94E-02
SM(d18:1/15:0)	688.6	16.09	24.03	10.68	10.68	2.63	18.25	24.21	25.98	1.49	Up	3.10E-03
SM(d18:1/16:0)	702.6	33.47	33.24	0.62	0.62	32.83	29.24	34.45	34.76	-1.01	Down	5.79E-01
SM(d18:1/16:1)	700.6	27.95	29.38	1.14	1.14	26.54	27.66	30.30	30.25	1.05	Up	2.00E-03
SM(d18:1/17:1)	714.6	17.23	15.36	6.97	6.97	2.63	14.75	25.75	18.25	-1.12	Down	9.76E-01
SM(d18:1/18:0)	730.6	29.65	33.53	5.78	5.78	20.52	30.08	35.04	35.26	1.13	Up	7.93E-02
SM(d18:1/18:1)	728.6	31.35	31.89	0.95	0.95	30.19	27.82	32.70	33.80	1.02	Up	3.87E-01
SM(d18:1/18:3)	724.6	23.72	15.36	3.95	3.95	17.26	14.75	27.00	18.25	-1.54	Down	1.00E-04
SM(d18:1/19:0)	744.6	27.56	28.30	1.28	1.28	26.15	18.25	30.08	30.37	1.03	Up	4.39E-02
SM(d18:1/19:1)	742.6	24.18	25.24	1.24	1.24	21.28	18.25	25.93	27.05	1.04	Up	1.56E-02
SM(d18:1/20:1)	756.6	27.44	15.36	1.02	1.02	25.95	14.75	28.87	18.25	-1.79	Down	1.00E-04
SM(d18:1/20:2)	754.6	18.71	15.36	8.45	8.45	2.63	14.75	24.67	18.25	-1.22	Down	6.04E-02
SM(d18:1/20:3)	752.6	28.23	28.62	0.72	0.72	27.14	18.25	29.36	31.93	1.01	Up	2.10E-02
SM(d18:1/22:0)	786.7	26.28	31.31	6.92	6.92	14.66	27.68	31.83	33.13	1.19	Up	2.68E-02
SM(d18:1/22:1)	784.6	26.53	29.39	4.09	4.09	19.57	18.25	30.30	31.68	1.11	Up	5.60E-03
SM(d18:1/23:0)	800.7	30.32	30.96	0.43	0.43	29.73	26.59	31.37	32.70	1.02	Up	6.03E-02
SM(d18:1/23:1)	798.7	30.11	30.87	0.54	0.54	29.22	25.46	31.10	32.55	1.03	Up	3.91E-02
SM(d18:1/24:0)	814.7	30.19	15.36	0.48	0.48	29.50	14.75	30.82	18.25	-1.97	Down	1.00E-04
SM(d18:1/24:1)	812.7	33.91	34.39	0.58	0.58	33.04	30.74	35.26	35.58	1.01	Up	4.74E-02
SM(d18:1/24:2)	810.7	30.34	31.57	0.70	0.70	29.52	26.46	31.42	33.37	1.04	Up	1.06E-02
SM(d18:1/25:0)	828.7	29.33	15.36	0.75	0.75	28.71	14.75	31.14	18.25	-1.91	Down	1.00E-04
SM(d18:1/25:1)	826.7	31.85	31.48	0.64	0.64	30.42	27.61	32.62	33.89	-1.01	Down	5.97E-01
SM(d18:1/25:2)	824.7	27.41	28.29	0.67	0.67	26.77	27.18	28.85	29.55	1.03	Up	1.15E-02
SM(d18:1/26:0)	842.7	28.26	28.59	0.78	0.78	26.66	25.77	28.95	30.06	1.01	Up	1.66E-01
SM(d18:1/26:1)	840.7	31.14	31.02	0.87	0.87	29.04	28.09	32.48	33.29	-1	Down	8.38E-01

SM(d18:1/26:2)	838.7	27.52	28.56	0.44	0.44	27.04	26.95	28.63	30.12	1.04	Up	2.25E-02
SM(d18:1/26:4)	834.7	15.82	27.28	8.41	8.41	2.63	24.79	26.51	29.76	1.72	Up	1.10E-03
SM(d18:1/27:1)	854.7	25.81	25.55	1.08	1.08	23.50	18.25	27.74	27.81	-1.01	Down	4.42E-01
SM(d18:1/28:4)	862.7	20.73	15.36	6.63	6.63	2.63	14.75	25.67	18.25	-1.35	Down	5.00E-04
SM(d18:2/18:1)	726.6	23.21	24.45	1.27	1.27	20.77	18.25	24.69	26.21	1.05	Up	3.50E-03
SM(d18:2/18:2)	724.6	21.95	27.27	4.67	4.67	18.25	18.25	28.42	29.67	1.24	Up	1.78E-02
SM(d18:2/18:3)	722.5	13.57	15.36	8.98	8.98	2.63	14.75	24.67	18.25	1.13	Up	1.00E+00
SM(d18:2/20:3)	750.6	25.75	26.95	0.94	0.94	23.96	24.40	27.58	28.72	1.05	Up	8.70E-03
SM(d18:2/22:1)	782.6	24.81	26.19	1.48	1.48	21.14	18.25	25.95	28.13	1.06	Up	8.00E-04
SM(d20:1/19:0)	772.6	28.01	28.52	0.59	0.59	27.35	18.25	29.24	30.42	1.02	Up	8.90E-03
SM(d20:1+hO/16:0)	746.6	20.89	22.89	2.35	2.35	15.16	18.25	23.63	24.32	1.1	Up	1.38E-02
SM(d30:1)	646.5	22.89	24.32	1.10	1.10	21.35	18.25	25.37	26.71	1.06	Up	7.40E-03
SM(d30:3)	642.5	10.25	15.36	6.10	6.10	2.63	14.75	16.22	18.25	1.5	Up	1.48E-02
SM(d31:1)	660.5	22.15	24.03	1.24	1.24	20.24	18.25	24.66	25.56	1.08	Up	3.60E-03
SM(d31:2)	658.5	20.45	15.36	3.45	3.45	14.19	14.75	24.90	18.25	-1.33	Down	2.30E-03
SM(d32:0)	676.6	23.69	25.46	0.96	0.96	22.67	18.25	25.56	27.16	1.07	Up	4.00E-04
SM(d32:1)	674.5	26.58	28.09	2.50	2.50	23.10	18.25	30.51	29.80	1.06	Up	1.03E-01
SM(d32:2)	672.5	24.29	15.36	0.92	0.92	23.01	14.75	25.91	18.25	-1.58	Down	1.00E-04
SM(d32:5)	666.5	16.29	15.36	7.00	7.00	2.63	14.75	27.59	18.25	-1.06	Down	7.42E-02
SM(d33:0)	690.6	21.94	15.36	0.73	0.73	21.18	14.75	23.34	18.25	-1.43	Down	1.00E-04
SM(d33:4)	682.5	26.87	15.36	0.90	0.90	24.67	14.75	27.89	18.25	-1.75	Down	1.00E-04
SM(d33:5)	680.5	18.94	26.27	2.98	2.98	17.12	24.74	27.19	31.19	1.39	Up	2.00E-04
SM(d34:0)	704.6	28.68	30.25	0.86	0.86	27.41	26.39	29.90	31.74	1.05	Up	2.80E-03
SM(d34:0+pO)	720.6	18.90	24.10	2.47	2.47	16.39	18.25	22.90	25.53	1.27	Up	1.00E-04
SM(d34:1)	702.6	11.50	21.07	6.74	6.74	2.63	15.58	20.01	22.37	1.83	Up	2.00E-04
SM(d34:1+pO)	718.6	22.22	15.36	2.25	2.25	17.15	14.75	24.16	18.25	-1.45	Down	1.00E-04
SM(d34:2)	700.6	26.53	21.00	4.04	4.04	17.22	15.59	29.60	22.66	-1.26	Down	2.80E-03
SM(d34:2+hO)	716.5	20.55	15.36	2.60	2.60	15.79	14.75	24.86	18.25	-1.34	Down	1.00E-04
SM(d34:4)	696.5	20.39	23.56	2.83	2.83	15.27	18.25	23.09	24.98	1.16	Up	1.00E-04
SM(d34:5+hO)	710.5	17.03	15.36	6.23	6.23	2.63	14.75	28.59	18.25	-1.11	Down	3.74E-02
SM(d34:6+pO)	708.5	12.81	15.36	8.08	8.08	2.63	14.75	19.27	18.25	1.2	Up	3.09E-01
SM(d35:0)	718.6	21.44	15.36	2.10	2.10	17.26	14.75	23.29	18.25	-1.4	Down	1.00E-04
SM(d35:1)	716.6	25.24	23.65	4.71	4.71	16.05	22.20	29.27	25.60	-1.07	Down	2.88E-01
SM(d35:4)	710.5	19.23	22.72	2.78	2.78	14.10	18.25	21.93	24.37	1.18	Up	3.00E-04
SM(d35:5+pO)	724.5	19.58	15.36	7.42	7.42	2.63	14.75	28.41	18.25	-1.27	Down	1.29E-02
SM(d36:0+pO)	748.6	18.21	23.35	2.83	2.83	14.77	18.25	23.00	25.09	1.28	Up	1.00E-04
SM(d36:3)	726.6	18.20	15.36	4.80	4.80	13.84	14.75	25.34	18.25	-1.18	Down	5.25E-01
SM(d36:5)	722.5	20.82	23.46	1.73	1.73	17.93	18.25	22.63	24.57	1.13	Up	1.00E-04

SM(d37:0+pO)	762.6	21.21	15.36	3.18	3.18	15.59	14.75	25.49	18.25	-1.38	Down	1.00E-04
SM(d37:5)	736.6	11.27	15.36	6.92	6.92	2.63	14.75	18.03	18.25	1.36	Up	6.44E-01
SM(d38:1)	758.6	31.28	19.71	0.59	0.59	30.37	15.15	31.96	22.14	-1.59	Down	1.00E-04
SM(d38:2)	756.6	14.09	27.43	8.70	8.70	2.63	18.25	29.20	29.42	1.95	Up	1.00E-03
SM(d38:2+hO)	772.6	23.11	15.36	2.51	2.51	19.01	14.75	25.32	18.25	-1.5	Down	1.00E-04
SM(d38:4)	752.6	19.99	15.36	8.97	8.97	2.63	14.75	25.75	18.25	-1.3	Down	1.27E-02
SM(d38:6)	748.6	25.65	27.12	1.82	1.82	20.81	24.98	27.20	29.30	1.06	Up	1.18E-01
SM(d38:7+hO)	762.5	7.13	15.36	5.73	5.73	2.63	14.75	17.64	18.25	2.15	Up	7.10E-03
SM(d39:4)	766.6	21.73	15.36	3.99	3.99	14.61	14.75	25.61	18.25	-1.41	Down	7.00E-04
SM(d39:6+pO)	778.6	21.84	15.36	2.69	2.69	19.08	14.75	25.97	18.25	-1.42	Down	1.00E-04
SM(d40:1)	786.7	24.26	15.36	5.54	5.54	19.01	14.75	31.62	18.25	-1.58	Down	1.00E-04
SM(d40:4+pO)	796.6	11.65	26.01	9.81	9.81	2.63	18.25	25.39	28.63	2.23	Up	4.00E-04
SM(d40:6+pO)	792.6	18.74	21.27	3.77	3.77	11.60	14.95	22.69	24.13	1.13	Up	2.25E-02
SM(d41:0)	802.7	27.25	18.59	0.58	0.58	26.48	14.86	28.38	26.87	-1.47	Down	1.00E-04
SM(d41:3)	796.6	25.00	25.11	0.83	0.83	23.58	18.25	26.43	26.69	1	Up	7.92E-02
SM(d41:4)	794.6	19.36	15.36	3.29	3.29	13.45	14.75	22.44	18.25	-1.26	Down	2.27E-02
SM(d41:6+pO)	806.6	21.04	15.36	4.97	4.97	14.19	14.75	25.16	18.25	-1.37	Down	2.68E-02
SM(d42:0)	816.7	21.73	15.36	9.46	9.46	2.63	14.75	28.48	18.25	-1.41	Down	2.88E-01
SM(d42:1)	814.7	25.32	15.36	3.99	3.99	19.65	14.75	31.45	18.25	-1.65	Down	1.00E-04
SM(d42:1+pO)	830.7	26.50	27.53	0.48	0.48	25.90	18.25	27.23	30.35	1.04	Up	1.20E-03
SM(d42:4+pO)	824.6	12.86	24.12	9.92	9.92	2.63	18.25	26.37	26.10	1.88	Up	4.10E-03
SM(d42:5+pO)	822.6	15.04	15.36	7.69	7.69	2.63	14.75	24.98	18.25	1.02	Up	2.60E-01
SM(d43:0)	830.7	25.69	27.00	0.71	0.71	24.44	24.87	27.06	28.20	1.05	Up	1.00E-03
SM(d44:0)	844.7	22.56	30.24	7.14	7.14	15.44	25.61	31.74	33.37	1.34	Up	3.92E-02
SM(d44:4+pO)	852.7	16.43	15.36	6.62	6.62	4.21	14.75	25.31	18.25	-1.07	Down	1.17E-01
SM(d44:5+pO)	850.7	13.38	15.36	9.79	9.79	2.63	14.75	25.91	18.25	1.15	Up	8.29E-01
SM(d45:6)	846.7	11.78	15.36	4.44	4.44	2.63	14.75	17.77	18.25	1.3	Up	4.50E-03
SM(d46:0)	872.8	28.69	34.24	7.70	7.70	16.68	30.54	34.86	35.78	1.19	Up	2.62E-02
SM(d46:1)	870.8	31.50	32.21	0.79	0.79	29.52	29.30	32.48	34.76	1.02	Up	2.46E-01
SM(d46:2)	868.7	26.65	26.84	1.53	1.53	24.18	21.53	28.27	34.10	1.01	Up	1.00E+00
SM(d46:4)	864.7	14.08	15.36	7.59	7.59	2.63	14.75	20.61	18.25	1.09	Up	3.40E-01
SM(d46:4+pO)	880.7	8.95	15.36	7.32	7.32	2.63	14.75	21.30	18.25	1.72	Up	1.46E-01
SM(d46:5+pO)	878.7	13.11	15.36	9.40	9.40	2.63	14.75	24.92	18.25	1.17	Up	7.82E-01
SM(d46:7+hO)	874.7	14.15	15.36	8.67	8.67	2.63	14.75	25.02	18.25	1.09	Up	3.72E-01
SM(d47:1)	884.8	23.79	27.14	4.77	4.77	15.27	24.13	28.25	29.82	1.14	Up	1.03E-01
SM(d47:7+hO)	888.7	7.33	15.36	6.17	6.17	2.63	14.75	17.48	18.25	2.1	Up	6.90E-03
SM(d48:0)	900.8	22.31	15.36	10.21	10.21	4.21	14.75	32.77	18.25	-1.45	Down	1.06E-02
SM(d48:1)	898.8	32.59	33.34	1.01	1.01	30.42	30.85	33.89	34.86	1.02	Up	1.67E-01

SM(d48:3+hO)	910.8	8.32	15.36	7.09	7.09	2.63	14.75	18.95	18.25	1.85	Up	5.92E-02
SM(d48:7+hO)	902.7	14.71	15.36	8.30	8.30	2.63	14.75	24.12	18.25	1.04	Up	3.72E-01
SM(d49:3)	908.8	20.89	23.84	3.87	3.87	13.74	21.59	24.71	26.48	1.14	Up	3.57E-02
SM(d50:0)	928.8	31.17	31.27	0.62	0.62	29.60	27.49	31.87	33.80	1	Up	9.75E-01
SM(d50:1)	926.8	30.49	31.04	0.52	0.52	29.76	28.65	31.37	33.89	1.02	Up	2.02E-01
SM(d50:1+hO)	942.8	8.25	15.36	7.07	7.07	2.63	14.75	19.54	18.25	1.86	Up	1.03E-02
SM(d50:3)	922.8	24.97	28.71	7.52	7.52	13.74	18.25	30.92	31.42	1.15	Up	6.01E-01
SM(d50:4)	920.8	28.92	28.58	0.80	0.80	26.81	18.25	29.60	32.30	-1.01	Down	8.78E-01
SM(d50:6+pO)	932.7	4.06	15.36	3.30	3.30	2.63	14.75	13.51	18.25	3.79	Up	1.00E-04
SM(d51:4)	934.8	22.64	15.36	1.53	1.53	19.29	14.75	24.63	18.25	-1.47	Down	1.00E-04
SM(d52:2)	952.8	29.12	29.35	0.69	0.69	28.16	25.56	30.47	31.96	1.01	Up	6.75E-01
SM(d52:2+pO)	968.8	22.12	23.35	0.90	0.90	20.45	15.59	24.00	26.58	1.06	Up	1.06E-02
SM(d53:5)	960.8	19.22	15.36	3.00	3.00	14.88	14.75	23.33	18.25	-1.25	Down	1.10E-03
SM(d54:3+pO)	994.8	13.65	15.36	8.53	8.53	2.63	14.75	23.58	18.25	1.13	Up	3.72E-01
SM(d56:3)	1007	15.68	15.36	6.90	6.90	2.63	14.75	23.29	18.25	-1.02	Down	2.46E-02
So(d12:0)	217.2	23.08	23.39	2.52	1.58	20.22	21.66	28.37	27.31	1.01	Up	7.28E-01
So(d12:0+pO)	233.2	21.54	23.33	2.30	1.53	18.25	21.68	26.82	26.82	1.08	Up	3.74E-02
So(d13:0+pO)	247.2	21.55	22.03	3.43	4.07	17.39	15.07	27.77	29.73	1.02	Up	7.63E-01
So(d14:0)	245.2	25.49	24.08	3.44	2.22	20.70	22.23	31.58	30.37	-1.06	Down	3.47E-01
So(d14:0+pO)	261.2	20.44	21.67	6.19	2.94	11.23	15.15	28.95	27.87	1.06	Up	9.75E-01
So(d14:1)	243.2	20.38	21.83	6.22	2.97	11.03	15.15	28.92	27.88	1.07	Up	9.76E-01
So(d14:1+hO)	259.2	15.03	15.36	5.28	0.94	2.63	14.75	22.76	18.25	1.02	Up	5.25E-01
So(d15:0)	259.3	20.81	24.23	2.46	2.05	18.34	21.93	26.85	28.46	1.16	Up	5.00E-04
So(d15:0+pO)	275.2	24.89	24.27	2.61	2.83	20.95	22.01	29.20	30.92	-1.03	Down	4.79E-01
So(d16:0)	273.3	30.53	30.05	3.47	2.03	25.98	27.44	35.26	35.39	-1.02	Down	8.78E-01
So(d16:0+pO)	289.3	22.47	25.11	2.51	2.44	19.93	22.77	27.86	31.66	1.12	Up	1.88E-02
So(d16:1)	271.3	23.29	26.27	1.93	2.30	20.78	23.65	28.07	32.20	1.13	Up	3.00E-03
So(d17:0)	287.3	22.75	23.97	2.57	2.06	19.95	21.46	27.16	28.32	1.05	Up	1.76E-01
So(d17:0+pO)	303.3	23.07	26.21	2.32	2.82	20.98	23.09	28.27	30.51	1.14	Up	6.20E-03
So(d17:1)	285.3	22.99	27.19	2.14	2.54	20.01	23.77	27.23	31.03	1.18	Up	3.00E-04
So(d18:0)	301.3	31.68	30.48	2.84	2.10	28.42	27.35	36.45	34.38	-1.04	Down	2.60E-01
So(d18:0+pO)	317.3	31.71	30.04	2.55	2.53	28.40	27.20	35.78	36.45	-1.06	Down	1.17E-01
So(d18:1)	299.3	31.56	30.64	2.60	3.12	28.63	26.53	35.58	36.45	-1.03	Down	4.55E-01
So(d18:1+hO)	315.3	26.89	25.47	3.80	2.56	23.13	22.41	34.17	31.14	-1.06	Down	6.95E-01
So(d18:2)	297.3	26.88	25.44	4.26	2.48	21.61	22.95	33.89	31.62	-1.06	Down	7.40E-01
So(d20:0)	329.3	27.47	27.12	3.04	2.04	24.23	24.74	32.62	31.91	-1.01	Down	7.48E-01
So(d20:0+pO)	345.3	26.02	27.30	3.28	2.88	22.47	24.58	32.02	33.89	1.05	Up	3.25E-01
So(d20:1)	327.3	25.32	26.97	3.70	2.84	20.90	24.27	31.83	33.46	1.07	Up	3.16E-01

So(d20:1+hO)	343.3	21.55	24.25	0.73	1.13	20.48	23.08	22.84	27.47	1.13	Up	1.00E-04
So(d20:2)	325.3	17.40	22.07	6.49	1.54	4.21	20.94	27.76	26.76	1.27	Up	1.16E-02
So(d22:0)	357.4	26.40	27.91	3.21	4.20	21.78	22.43	31.91	33.29	1.06	Up	3.49E-01
So(d22:0+pO)	373.4	28.77	26.65	2.00	2.18	25.31	23.68	32.77	32.40	-1.08	Down	6.20E-03
So(d22:1)	355.3	28.59	29.42	2.11	2.17	24.92	27.02	32.70	33.63	1.03	Up	3.62E-01
So(d22:1+hO)	371.3	21.95	24.57	1.82	1.50	19.16	22.88	26.35	27.41	1.12	Up	1.10E-03
So(d22:2)	353.3	22.22	18.21	2.40	4.22	19.50	14.86	27.84	25.77	-1.22	Down	1.63E-02
So(d24:0)	385.4	25.48	27.83	3.82	3.05	21.04	23.17	32.08	32.48	1.09	Up	1.16E-01
So(d24:0+pO)	401.4	24.00	24.45	1.85	2.06	22.20	22.76	28.50	30.47	1.02	Up	3.72E-01
So(d25:0)	399.4	20.43	23.29	5.22	6.53	12.36	15.00	30.54	31.45	1.14	Up	4.13E-01
So(d25:0+pO)	415.4	10.20	25.37	10.94	5.15	2.63	15.11	31.30	31.87	2.49	Up	4.00E-03
SoG1(d12:0)	379.26	15.62	21.35	4.24	1.19	5.89	18.25	21.36	23.03	1.37	Up	1.00E-04
SoG1(d15:0)	421.30	13.64	22.41	4.89	1.21	2.63	20.95	18.39	25.27	1.64	Up	1.00E-04
SoG1(d16:0)	435.32	16.10	23.23	5.62	1.32	2.63	21.51	21.20	26.07	1.44	Up	1.00E-04
SoG1(d16:0+pO)	451.31	29.78	29.02	0.73	2.48	29.12	26.69	31.33	35.58	-1.03	Down	6.88E-02
SoG1(d16:1)	433.30	15.25	15.36	5.20	0.94	4.21	14.75	22.97	18.25	1.01	Up	5.58E-01
SoG1(d17:0)	449.34	18.00	23.52	2.88	0.92	10.51	22.14	21.88	25.52	1.31	Up	1.00E-04
SoG1(d17:1+hO)	463.31	15.22	21.61	4.93	1.25	5.89	18.25	21.02	23.00	1.42	Up	2.00E-04
SoG1(d18:0+pO)	479.35	19.23	23.70	2.22	1.39	15.36	22.06	22.63	26.10	1.23	Up	1.00E-04
SoG1(d18:1)	461.34	21.30	24.99	0.98	1.61	19.69	22.99	22.54	27.66	1.17	Up	1.00E-04
SoG1(d20:1+hO)	505.36	16.49	23.49	7.66	2.11	2.63	18.25	23.35	26.70	1.42	Up	1.50E-03
SoG1(d20:2)	487.35	21.63	24.09	1.21	2.34	19.93	18.25	23.38	27.46	1.11	Up	5.30E-03
SoG1(d22:1+hO)	533.39	19.09	19.19	1.54	3.21	16.99	14.75	21.97	22.32	1.01	Up	6.89E-01

✣Lipid identification and relative quantification were performed using LipidSearch 4.1 software (Thermo). The search criteria were as follows: product search; parent m/z tolerance 5 ppm; product m/z tolerance 5ppm; quantification: m/z tolerance 5 ppm, retention time tolerance 0.5 min. Cer = Ceramides; SM = sphingomyelins; So = sphingosine; SoG1 = glucosylsphingosine. * p-values for t-tests and Wilcoxon rank-sum results. Data presented have been log2 transformed.

Supplemental Donor Information

The typical donor information that received followed by receipt of tissue. The diagnosis is usually based on open angle and consecutive static perimetry.

We also consult with attending Ophthalmologist/Physician when possible.

Overview #[redacted] Eye: Right

Central 24-2 Threshold Test

Threshold Graytone | Threshold (dB) | Total Deviation | [redacted] Pattern Deviation

A SITA-Standard GHT: Within normal limits 6.3 mm

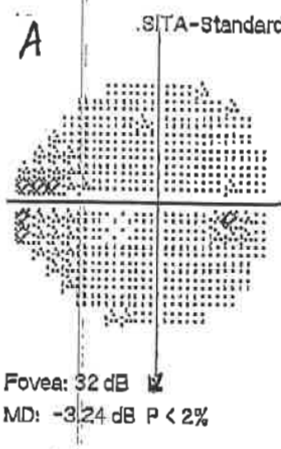

			27	27	27	25		
		28	29	25	26	28	27	
	24	27	28	27	27	28	28	28
20	20	25	27	30	29	29	25	29
20	22	29	31	30	30	29	16	27
	23	28	30	30	29	29	27	30
		28	29	29	27	30	27	
			25	26	26	27		

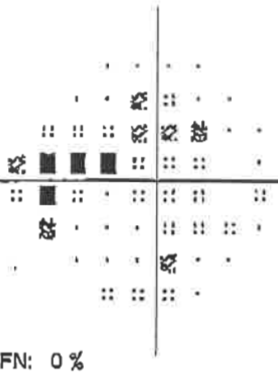

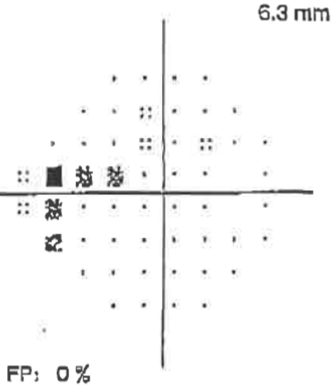

Fovea: 32 dB
MD: -3.24 dB P < 2%

FL: 0/14
PSD: 1.99 dB P < 10%

FN: 0 %

FP: 0 %

B SITA-Standard GHT: Outside normal limits 6.3 mm 20/30

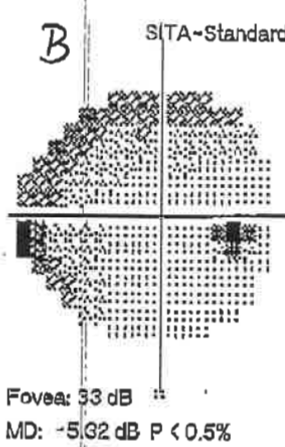

			17	16	18	16		
		18	22	19	23	24	21	
	17	23	24	27	25	26	26	27
20	20	25	27	26	29	28	27	28
<0	21	24	26	29	29	30	<0	28
	20	25	28	30	30	29	28	27
		21	27	28	28	30	28	
			26	27	29	26		

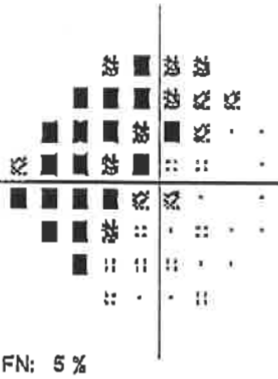

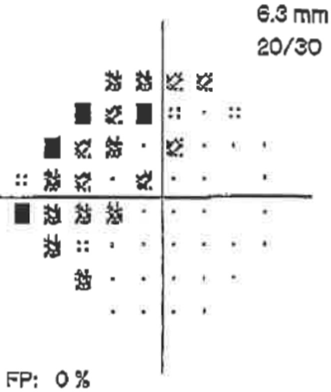

Fovea: 33 dB
MD: -5.32 dB P < 0.5%

FL: 2/16
PSD: 3.74 dB P < 0.5%

FN: 5 %

FP: 0 %

C SITA-Standard GHT: Outside normal limits

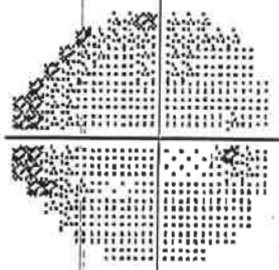

			21	20	21	23		
		22	27	24	25	26	27	
	21	29	29	26	26	26	28	30
18	23	27	27	28	29	29	25	29
17	22	26	29	30	31	31	18	27
	20	25	31	30	30	30	28	28
		28	28	28	28	30	28	
			26	27	28	29		

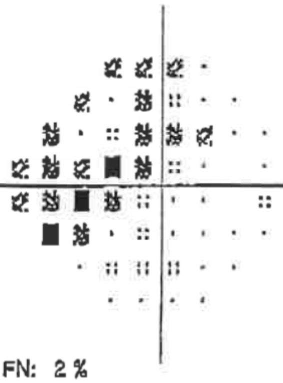

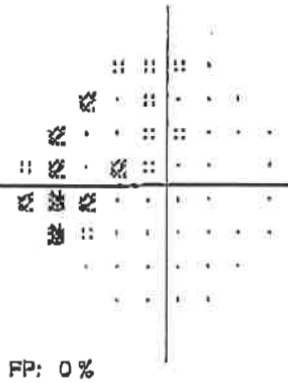

Fovea: 34 dB
MD: -3.63 dB P < 1%

FL: 0/16
PSD: 2.29 dB P < 5%

FN: 2 %

FP: 0 %

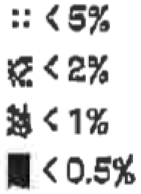

Overview #[redacted] Eye: Right

Central 24-2 Threshold Test

[redacted]

Threshold Graytone | Threshold (dB) | Total Deviation | Pattern Deviation

D

SITA-Standard GHT: Borderline/General Reduction

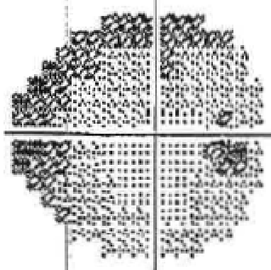

			18	19	19	18		
		19	21	22	19	23	23	
	17	22	22	22	21	22	22	24
16	19	25	24	25	28	27	19	23
13	17	23	26	27	28	27	8	22
	20	24	25	23	28	25	25	24
		22	24	25	28	25	23	
			24	24	24	24		

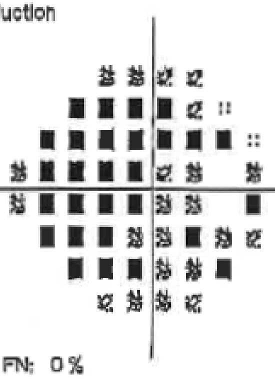

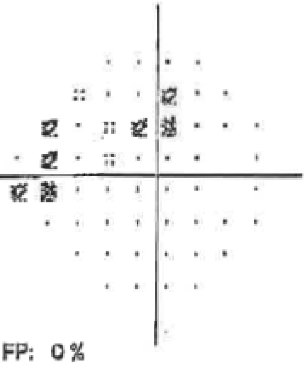

Fovea: 30 dB ■ FL: 1/16 FN: 0 % FP: 0 %

MD: -7.10 dB P < 0.5% PSD: 2.18 dB P < 5%

E

SITA-Standard GHT: Borderline/General Reduction

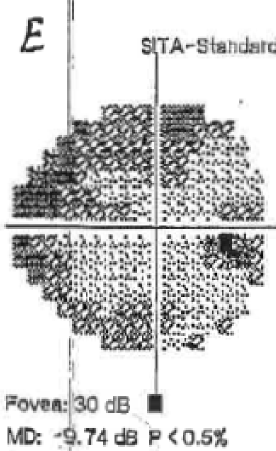

			16	17	13	13		
		16	18	19	17	20	22	
	11	20	23	21	19	22	24	21
13	15	20	18	22	24	23	19	20
12	20	22	23	25	25	23	10	20
	17	22	25	28	24	24	21	20
		19	22	18	20	23	21	
			17	19	21	20		

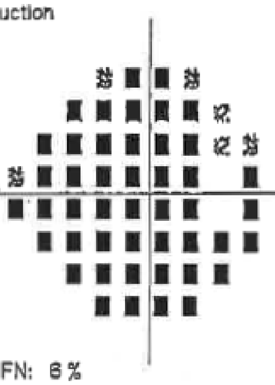

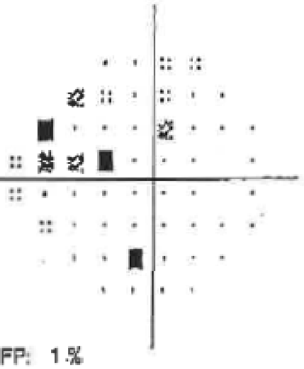

Fovea: 30 dB ■ FL: 2/16 FN: 6 % FP: 1 %

MD: -9.74 dB P < 0.5% PSD: 2.54 dB P < 2%

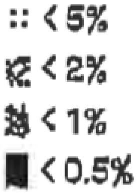

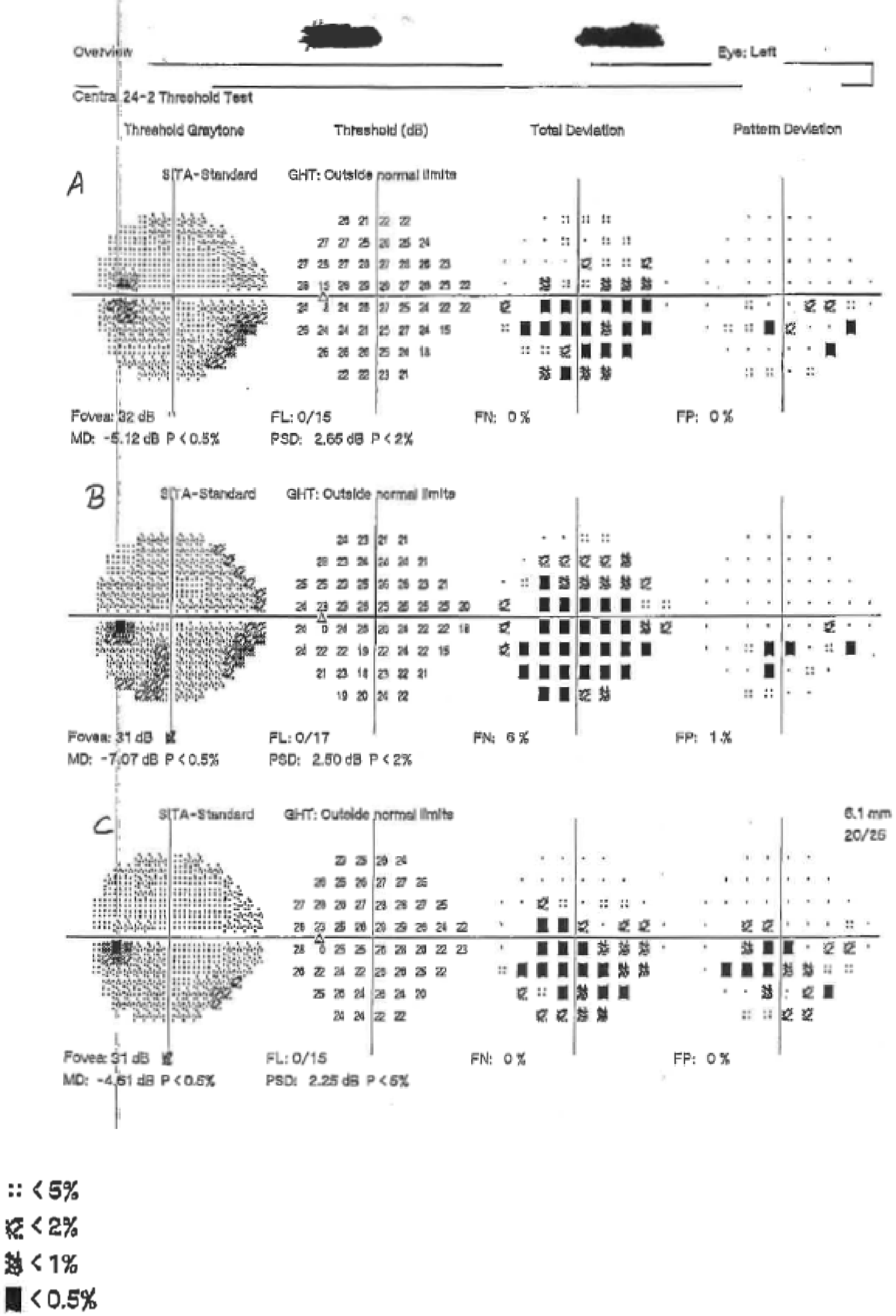
Overview
Eye: Left
Central 24-2 Threshold Test
Threshold Graytone
Threshold (dB)
Total Deviation
Pattern Deviation
A
SITA-Standard
GHT: Outside normal limits
Fovea: 32 dB
MD: -5.12 dB P < 0.5%
FL: 0/15
PSD: 2.65 dB P < 2%
FN: 0 %
FP: 0 %
B
SITA-Standard
GHT: Outside normal limits
Fovea: 31 dB
MD: -7.07 dB P < 0.5%
FL: 0/17
PSD: 2.50 dB P < 2%
FN: 6 %
FP: 1 %
C
SITA-Standard
GHT: Outside normal limits
6.1 mm
20/25
Fovea: 31 dB
MD: -4.61 dB P < 0.5%
FL: 0/15
PSD: 2.25 dB P < 5%
FN: 0 %
FP: 0 %
< 5%
< 2%
< 1%
< 0.5%

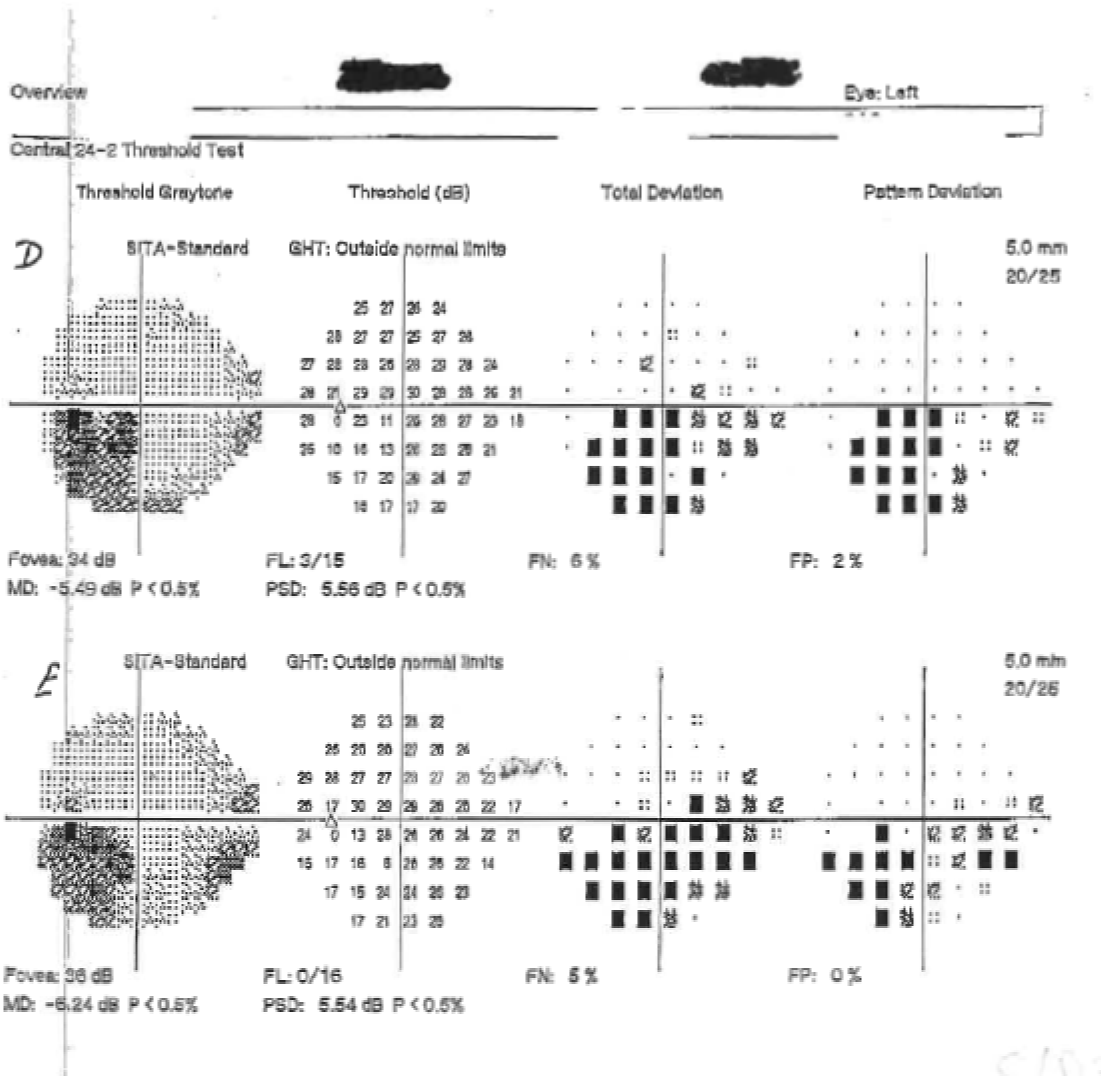

Overview [redacted] [redacted]
Eye: Left
Central 24-2 Threshold Test
Threshold Graytone
Threshold (dB)
Total Deviation
Pattern Deviation
D
SITA-Standard
GHT: Outside normal limits
5.0 mm
20/25
Fovea: 34 dB
FL: 3/15
FN: 6 %
FP: 2 %
MD: -5.49 dB P < 0.5%
PSD: 5.56 dB P < 0.5%
E
SITA-Standard
GHT: Outside normal limits
5.0 mm
20/25
Fovea: 36 dB
FL: 0/16
FN: 5 %
FP: 0 %
MD: -6.24 dB P < 0.5%
PSD: 5.54 dB P < 0.5%

∷ < 5%

< 2%

< 1%

< 0.5%

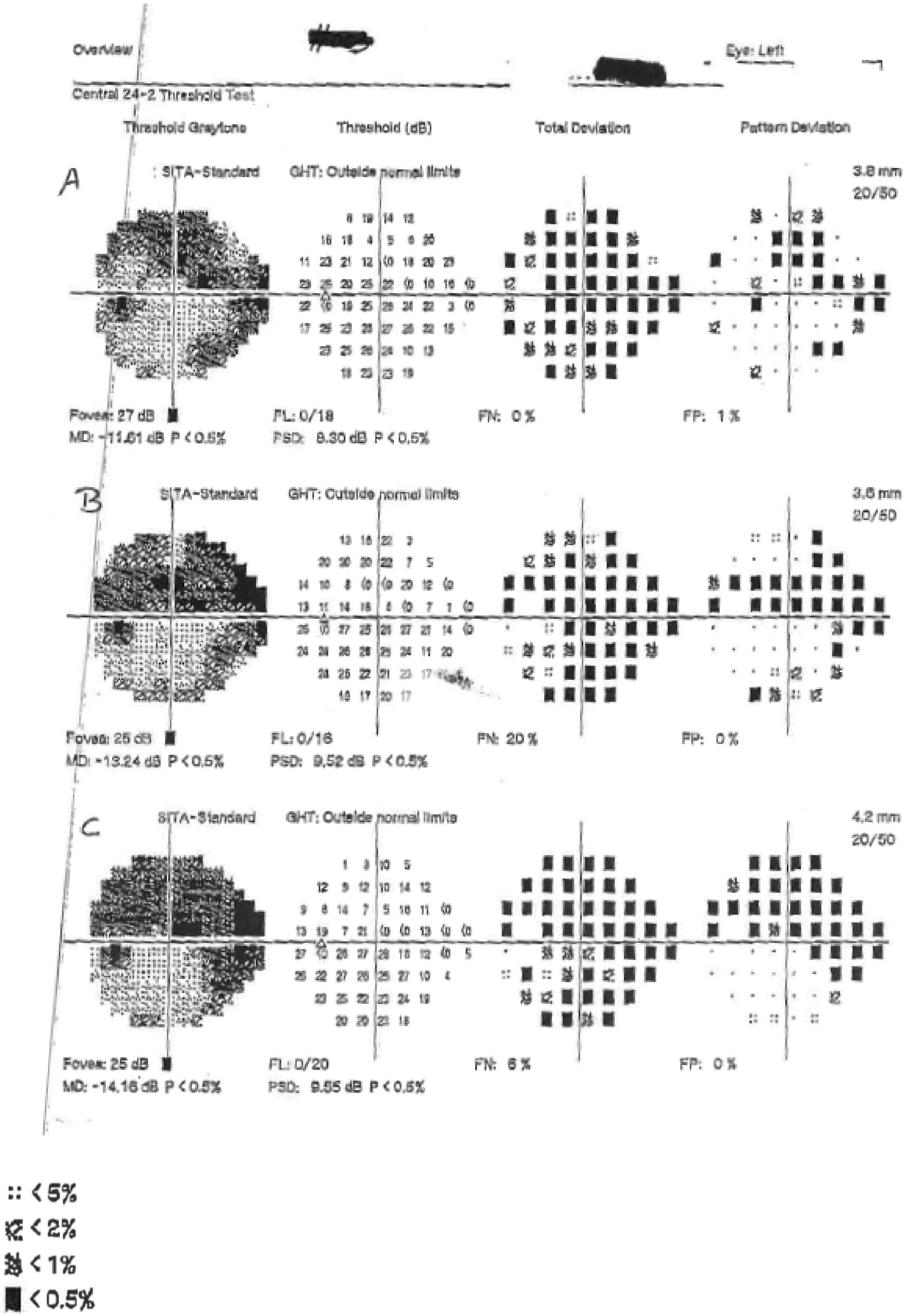
Overview
Eye: Left
Central 24-2 Threshold Test
Threshold Graytone
Threshold (dB)
Total Deviation
Pattern Deviation
A
SITA-Standard
GHT: Outside normal limits
3.8 mm
20/50
Fovea: 27 dB
MD: -11.61 dB P < 0.5%
FL: 0/18
PSD: 8.30 dB P < 0.5%
FN: 0 %
FP: 1 %
B
SITA-Standard
GHT: Outside normal limits
3.6 mm
20/50
Fovea: 25 dB
MD: -13.24 dB P < 0.5%
FL: 0/16
PSD: 9.52 dB P < 0.5%
FN: 20 %
FP: 0 %
C
SITA-Standard
GHT: Outside normal limits
4.2 mm
20/50
Fovea: 25 dB
MD: -14.16 dB P < 0.5%
FL: 0/20
PSD: 9.55 dB P < 0.5%
FN: 6 %
FP: 0 %
:: < 5%
< 2%
< 1%
< 0.5%

Overview [redacted] [redacted] Eye: Left

Central 24-2 Threshold Test

Threshold Graytone | Threshold (dB) | Total Deviation | Pattern Deviation

D SITA-Standard GHT: Outside normal limits 4.2 mm 20/50

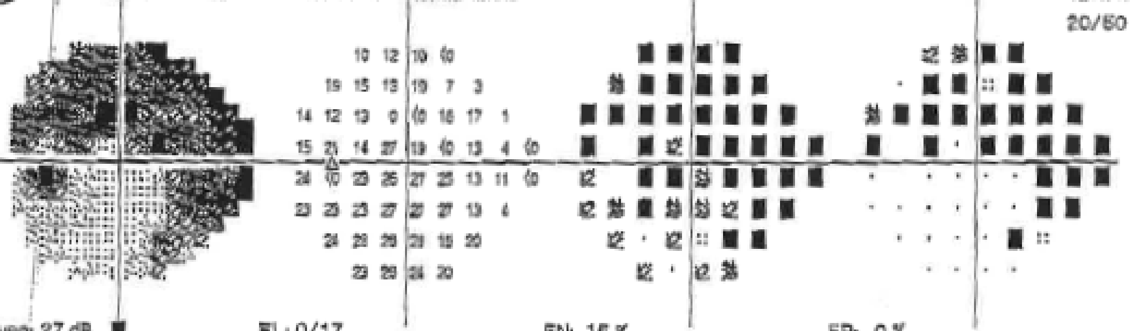

Fovea: 27 dB ■ FL: 0/17 FN: 15 % FP: 0 %

MD: -12.55 dB P < 0.5% PSD: 9.26 dB P < 0.5%

E SITA-Standard GHT: Outside normal limits 4.2 mm 20/50

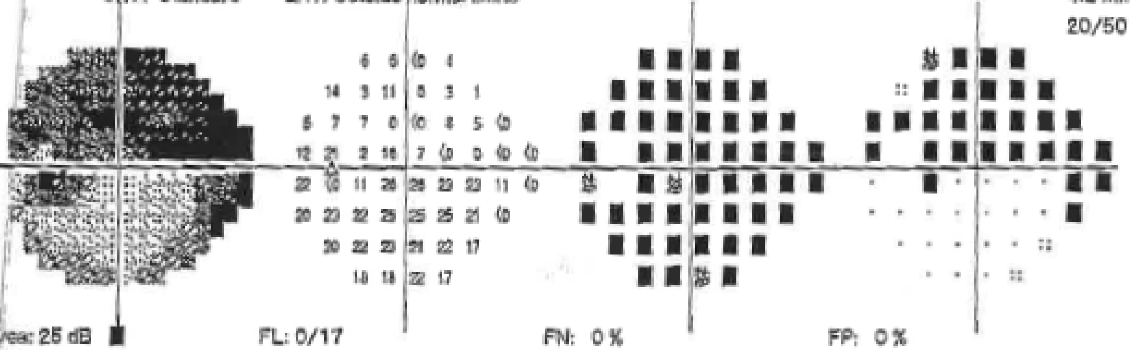

Fovea: 25 dB ■ FL: 0/17 FN: 0 % FP: 0 %

MD: -16.32 dB P < 0.5% PSD: 9.85 dB P < 0.5%

:: < 5%

< 2%

< 1%

■ < 0.5%

Min. = 1
Inter = 9
Comp = 12

EXAM SHEET

Patient [redacted] Acct. Name ______ Birthdate [redacted] Hm.Ph. ______

Interview Sheets Comments: ______

[redacted]

Date 12-29-17 Time

Cur Rx	SPH	CYL	AXIS	ADD
R	pl.	-1.25	080	+2.25
L	+0.75	-1.50	170	

Lens Type/Comments: ows & PAL — Broken

[redacted]

Ref. by

Orientation: A O-C-D E

Mood: A (C) D E

Amb: Has Amb.

[redacted]

Blood Pressure ____/____

[redacted]

R	
L	10/ 125

Rx
OD
OS

Add ______ Prism OD ______ OS ______

[redacted]

CC: Blurred vision. Dull pain. PAL unclear [illegible] — Family Present Y or (N)

Name

PI/PAU of: — Relationship

Prev. Ex Rec: 13 glts — POA Y or N

Med Chart Review: H&P S.S. (MDS) Nurses Service Request Old Chart

Nurse/Family comments:

Rx Medication		Systemic DX:
Alprazolam	Prochlorperazine	COPD
Amitriptyline	Quetiapine	ASHD
Cosentyx	Sertraline	CAD
Clopidogrel	Tizanidine	HTN
Fluoxetine	Zofran	Seizures
Folic Acid	Other: Lipsyl	Anxiety
Furosemide	Allergies	SS Complaints
Hydrocodone	DA: NKDA:	
Norco	Depakote	UTI
Ondansetron	[illegible]	
Pr Chlor	Aspirin [illegible]	

R X — L X

2+ pal blephs

Lids: cl — cl

cj

Cornea: cl — cl

AC: I II III (IV) — I II III (IV)

Iris: hazel — hazel

Lens: opacl — opacl

Vitreous

☐ Open 360° pigment — ☐ Open 360° pigment

PVD ou

3/3 + D. cup + D. 3/3

Disc/Rim

Color / — Color /

Contour / — Contour /

Macula: mottled

5 AV 5
Vessels

glts: (1) 2 - (3) 4 - 5 - 6 - 7 OU
(20D) FCL - 90D - 14D - 3MIR - QUAD
DIRECT - GONIO

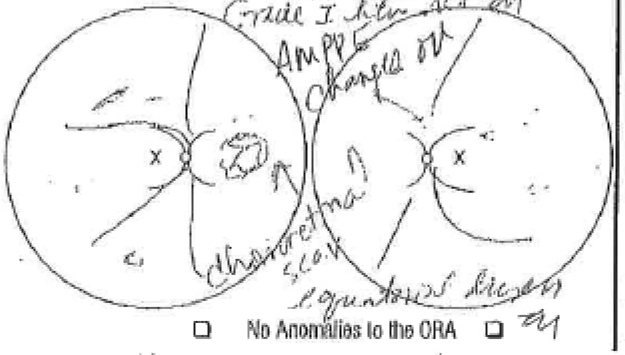

☐ No Anomalies to the ORA ☐ ou

Assessment/Dx: (1) PC/OL ou
(2) Blepharitis ou
(3) chorioretinal scarring OD
(4) Equatorial drusen
(5) [illegible] htn retinop. [illegible]

Plan/PE: Select [illegible] 1720 and [illegible] when eligible. Tighten [illegible] lids. RTC 1 year full

Plan: Orders for nursing staff

[redacted]

PE 1 2 3 4 5 6 7 8 9 10 11 12 13 14 [redacted]

PS 8105b Rev. 3/16
